# Synthesis of Highly Functionalized Bismacycles via Post-Transmetallation Modification of Arylboronic Acids

**DOI:** 10.1021/acs.joc.3c00361

**Published:** 2023-07-12

**Authors:** Sudheesh T. Sivanandan, Benjamin Owen, Patrick J. Guiry, Liam T. Ball

**Affiliations:** † School of Chemistry, University of Nottingham, Nottingham NG7 2RD, U.K; ‡ Centre for Synthesis and Chemical Biology, School of Chemistry, University College Dublin, Belfield Dublin 4, Ireland

## Abstract

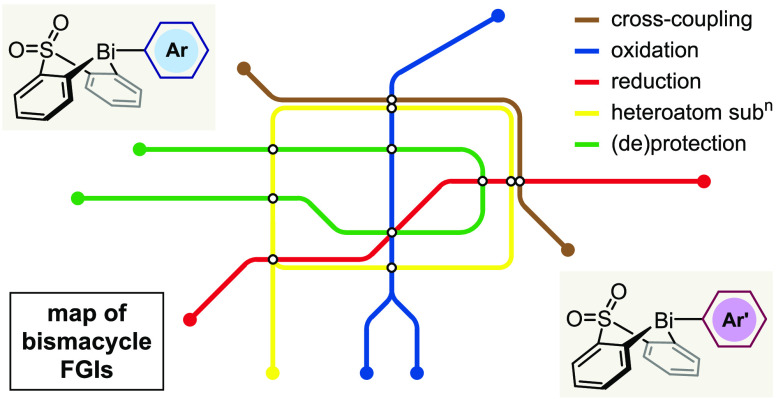

Bismacycles featuring a sulfone-bridged scaffold have recently been developed as versatile and convenient electrophilic arylating agents. Here, we report that the exocyclic aryl group, which is ultimately transferred to a nucleophilic coupling partner, can be functionalized through cross-coupling, heteroatom substitutions, oxidations and reductions, and protecting group manipulations. This “postsynthetic modification” approach provides concise and divergent access to complex aryl bismacycles. The utility of the functionalized bismacycles in electrophilic arylation of C–H and O–H bonds is demonstrated.

## Introduction

**Scheme 1:**
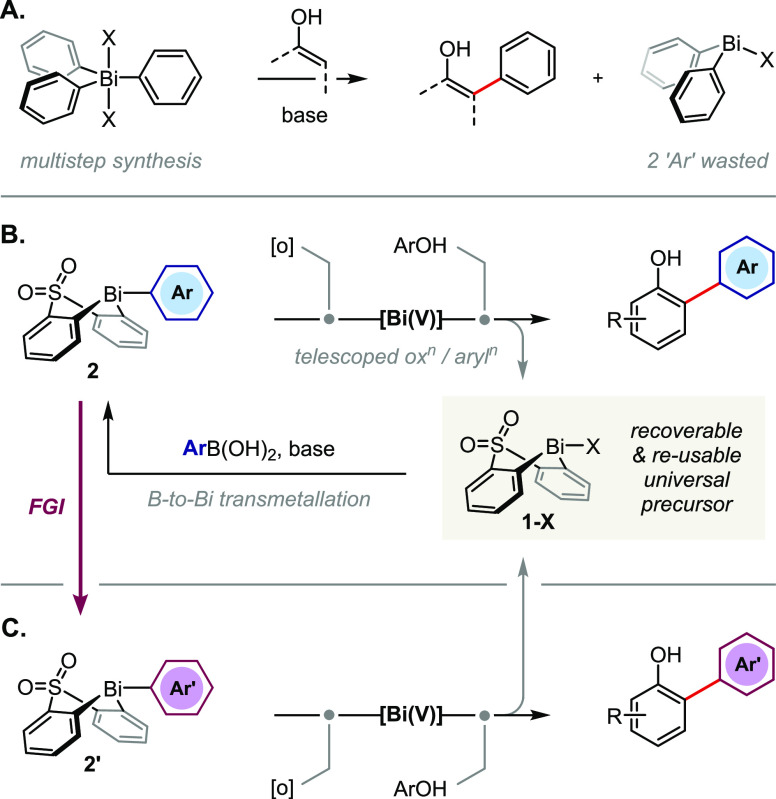
Bi(V)-Mediated Arylation

Couplings of C-, N-, and O-nucleophiles with aryl electrophiles are among the most valuable transformations in organic synthesis. Despite being comparatively underutilized, electrophilic arylation strategies based on hypervalent main group elements^1−7^ represent powerful, and often complementary, alternatives to ubiquitous approaches such as transition metal catalysis or S_N_Ar.^8−10^ For example, Barton,^11−18^ Dodonov,^19,20^ and many others^21−25^ have demonstrated the utility of triarylbismuth(V) compounds as potent C–H arylating agents for phenol and enol nucleophiles (Scheme 1A). While the stability and low toxicity of triarylbismuth reagents in both the +3 and +5 oxidation states have undoubtedly contributed to their appeal, the field has traditionally suffered from significant practical issues that derive primarily from the homoleptic nature of simple arylbismuth species.

First, the synthesis of triarylbismuth(V) compounds typically requires multistep sequences in which the aryl moieties are introduced using Grignard reagents. The reliance on such a reactive class of organometallic reagents restricts functional group compatibility and ultimately limits the diversity of aryl groups that can be installed. In 1926, Adams reported a solution to this challenge in which triarylbismuth(V) reagents were functionalized through (1) electrophilic aromatic nitration or (2) oxidation of benzylic methyl substituents to carboxylic acids.^26^ Postsynthetic functionalizations of triarylbismuth reagents in the +3 oxidation state are somewhat better explored,^27−29^ with a particularly detailed study published by Gagnon in 2016.^30^

However, these strategies do not address the second issue associated with homoleptic bismuth reagents: atom economy. Not only is the metal rarely recoverable, but the transfer of only one aryl moiety to the nucleophile also results in the remaining two aryl groups being wasted. In theory, this latter challenge could be addressed using heteroleptic triarylbismuth reagents bearing two low-value aryl groups that do not transfer, analogous to the “dummy aryl” concept that has been used to great effect in diaryliodonium chemistry.^31,32^ However, selective transfer of a specific aryl group from a heteroleptic triarylbismuth(V) reagent has not been well explored and has been met with only limited success.^16,33−36^ Furthermore, the synthesis of heteroleptic triarylbismuthanes is nontrivial, being both enabled and hindered by aryl scrambling.^37^

In 2020, we reported a solution to the dual challenges of accessibility and atom economy.^38,39^ Using a general and stable bismuth(III) precursor based on Suzuki′s sulfone-bridged bismacycle,^40^ we developed a telescoped procedure consisting of B-to-Bi transmetallation, followed by oxidation and *ortho*-selective C–H arylation of a phenol (Scheme 1B). Crucially, the valuable aryl moiety is installed at bismuth in a modular fashion from 1.1 equiv of an arylboronic acid (**1-X** → **2**). The use of Grignard reagents is therefore avoided, which benefits the safety, convenience, and functional group compatibility of the process. Following oxidation to Bi(V), the subsequent electrophilic arylation proceeds with complete selectivity for transfer of the exocyclic aryl moiety (*Cf*. acyclic heteroleptic bismuthanes),^16,33−36^ and the resulting bismacycle co-product can be recovered and reused in excellent yield. We have subsequently adapted our methodology to the *meta*-selective C–H arylation of phenols,^41^ the α-arylation of cyclic and polyfluoroalkyl diones,^42^ and the *O*-selective arylation of 2- and 4-pyridones.^43^ Contemporaneously with our initial report, Cornella demonstrated the use of a structurally related sulfoximine-bridged bismacycle for catalytic C_Ar_–F formation^44^ and subsequently used substituted sulfone-bridged bismacycles in catalytic C_Ar_-OTf formation^45^ and sulfonyl fluoride synthesis.^46^

The utility of our bismuth-mediated arylation strategy hinges on the B-to-Bi transmetallation process (**1-X** → **2**, Scheme 1B), which we have so far demonstrated with over 65 distinct examples spanning a sterically and electronically diverse range of arylboronic acids.^38,41−43^ However, while extremely enabling, the transmetallation comes with an implicit limitation: the requisite boronic acid must be accessible, either commercially or by de novo synthesis.

We anticipated that functional group interconversions (FGIs) at the exocyclic aryl group after its installation on bismuth (**2** → **2′**, Scheme 1C) would provide a convenient solution to this issue. Furthermore, as a general strategy, “post-transmetallation modification” would also enable (1) the rapid structural diversification of a common aryl bismacycle precursor for library synthesis, and (2) installation of aryl moieties for which direct B-to-Bi transmetallation is slow, such as from electron-poor and sterically hindered boronic acids.^42,43^ Given that the B-to-Bi transmetallation is compatible with numerous synthetically versatile functional groups, including halides, alkenes, and carbonyls, there is a huge scope for the transformations that could potentially be achieved. However, the overall success of the strategy requires not only that the desired transformation proceeds efficiently, but also that the weak^47^ Bi–C bonds remain intact during the reaction and any subsequent purifications.

Herein, we report the post-transmetallation modification of aryl bismacycles as a concise route to highly functionalized electrophilic arylating agents. The concept is illustrated using some of the most prevalent reaction types in drug discovery,^8−10^ including cross-coupling, heteroatom functionalization, oxidations and reductions, and protecting group manipulations. In this way, we demonstrate a highly enabling extension to the growing toolbox of organobismuth chemistry.

## Results and Discussion

**Scheme 2:**
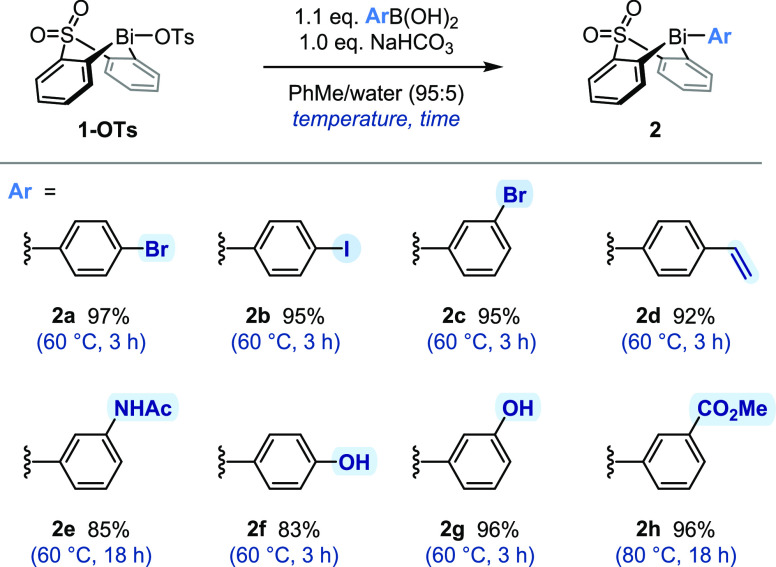
Synthesis of Aryl Bismacycles via B-to-Bi Transmetallation

In preparation for our studies, a library of aryl bismacycle substrates **2a**–**h** was synthesized via B-to-Bi transmetallation from the corresponding arylboronic acid (Scheme 2). The bismacycles were isolated as bench-stable solids in good yield following a simple aqueous workup.

**Scheme 3:**
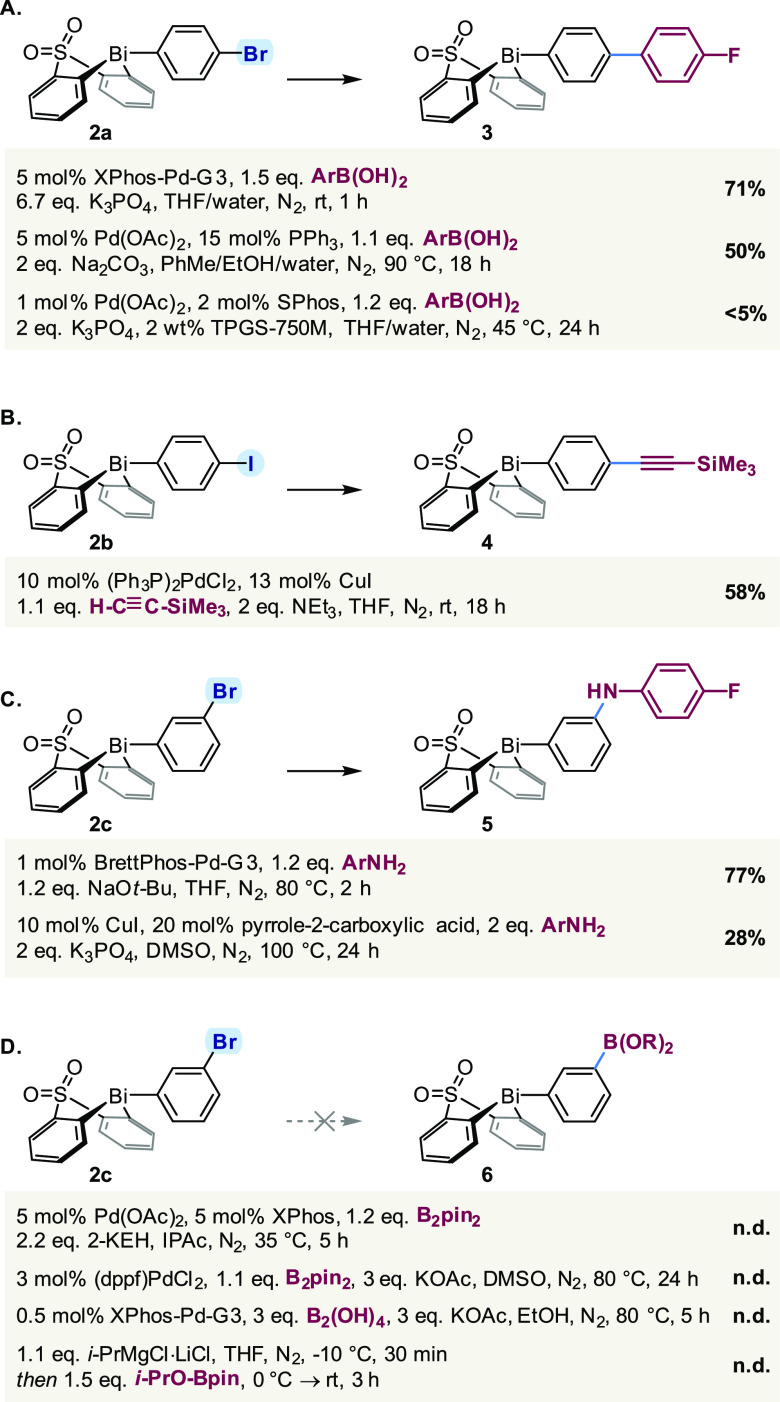
Pd-Catalyzed Cross-Couplings n.d., not detected; 2-KEH, potassium 2-ethylhexanoate.

Given their fundamental importance in contemporary synthesis, the first transformations to be considered were Pd-catalyzed cross-couplings. Suzuki–Miyaura cross-coupling of 4-bromophenyl bismacycle **2a** proceeded rapidly under Buchwald′s conditions,^48^ providing biaryl bismacycle **3** in good isolated yield (Scheme 3A). The same coupling can also be performed using PPh_3_ as a ligand under more conventional conditions, whereas the reaction in micellar solution^49−51^ proved unacceptably sluggish, presumably due to the poor solubility of **2a** in the aqueous reaction medium. Notably, products from cross-coupling of the Bi–C bonds were not observed under any of the conditions employed in this study. The apparent resistance of the aryl bismacycle to Bi-to-Pd transmetallation contrasts the extensive precedent for both Pd- and Cu-catalyzed couplings of homoleptic triarylbismuth reagents;^21,22,52^ this stark difference illustrates that one cannot simply extend the reactivity patterns established for homoleptic bismuth species to bismacyclic compounds, highlighting the importance of the present study.

The Sonogashira coupling of 4-iodophenyl bismacycle **2b** proved similarly successful, affording alkyne **4** in 58% isolated yield (Scheme 3B). As anticipated, the equivalent Sonogashira coupling with 4-bromophenyl bismacycle **2a** did not proceed (not shown), consistent with the low reactivity of aryl bromides under these conditions.^53^ Importantly, however, bismacycle **2a** was recovered unreacted, demonstrating its stability not only to a palladium catalyst but also to copper salts.

Moving beyond C–C bond formation, we were pleased to find that Buchwald–Hartwig amination of 3-bromophenyl bismacycle **2c** afforded the corresponding diarylamine **5** in a good isolated yield (Scheme 3C). Alternatively, the same product can be accessed under Cu catalysis (Scheme 3C),^54^ albeit in lower isolated yield. While application of the Pd-catalyzed protocol to regioisomeric 4-bromophenyl bismacycle **2a** also resulted in C–N cross-coupling, the (electron-rich) product proved unstable toward protodebismuthation during purification by chromatography on (basified) silica gel. This latter result highlights the potentially dichotomous stability of aryl bismacyclic species toward the functionalization conditions, here a strong base and a Pd catalyst, and subsequent manipulations, including purification.

Extending the scope of cross-couplings to Miyaura borylation proved unsuccessful under a range of conditions (Scheme 3D),^55−57^ in each case furnishing a complex mixture. Attempts to prepare pinacol boronate **6** (Scheme 3D; (OR)_2_ = pin) by sequential magnesium–halogen exchange/borylation also resulted in the complete consumption of bismacycle **2c** and formation of a complex mixture. Given that triarylbismuth species and arylboronates have been demonstrated to be compatible,^38,58^ this observation is attributed to the conditions required to install the boryl moiety. Indeed, all attempts to use reactive organometallic reagents in conjunction with the sulfone-bridged bismacycle proved unsuccessful (see the SI), consistent with the known ability of organometallic reagents to form bismuth(III) “ate” complexes prior to substituent exchange/decomposition.^59−61^

**Scheme 4:**
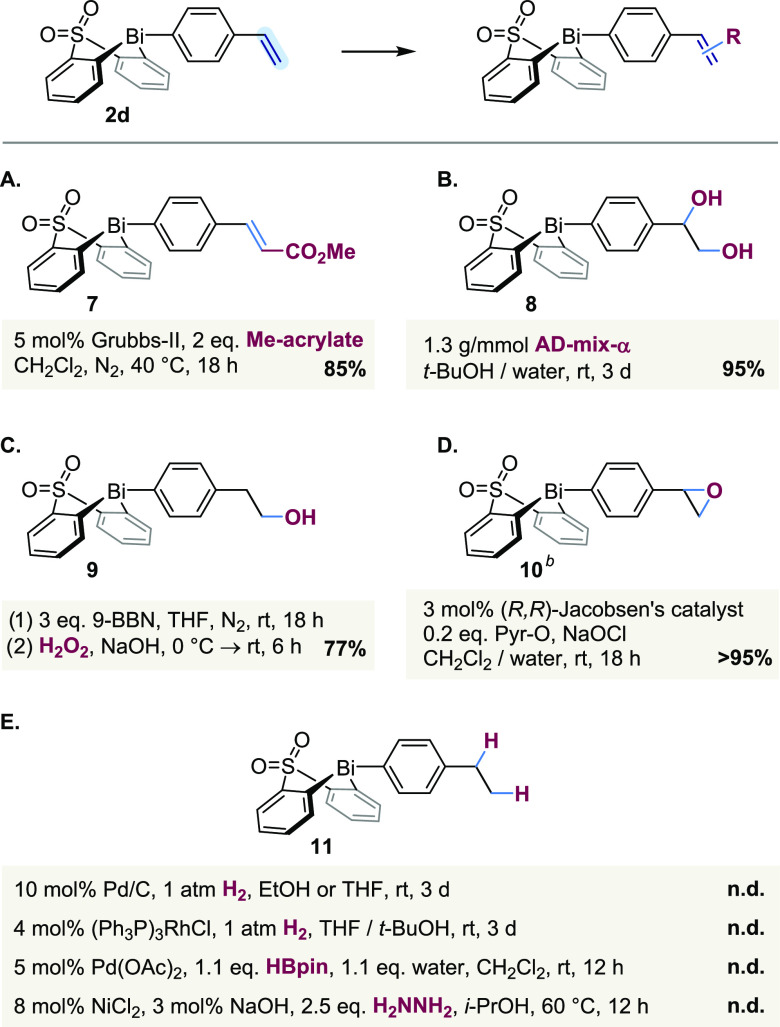
Alkene Functionalizations n.d., not detected; Pyr-O, pyridine *N*-oxide. Yield determined by ^1^H NMR spectroscopic analysis prior to purification.

To further explore the compatibility of our bismacycles with transition metal catalysis, we turned to functionalizations of styrenyl bismacycle **2d** (Scheme 4). Both cross-metathesis and Sharpless dihydroxylation proceeded smoothly (Scheme 4A,4B). The value of the “post-transmetallation modification” concept is illustrated by the fact that the boronic acid needed to prepare diol **8** directly via B-to-Bi transmetallation is only accessible in low yield (8%, from 4-styrenyl boronic acid),^62^ whereas performing dihydroxylation after transmetallation affords the same product in >90% over the two steps. Similarly, primary alcohol **9** was accessed via a hydroboration–oxidation sequence (Scheme 4C), the latter step of which is incompatible with the parent boronic acid.

While Jacobsen–Katsuki epoxidation^63^ did proceed quantitatively, as determined by ^1^H NMR spectroscopy (Scheme 4D), epoxide **10** proved extremely sensitive to isolation and therefore could not be obtained pure. Notably, the opposite chemoselectivity is observed with *m*CPBA, which we have previously demonstrated oxidizes the bismuth center to Bi(V) in preference to epoxidizing a pendant styrene^38^ or mediating Baeyer–Villiger rearrangement on a pendant formyl substituent.^41^

Although styrenyl bismacycle **2d** tolerates both oxidants (Scheme 4B) and reductants (Scheme 4C), not all redox processes were compatible. For example, only the unreacted starting material was recovered following attempted hydrogenation of **2d** with H_2_ and either Pd/C or Wilkinson′s catalyst (Scheme 4E), while complete decomposition was observed when using HBPin/Pd(OAc)_2_^64^ or hydrazine/NiCl_2_.^65^ Furthermore, all attempted functionalizations based on photoredox catalysis proved unsuccessful (see the SI), despite the stability of the bismacycle to irradiation with visible light.

**Scheme 5:**
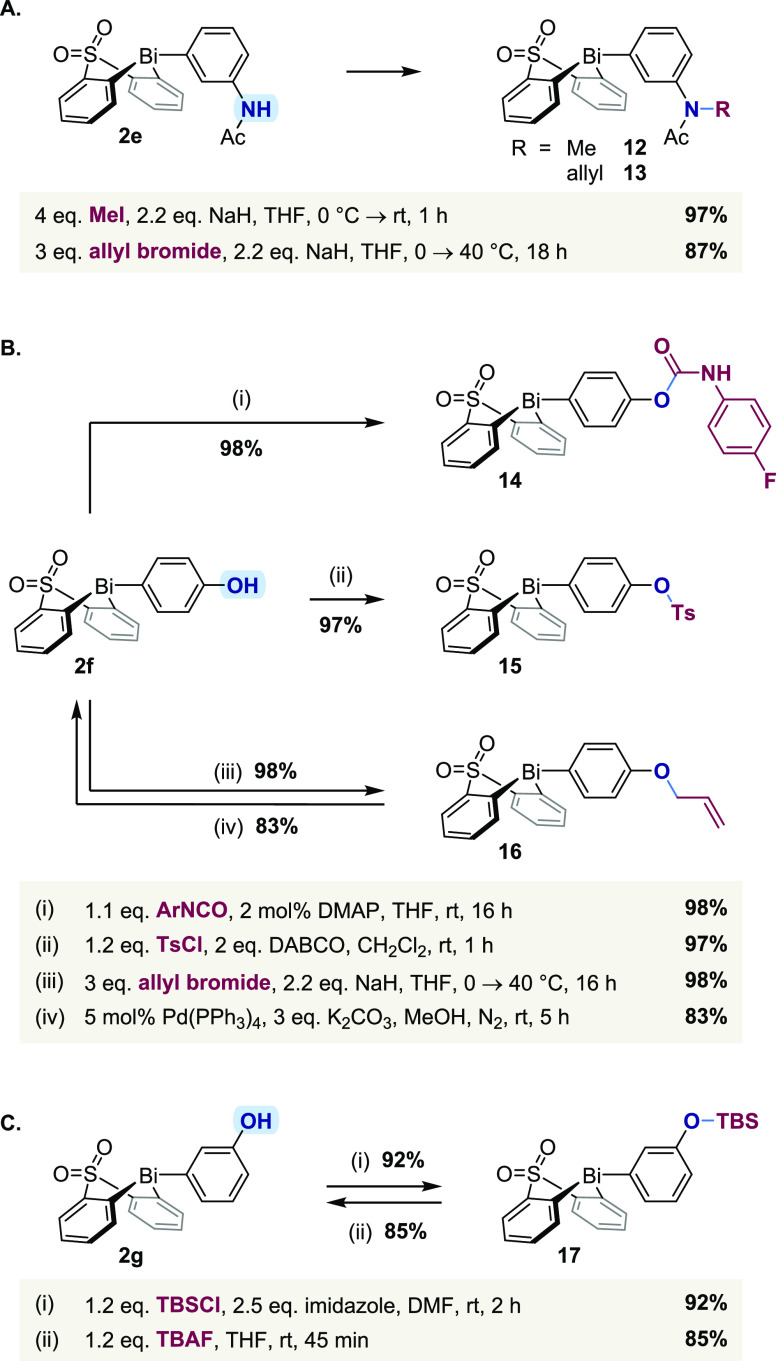
Electrophilic Substitutions

Attention was next turned to electrophilic substitutions at heteroatoms, which are among the most widely used transformations in pharmaceutical discovery chemistry.^8^ In this regard, 3-acetamidophenyl bismacycle **2e** underwent both *N*-methylation and *N*-allylation in excellent isolated yield (Scheme 5A), demonstrating the stability of the bismacyclic scaffold to a strong base.

Carbamoylation, tosylation, and allylation of 4-hydroxyphenyl bismacycle **2f** also proceeded in quantitative yields (Scheme 5B). In the latter case, initial attempts at *O*-allylation using K_2_CO_3_ in acetone led to cleavage of the exocyclic Bi–C_Ar_ bond via protodebismuthation, presumably due to the sensitivity of the very electron-rich phenoxy bismuth intermediate to the protic reaction environment (σ_*p*_(O^–^) = −0.81).^66^ However, the use of NaH as a base under aprotic conditions allowed for high-yielding allylation. Subsequent deprotection of allyl ether **16** proceeded smoothly under palladium catalysis to regenerate hydroxyphenyl bismacycle **2f**. Reproducibly high yields were achieved only when the deallylation was quenched as soon as full conversion had been reached (ca. 5 h); a prolonged reaction time (overnight) led to significant decomposition, again underscoring the sensitivity of electron-rich bismacycles to protodebismuthation.

Continuing the theme of protection/deprotection, *O*-silylation of 3-hydroxyphenyl bismacycle **2g** proceeded in excellent yield using *tert*-butyldimethylsilyl chloride (Scheme 5C). Subsequent desilylation of **17** with TBAF regenerated free phenol **2g**. Unlike the manipulations of regioisomeric **2f** (vide supra), protodebismuthation side-reactions were not observed for **2g**, consistent with the electron-withdrawing character of the *meta* hydroxy substituent (σ_*m*_(OH) = 0.12, vs σ_*p*_(OH) = −0.37).^66^

**Scheme 6:**
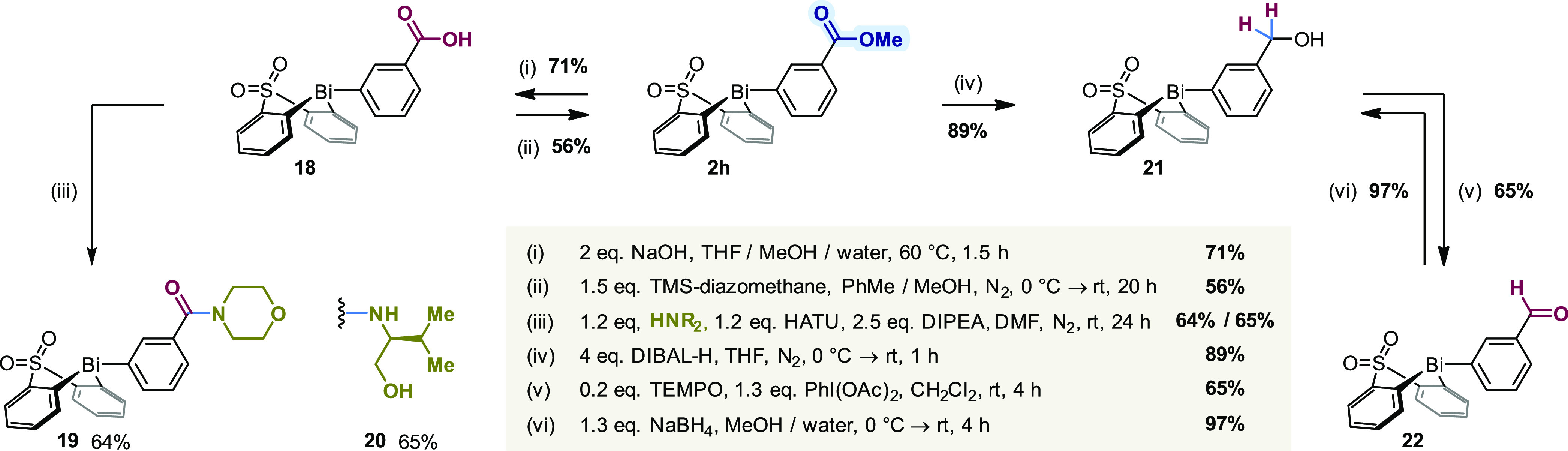
Functional Group Interconversions of Aryl Esters

Finally, we explored manipulations of ester **2h** (Scheme 6), which represent work-horse transformations in all aspects of synthesis. Following hydrolysis with NaOH, carboxylic acid **18** was isolated by precipitation upon careful acidification. Ester **2h** could be reformed by treatment of acid **18** with TMS-diazomethane; alternatively, amide coupling mediated by HATU afforded amides **19** and **20** in good yields. Reduction of ester **2h** with DIBAL-H gave access to primary benzylic alcohol **21**, which could then be converted selectively to aldehyde **22** by TEMPO-catalyzed oxidation with iodobenzene diacetate. The observed inertness of the bismacycle toward oxidation by I(III) is consistent with our earlier findings^38^ and stands in contrast to the reactivity reported previously for homoleptic triarylbismuth reagents.^67,68^ To demonstrate the compatibility of the bismacyclic scaffold with nucleophilic reductants, aldehyde **22** was subsequently reduced back to the primary alcohol **21** using sodium borohydride in nearly quantitative yield.

**Scheme 7:**
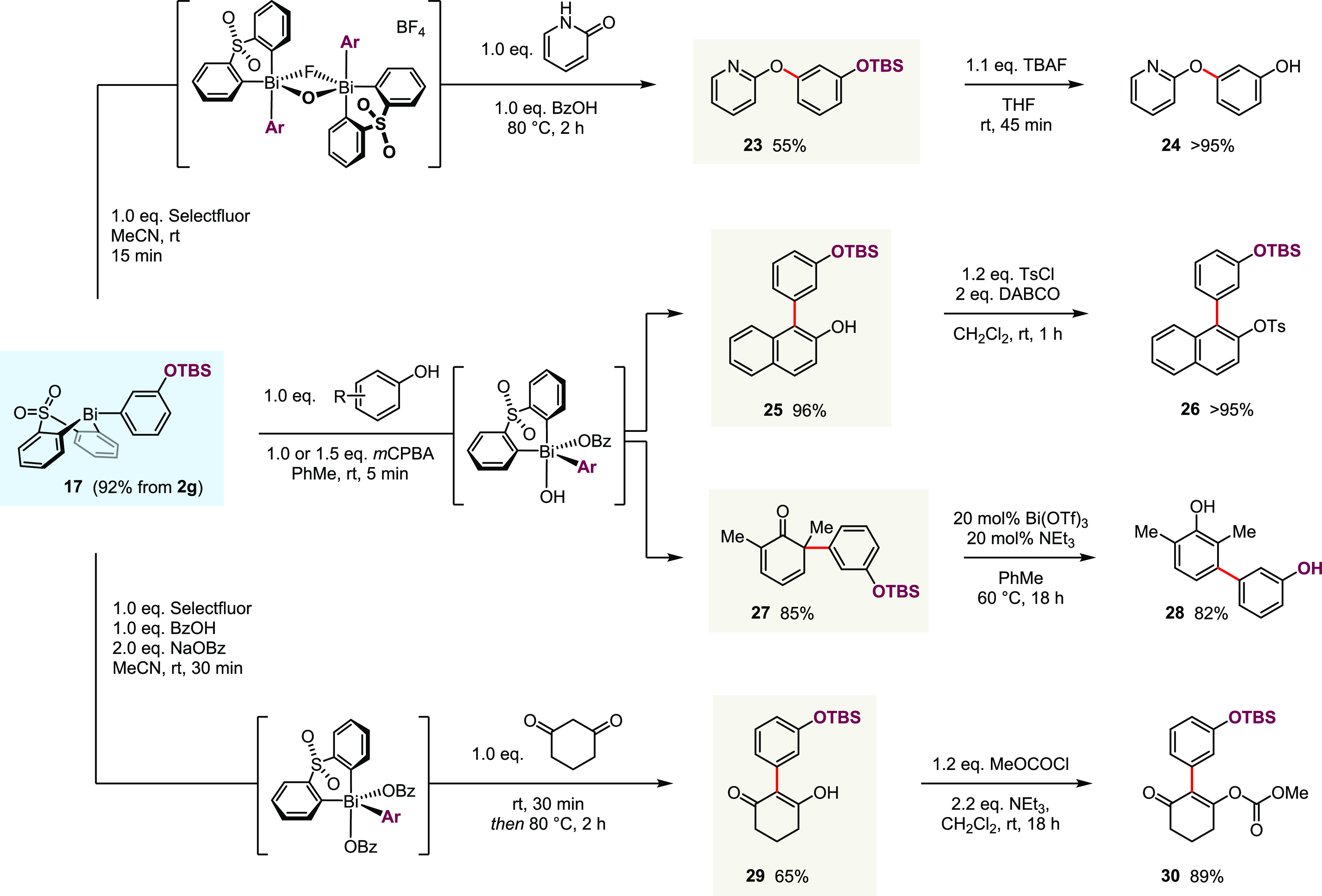
Applications of Functionalized Aryl Bismacycles to Electrophilic Arylation

To showcase the broader utility of post-transmetallation functionalization in synthesis, we applied TBS-protected hydroxyphenyl bismacycle **17** to the electrophilic arylations developed previously in our laboratory (Scheme 7).^38,41−43^ Thus, the products from *O*-selective arylation of pyridones (**23**), *ortho*-selective arylation of naphthols (**25**), *meta*-selective arylation of phenols (**28**), and α-selective arylation of cyclic 1,3-diketones (**29**) were obtained in good yields. Due to the silyl protecting group in **17**, the arylations, and selected subsequent manipulations (**25** → **26**, **29** → **30**), all proceeded without the chemoselectivity issues that would accompany the direct use of parent hydroxyphenyl bismacycle **2g**.

## Conclusions

In this publication, we have explored the compatibility of aryl bismacycles with some of the most widely used functional group interconversions in discovery chemistry. The ability to modify and add functionality to the bismacycles provides access to complex electrophilic arylating agents and overcomes potential issues with previously established synthetic routes, namely, (1) the availability of boronic acids or (2) low transmetallation rates. We anticipate that the present study will prove a valuable addition to the growing number of tools available to organobismuth chemistry and that it will expedite the adoption of bismuth-mediated arylation in both industrial and academic target-oriented synthesis.

## Data Availability

The data underlying this study are available in the published article and its Supporting Information.

## Supporting Information Available

Experimental procedures; spectra; and optimization data (PDF)

## References

[ref1] Merritt E. A.; Olofsson B. Diaryliodonium Salts: A Journey from Obscurity to Fame. Angew. Chem., Int. Ed. 2009, 48, 9052–9070. 10.1002/anie.200904689. 19876992

[ref2] Pinhey J. T. Organolead(IV) Triacetates in Organic Synthesis. Pure Appl. Chem. 1996, 68, 819–824. 10.1351/pac199668040819.

[ref3] McCormack P. J.; Guiry P. J. Category l (Organometallics), Volume 5 (Group 14 (Ge, Sn, P)), Product Class 3 (Lead Compounds), Product Sub-Classes 6-13. In Science of Synthesis; Thomas E. J.; Moloney M. G., Eds.; Houben-Weyl Thieme, 2003; pp 653–712.

[ref4] Chemistry of Hypervalent Compounds; Akiba K., Ed.; Wiley-VCH: New York, 1999.

[ref5] Ligand Coupling Reactions with Heteroatomic Compounds; Finet J.-P., Ed.; Elsevier Science: Oxford, 1998.

[ref6] Abramovitch R. A.; Barton D. H. R.; Finet J.-P. Newer Methods of Arylation. Tetrahedron 1988, 44, 3039–3071. 10.1016/S0040-4020(01)85938-X.

[ref7] Elliott G. I.; Konopelski J. P. Arylation with Organolead and Organobismuth Reagents. Tetrahedron 2001, 57, 5683–5705. 10.1016/S0040-4020(01)00385-4.

[ref8] Roughley S. D.; M Jordan A. The Medicinal Chemist′s Toolbox: An Analysis of Reactions Used in the Pursuit of Drug Candidates. J. Med. Chem. 2011, 54, 3451–3479. 10.1021/jm200187y. 21504168

[ref9] Schneider N.; M Lowe D.; A Sayle R.; A Tarselli M.; A Landrum G. Big Data from Pharmaceutical Patents: A Computational Analysis of Medicinal Chemists′ Bread and Butter. J. Med. Chem. 2016, 59, 4385–4402. 10.1021/acs.jmedchem.6b00153. 27028220

[ref10] Carey J. S.; Laffan D.; Thomson C.; Williams M. T. Analysis of the Reactions Used for the Preparation of Drug Candidate Molecules. Org. Biomol. Chem. 2006, 4, 2337–2347. 10.1039/B602413K. 16763676

[ref11] Barton D. H. R.; Lester D. J.; Motherwell W. B.; Papoula M. T. B. Obsevations on the Cleavage of the Bismuth–Carbon Bond in Bi Compounds: A New Arylation Reaction. J. Chem. Soc., Chem. Commun. 1980, 5, 246–247. 10.1039/C39800000246.

[ref12] Barton D. H. R.; Blazejewski J.-C.; Charpiot B.; Lester D. J.; Motherwell W. B.; Papoula M. T. B. Comparative Arylation Reactions with Pentaphenylbismuth and with Triphenylbismuth Carbonate. J. Chem. Soc., Chem. Commun. 1980, 17, 827–829. 10.1039/c39800000827.

[ref13] Barton D. H. R.; Bhatnagar N. Y.; Blazejewski J.-C.; Charpiot B.; Finet J.-P.; Lester D. J.; Motherwell W. B.; Papoula M. T. B.; Stanforth S. P. Pentavalent Organobismuth Reagents. Part 2. The Phenylation of Phenols. J. Chem. Soc., Perkin Trans. 1 1985, 2657–2665. 10.1039/p19850002657.

[ref14] Barton D. H. R.; Papoula M. T. B.; Guilhem J.; Motherwell W. B.; Pascard C.; Dau E. T. H. Synthesis and X-Ray Crystal Structures of Some Hindered Polyphenylated Ketones. J. Chem. Soc., Chem. Commun. 1982, 13, 732–734. 10.1039/C39820000732.

[ref15] Barton D. H. R.; Blazejewski J.-C.; Charpiot B.; Finet J.-P.; Motherwell W. B.; Papoula M. T. B.; Stanforth S. P. Pentavalent Organobismuth Reagents. Part 3. Phenylation of Enols and of Enolate and Other Anions. J. Chem. Soc., Perkin Trans. 1 1985, 2667–2675. 10.1039/P19850002667.

[ref16] Barton D. H. R.; Bhatnagar N. Y.; Finet J.-P.; Motherwell W. B. Pentavalent Organobismuth Reagents. Part vi. Comparative Migratory Aptitudes of Aryl Groups in the Arylation of Phenols and Enols by Pentavalent Bismuth Reagents. Tetrahedron 1986, 42, 3111–3122. 10.1016/S0040-4020(01)87378-6.

[ref17] Barton D. H. R.; Yadav-Bhatnagar N.; Finet J.-P.; Khamsi J.; Motherwell W. B.; Stanforth S. P. The Chemistry of Pentavalent Organobismuth Reagents: Part X. Studies on the Phenylation and Oxidation of Phenols. Tetrahedron 1987, 43, 323–332. 10.1016/S0040-4020(01)89960-9.

[ref18] Barton D. H. R.; Finet J.-P.; Giannotti C.; Halley F. The Chemistry of Pentavalent Organobismuth Reagents. Part 7. The Possible Role of Radical Mechanisms in the Phenylation Process for Bismuth(V), and Related Lead(IV), Iodine(III), and Antimony(V) Reagents. J. Chem. Soc., Perkin Trans. 1 1987, 241–249. 10.1039/P19870000241.

[ref19] Dodonov V. A.; Gushchin A. V.; Grishin D. F.; Brilkina T. G. Reactions of Some Triphenylbismuth Dialkoxides. Zhurnal Obs. Khimii 1984, 54, 100–103.

[ref20] Dodonov V. A.; Gushchin A. V.; Brilkina T. G. Preparation and Some Reactions of Triphenylbismuth Diacylates. Zhurnal Obs. Khimii 1985, 55, 73–80.

[ref21] Gagnon A.; Dansereau J.; Le Roch A. Organobismuth Reagents: Synthesis, Properties and Applications in Organic Synthesis. Synthesis 2017, 49, 1707–1745. 10.1055/s-0036-1589482.

[ref22] Gagnon A.; Benoit E.; Le Roch A. Bismuth Compounds (Update 2018). In Science of Synthesis: Knowledge Updates 2018/4; Banert K.; Clarke P. A.; Drabowicz J.; Oestreich M., Eds.; Georg Thieme Verlag: Stuttgart, 2019 10.1055/b-00000101.

[ref23] Ruffell K.; Ball L. T. Organobismuth Redox Manifolds: Versatile Tools for Synthesis. Trends Chem. 2020, 2, 867–869. 10.1016/j.trechm.2020.07.008.

[ref24] Calcatelli A.; Denton R.; Ball L. Modular Synthesis of α,α-Diaryl α-Amino Esters via Bi(V)-Mediated Arylation/S_N_2-Displacement of Kukhtin–Ramirez Intermediates. Org. Lett. 2022, 24, 8002–8007. 10.1021/acs.orglett.2c03201. 36278869 PMC9641671

[ref25] Koech P. K.; Krische M. J. Phosphine Catalyzed α-Arylation of Enones and Enals Using Hypervalent Bismuth Reagents: Regiospecific Enolate Arylation via Nucleophilic Catalysis. J. Am. Chem. Soc. 2004, 126, 5350–5351. 10.1021/ja048987i. 15113193

[ref26] Supniewski J. V.; Adams R. Organic Bismuth Compounds. I. Preparation of Tricarboxy-Triphenylbismuth Dichlorides and Certain Nitro-Triaryl Bismuth Compounds. J. Am. Chem. Soc. 1926, 48, 507–517. 10.1021/ja01413a031.

[ref27] Matano Y.; Aratani Y.; Miyamatsu T.; Kurata H.; Miyaji K.; Sasako S.; Suzuki H. Water-Soluble Non-Ionic Triarylbismuthanes. First Synthesis and Properties. J. Chem. Soc., Perkin Trans. 1 1998, 16, 2511–2518. 10.1039/A803946A.

[ref28] Murafuji T.; Nishio K.; Nagasue M.; Tanabe A.; Aono M.; Sugihara Y. Synthesis of Triarylbismuthanes Fully Substituted with Arenes, Each Bearing a π-Accepting Substituent. Synthesis 2000, 09, 1208–1210. 10.1055/s-2000-6406.

[ref29] Petiot P.; Gagnon A. Palladium-Catalyzed Cross-Coupling Reaction of Functionalized Aryl- and Heteroarylbismuthanes with 2-Halo(or 2-Triflyl)Azines and -Diazines. Eur. J. Org. Chem. 2013, 2013, 5282–5289. 10.1002/EJOC.201300850.

[ref30] Hébert M.; Petiot P.; Benoit E.; Dansereau J.; Ahmad T.; Le Roch A.; Ottenwaelder X.; Gagnon A. Synthesis of Highly Functionalized Triarylbismuthines by Functional Group Manipulation and Use in Palladium- and Copper-Catalyzed Arylation Reactions. J. Org. Chem. 2016, 81, 5401–5416. 10.1021/acs.joc.6b00767. 27231755

[ref31] Malmgren J.; Santoro S.; Jalalian N.; Himo F.; Olofsson B. Arylation with Unsymmetrical Diaryliodonium Salts: A Chemoselectivity Study. Chem. - Eur. J. 2013, 19, 10334–10342. 10.1002/chem.201300860. 23788251 PMC3884774

[ref32] Stuart D. R. Aryl Transfer Selectivity in Metal-Free Reactions of Unsymmetrical Diaryliodonium Salts. Chem. - Eur. J. 2017, 23, 15852–15863. 10.1002/chem.201702732. 28793179

[ref33] Matano Y.; Hisanaga T.; Yamada H.; Kusakabe S.; Nomura H.; Imahori H. Remarkable Substituent Effects on the Oxidizing Ability of Triarylbismuth Dichlorides in Alcohol Oxidation. J. Org. Chem. 2004, 69, 8676–8680. 10.1021/jo0485740. 15575743

[ref34] Matano Y.; Suzuki T.; Iwata T.; Shinokura T.; Imahori H. Remarkable Substituent Effects on the Oxidizing Ability of Tetraarylbismuthonium Tetrafluoroborates in Alcohol Oxidation. Bull. Chem. Soc. Jpn. 2008, 81, 1621–1628. 10.1246/bcsj.81.1621.

[ref35] Matano Y.; Miyamatsu T.; Suzuki H. Synthesis and Reaction of Unsymmetrical Tetraarylbismuthonium Salts. First Isolation of Bismuthonium Salts Bearing All Different Aryl Groups. Chem. Lett. 1998, 27, 127–128. 10.1246/cl.1998.127.

[ref36] Matano Y.; Suzuki T.; Shinokura T.; Imahori H. Mesityltriphenylbismuthonium Tetrafluoroborate as an Efficient Bismuth(V) Oxidant: Remarkable Steric Effects on Reaction Rates and Chemoselectivities in Alcohol Oxidation. Tetrahedron Lett. 2007, 48, 2885–2888. 10.1016/j.tetlet.2007.02.085.

[ref37] Louis-Goff T.; Rheingold A. L.; Hyvl J. Investigation into the Organobismuth Dismutation and Its Use for Rational Synthesis of Heteroleptic Triarylbismuthanes, Ar^1^_2_Ar^2^Bi. Organometallics 2020, 39, 778–782. 10.1021/acs.organomet.9b00777.

[ref38] Jurrat M.; Maggi L.; Lewis W.; Ball L. T. Modular Bismacycles for the Selective C–H Arylation of Phenols and Naphthols. Nat. Chem. 2020, 12, 260–269. 10.1038/s41557-020-0425-4. 32108765

[ref39] Senior A.; Ball L. T. Bismuth(V)-Mediated C–H Arylation of Phenols and Naphthols. Synlett 2021, 32, 235–240. 10.1055/s-0040-1706294.

[ref40] Suzuki H.; Murafuji T.; Azuma N. Synthesis and Reactions of Some New Heterocyclic Bismuth-(III) and -(V) Compounds. 5,10-Dihydrodibenzo[*b,e*]Bismine and Related Systems. J. Chem. Soc., Perkin Trans. 1 1992, 13, 1593–1600. 10.1039/P19920001593.

[ref41] Senior A.; Ruffell K.; Ball L. T. Meta-Selective C-H Arylation of Phenols via Regiodiversion of Electrophilic Aromatic Substitution. Nat. Chem. 2023, 15, 386–394. 10.1038/s41557-022-01101-0. 36509853

[ref42] Ruffell K.; Argent S. P.; Ling K. B.; Ball L. T. Bismuth-Mediated α-Arylation of Acidic Diketones with Ortho-Substituted Boronic Acids. Angew. Chem., Int. Ed. 2022, 61, e202210840 10.1002/anie.202210840. PMC9805042 35950691

[ref43] Ruffell K.; Gallegos L. C.; Ling K. B.; Paton R. S.; Ball L. T. Umpolung Synthesis of Pyridyl Ethers by Bi^V^-Mediated O-Arylation of Pyridones. Angew. Chem., Int. Ed. 2022, 61, e202212873 10.1002/anie.202212873. PMC10099949 36251336

[ref44] Planas O.; Wang F.; Leutzsch M.; Cornella J. Fluorination of Arylboronic Esters Enabled by Bismuth Redox Catalysis. Science 2020, 367, 313–317. 10.1126/science.aaz2258. 31949081

[ref45] Planas O.; Peciukenas V.; Cornella J. Bismuth-Catalyzed Oxidative Coupling of Arylboronic Acids with Triflate and Nonaflate Salts. J. Am. Chem. Soc. 2020, 142, 11382–11387. 10.1021/jacs.0c05343. 32536157 PMC7315642

[ref46] Magre M.; Cornella J. Redox-Neutral Organometallic Elementary Steps at Bismuth: Catalytic Synthesis of Aryl Sulfonyl Fluorides. J. Am. Chem. Soc. 2021, 143, 21497–21502. 10.1021/jacs.1c11463. 34914387 PMC8719321

[ref47] Emsley J. The Elements, 3rd ed.; Clarendon Press: Oxford, 1998.

[ref48] Bruno N. C.; Tudge M. T.; Buchwald S. L. Design and Preparation of New Palladium Precatalysts for C–C and C–N Cross-Coupling Reactions. Chem. Sci. 2013, 4, 916–920. 10.1039/C2SC20903A. 23667737 PMC3647481

[ref49] Gabriel C. M.; Lee N. R.; Bigorne F.; Klumphu P.; Parmentier M.; Gallou F.; Lipshutz B. H. Effects of Co-Solvents on Reactions Run under Micellar Catalysis Conditions. Org. Lett. 2017, 19, 194–197. 10.1021/acs.orglett.6b03468. 27997200

[ref50] Takale B. S.; Thakore R. R.; Irvine N. M.; Schuitman A. D.; Li X.; Lipshutz B. H. Sustainable and Cost-Effective Suzuki–Miyaura Couplings toward the Key Biaryl Subunits of Arylex and Rinskor Active. Org. Lett. 2020, 22, 4823–4827. 10.1021/acs.orglett.0c01625. 32521158

[ref51] Parmentier M.; Wagner M.; Wickendick R.; Baenziger M.; Langlois A.; Gallou F. A General Kilogram Scale Protocol for Suzuki–Miyaura Cross-Coupling in Water with TPGS-750-M Surfactant. Org. Process Res. Dev. 2020, 24, 1536–1542. 10.1021/acs.oprd.0c00281.

[ref52] Condon S.; Pichon C.; Davi M. Preparation and Synthetic Applications of Trivalent Arylbismuth Compounds as Arylating Reagents. A Review. Org. Prep. Proced. Int. 2014, 46, 89–131. 10.1080/00304948.2014.884369.

[ref53] Hundertmark T.; Littke A. F.; Buchwald S. L.; Fu G. C. Pd(PhCN)_2_Cl_2_/P(*t*-Bu)_3_: A Versatile Catalyst for Sonogashira Reactions of Aryl Bromides at Room Temperature. Org. Lett. 2000, 2, 1729–1731. 10.1021/ol0058947. 10880212

[ref54] Altman R. A.; Anderson K. W.; Buchwald S. L. Pyrrole-2-Carboxylic Acid as a Ligand for the Cu-Catalyzed Reactions of Primary Anilines with Aryl Halides. J. Org. Chem. 2008, 73, 5167–5169. 10.1021/jo8008676. 18543973 PMC2582381

[ref55] Barroso S.; Joksch M.; Puylaert P.; Tin S.; Bell S. J.; Donnellan L.; Duguid S.; Muir C.; Zhao P.; Farina V.; Tran D. N.; de Vries J. G. Improvement in the Palladium-Catalyzed Miyaura Borylation Reaction by Optimization of the Base: Scope and Mechanistic Study. J. Org. Chem. 2021, 86, 103–109. 10.1021/acs.joc.0c01758. 33245661

[ref56] Ishiyama T.; Murata M.; Miyaura N. Palladium(0)-Catalyzed Cross-Coupling Reaction of Alkoxydiboron with Haloarenes: A Direct Procedure for Arylboronic Esters. J. Org. Chem. 1995, 60, 7508–7510. 10.1021/jo00128a024.

[ref57] Molander G. A.; Trice S. L. J.; Kennedy S. M.; Dreher S. D.; Tudge M. T. Scope of the Palladium-Catalyzed Aryl Borylation Utilizing Bis-Boronic Acid. J. Am. Chem. Soc. 2012, 134, 11667–11673. 10.1021/ja303181m. 22769742 PMC3407959

[ref58] Caires C. C.; Guccione S. Synthesis, Structure, and Reactivity of Borate Ester Coordinated Organobismuth Compounds. Organometallics 2008, 27, 747–752. 10.1021/om7009792.

[ref59] Gilman H.; Yablunky H. L.; Svigoon A. C. Relative Reactivities of Organometallic Compounds. XXVI.* Interconversion of Bismuth and Alkali Metals. J. Am. Chem. Soc. 1939, 61, 1170–1172. 10.1021/ja01874a047.

[ref60] Gilman H.; Yablunky H. L. Unsymmetrical Organobismuth Compounds. J. Am. Chem. Soc. 1941, 63, 207–211. 10.1021/ja01846a048.

[ref61] Wittig G.; Maercker A. Nukleophile Verdrängung von Phenylgruppen in Phosphinen, Arsinen, Stibinen Und Bismutinen Durch Aryllithium-Verbindungen. J. Organomet. Chem. 1967, 8, 491–494. 10.1016/S0022-328X(00)83670-0.

[ref62] Chatterjee S.; Paine T. K. Oxygenation of Organoboronic Acids by a Nonheme Iron(II) Complex: Mimicking Boronic Acid Monooxygenase Activity. Inorg. Chem. 2015, 54, 9727–9732. 10.1021/acs.inorgchem.5b01198. 26430780

[ref63] Brandes B. D.; Jacobsen E. N. Highly Enantioselective, Catalytic Epoxidation of Trisubstituted Olefins. J. Org. Chem. 1994, 59, 4378–4380. 10.1021/jo00095a009.

[ref64] Wang Y.; Cao X.; Zhao L.; Pi C.; Ji J.; Cui X.; Wu Y. Generalized Chemoselective Transfer Hydrogenation/Hydrodeuteration. Adv. Synth. Catal. 2020, 362, 4119–4129. 10.1002/adsc.202000759.

[ref65] Popov Y. V.; Mokhov V. M.; Nebykov D. N. Colloid and Nanodimensional Catalysts in Organic Synthesis: I. Investigation of Hydrogenation Selectivity of Unsaturated Compounds with Hydrazine Hydrate and Aluminum Hydride. Russ. J. Gen. Chem. 2014, 84, 444–448. 10.1134/S1070363214030062.

[ref66] Hansch C.; Leo A.; Taft R. W. A Survey of Hammett Substituent Constants and Resonance and Field Parameters. Chem. Rev. 1991, 91, 165–195. 10.1021/cr00002a004.

[ref67] Fedorov A. Y.; Finet J.-P.; Ganina O. G.; Naumov M. I.; Shavyrin A. S. Reductive Coupling of Polyfunctionalized Organobismuth and Organolead Arylating Reagents in the Synthesis of Benzopyran Derivatives. Russ. Chem. Bull. 2005, 54, 2602–2611. 10.1007/s11172-006-0163-9.

[ref68] Finet J.-P.; Fedorov A. Y. Tris(Polymethoxyphenyl)Bismuth Derivatives: Synthesis and Reactivity. J. Organomet. Chem. 2006, 691, 2386–2393. 10.1016/j.jorganchem.2006.01.022.

