# Supplementary material for: Synthesis of Highly Functionalized Bismacycles via Post-Transmetallation Modification of Arylboronic Acids
Source: J Org Chem. 2023 Jul 12;88(14):9730–6. doi: 10.1021/acs.joc.3c00361 (PMC10367077; doi:10.1021/acs.joc.3c00361)

Experimental Section

Synthesis of Highly Functionalized Bismacycles via Post-Transmetallation Modification of Arylboronic Acids

a

Sudheesh T. Sivanandan,† Benjamin Owen,‡ Patrick J. Guiry,‡ and Liam T. Ball†,*

†School of Chemistry, University of Nottingham, Nottingham NG7 2RD, U.K. ‡Centre for Synthesis and Chemical Biology, School of Chemistry, University College Dublin, Belfield, Dublin 4, Ireland.

corresponding author: liam.ball@nottingham.ac.uk

CONTENTS

1. General Information 2
2. GP1: Preparation of Aryl Bismacycles (Scheme 2) 4
3. Cross-Couplings of Bismacycle Arylhalides (Scheme 3) 9
4. Functionalizations of Styrenyl Bismacycle **2d** (Scheme 4) 14
5. Electrophilic Substitutions (Scheme 5) 17
6. Functional Group Interconversions of Aryl Esters (Scheme 6) 25
7. Applications of Arylbismacycles to Electrophilic Arylation (Scheme 7) 32
8. Attempted Functionalizations with Reactive Organometallic Reagents 40
9. Attempted Functionalizations *via* Photoredox Catalysis 43
10. Summary of Reaction Conditions Employed 45
11. References 46
12. NMR Spectra 47

1. General Information

Procedures employing oxygen- and/or moisture-sensitive materials were performed with anhydrous solvents (*vide infra*) using standard inert-atmosphere techniques (atmosphere of anhydrous dinitrogen). MeCN, THF, toluene and Et_2O were dried using an Inert PureSolv Grubbs-type system (alumina columns, argon atmosphere). Dichloromethane was distilled from CaH_2 under an atmosphere of anhydrous dinitrogen; DMF was distilled from 4Å molecular sieves under an atmosphere of anhydrous dinitrogen. Unless stated otherwise, all reagents were used as received from commercial sources. The arylboronic acids used in this study were purchased from Fluorochem, Acros Organics, Sigma Aldrich or Alfa Aesar.

Analytical thin-layer chromatography was performed on precoated aluminium-backed plates (Silica Gel 60 F254; Merck), and visualized using a combination of UV light (254 nm), aqueous basic potassium permanganate or iodine stains. Automated flash column chromatography was performed on disposable columns pre-packed with 50 μm spherical silica gel using a Büchi C-850 equipped with a UV-vis DAD (200-800 nm) and an ELSD detector.

NMR spectra were recorded at 25 °C on a Bruker Avance 500 or 400 spectrometer (^{1}H, 500 / 400 MHz; ^{13}C{^{1}H}, 126 / 101 MHz; ^{19}F NMR, 471 / 376 MHz). Chemical shifts are reported in ppm; coupling constants, *J*, are reported in Hz and are uncorrected for digitization. The following abbreviations (and their combinations) are used to label the multiplicities: s (singlet), d (doublet), t (triplet), q (quartet), quint (quintet), sept (septet), m (multiplet), br (broad), and app (apparent). ^{1}H and ^{13}C{^{1}H} chemical shifts are reported relative to tetramethylsilane, and are referenced to the appropriate residual solvent peaks:

- $CDCl_3$: δ_H = 7.26 ppm, δ_C = 77.16 ppm
- DMSO-d_6: δ_H = 2.50 ppm, δ_C = 39.52 ppm
- acetone-d_6: δ_H = 2.05 ppm, δ_C = 29.92, 206.68 ppm

^{19}F chemical shifts are reported relative to BF_3•OEt_2. Structural assignments were made with additional information from gCOSY, gHSQC, and gHMBC experiments.

Infrared spectra of neat compounds were recorded over the range 4000-600 cm^{-1} using a Bruker Alpha FTIR spectrometer fitted with a Bruker Platinum ATR Quicksnap™ diamond cell. Melting points were measured using Stuart SMP10 or Gallenkamp melting point apparatus in open capillaries. High resolution electrospray ionization mass spectra (HRMS) were recorded using a Bruker ESITOF MicroTOF II spectrometer.

Bismacycle tosylate **1-OTs** was prepared according to literature procedure.[1]

Reactions were heated using preheated aluminium blocks placed on stirrer hot plates (Heidolph MR Hei-Tec) fitted with Pt1000 temperature probes. Unless stated otherwise, all reaction temperatures refer to the temperature of the heating block.

2. GP1: Preparation of Aryl Bismacycles (Scheme 2)

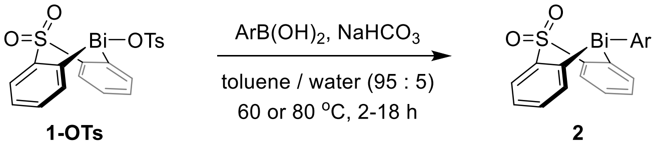

A suspension of bismacycle tosylate **1-OTs** (1.0 eq.), aryl boronic acid (1.1 eq.) and sodium bicarbonate (1.0 eq.) in toluene (0.1 M) and water (5 vol%) was heated at 60 or 80 °C for 2-18 h, as specified for individual entries. Upon completion of the reaction (as determined by ^{1}H NMR spectroscopy), the mixture was cooled to room temperature, diluted with ethyl acetate (1 vol.) and washed with 2 M aqueous NaOH (3 × 1 vol.). The organic portion was dried over $MgSO_4$, filtered and concentrated *in vacuo* to afford the aryl bismacycle as a solid.

10-(4-Bromophenyl)-10H-dibenzo[b,e][1,4]thiabismine 5,5-dioxide (**2a**)

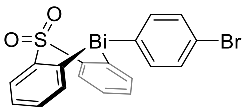

Using 5,5-dioxido-10*H*-dibenzo[*b,e*][1,4]thiabismin-10-yl 4-methylbenzenesulfonate **1-OTs** (596 mg, 1.0 mmol, 1.0 eq.) and 4-bromophenyl boronic acid (220 mg, 1.1 mmol, 1.1 eq.) in *GP1* at 60 °C for 3 h afford the title compound as a colorless solid (560 mg, 97%). Characterization data were consistent with literature values.[2]

^{1}H NMR (400 MHz, $CDCl_3$): δ 8.38 (dd, J = 7.6, 1.6 Hz, 2H), 7.84 (dd, J = 7.6, 1.6 Hz, 2H), 7.62 (d, J = 8.2 Hz, 2H), 7.51 (d, J = 8.2 Hz, 2H), 7.41 (app td, J = 7.6, 1.6 Hz, 2H), 7.36 (app td, J = 7.6, 1.6 Hz, 2H).

10-(4-Iodophenyl)-10H-dibenzo[b,e][1,4]thiabismine 5,5-dioxide (**2b**)

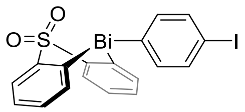

Using 5,5-dioxido-10*H*-dibenzo[*b,e*][1,4]thiabismin-10-yl 4-methylbenzenesulfonate **1-OTs** (596 mg, 1.0 mmol, 1.0 eq.) and 4-iodophenyl boronic acid (273 mg, 1.1 mmol, 1.1 eq.) in *GP1* at 60 °C for 3 h afford the title compound as a colorless solid (597 mg, 95%). Characterization data were consistent with literature values.[1]

^{1}H NMR (400 MHz, CDCl$_3$): δ 8.38 (dd, *J* = 7.6, 1.6 Hz, 2H), 7.85 (dd, *J* = 7.6, 1.6 Hz, 2H), 7.73 (d, *J* = 8.0 Hz, 2H), 7.49 (d, *J* = 8.0 Hz, 2H), 7.41 (app td, *J* = 7.6, 1.6 Hz, 2H), 7.36 (app td, *J* = 7.6, 1.6 Hz, 2H).

10-(3-Bromophenyl)-10H-dibenzo[b,e][1,4]thiabismine 5,5-dioxide (**2c**)

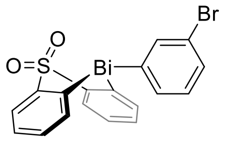

Using 5,5-dioxido-10*H*-dibenzo[*b,e*][1,4]thiabismin-10-yl 4-methylbenzenesulfonate **1-OTs** (596 mg, 1.0 mmol, 1.0 eq.) and 3-bromophenyl boronic acid (220 mg, 1.1 mmol, 1.1 eq.) in *GP1* at 60 °C for 3 h afford the title compound as a colorless solid (552 mg, 95%). Characterization data were consistent with literature values.[1]

^{1}H NMR (400 MHz, CDCl$_3$): δ 8.39 (dd, *J* = 7.5, 1.7 Hz, 2H), 7.93 (dd, *J* = 2.1, 1.1 Hz, 1H), 7.87 (dd, *J* = 7.5, 1.7 Hz, 2H), 7.65 (app dt, *J* = 7.6, 1.1 Hz, 1H), 7.48 (ddd, *J* = 7.6, 2.1, 1.1 Hz, 1H), 7.42 (app td, *J* = 7.5, 1.7 Hz, 2H), 7.38 (app td, *J* = 7.5, 1.7 Hz, 2H), 7.30 (app t, *J* = 7.6 Hz, 1H).

10-(4-Vinylphenyl)-10H-dibenzo[b,e][1,4]thiabismine 5,5-dioxide (**2d**)

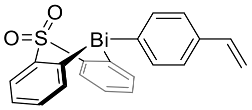

Using 5,5-dioxido-10*H*-dibenzo[*b,e*][1,4]thiabismin-10-yl 4-methylbenzenesulfonate **1-OTs** (596 mg, 1.0 mmol, 1.0 eq.) and 4-vinylphenyl boronic acid (163 mg, 1.1 mmol, 1.1 eq.) in *GP1* at 60 °C for 3 h afford the title compound as a colorless solid (486 mg, 92%). Characterization data were consistent with literature values.[1]

^{1}H NMR (400 MHz, $CDCl_3$): δ 8.38 (dd, J = 7.7, 1.5 Hz, 2H), 7.87 (dd, J = 7.7, 1.5 Hz, 2H), 7.72 (d, J = 8.0 Hz, 2H), 7.44 (d, J = 8.0 Hz, 2H), 7.40 (app td, J = 7.7, 1.5 Hz, 2H), 7.34 (app td, J = 7.7, 1.5 Hz, 2H), 6.69 (dd, J = 17.7, 10.9 Hz, 1H), 5.77 (dd, J = 17.7, 0.9 Hz, 1H), 5.27 (dd, J = 10.9, 0.9 Hz, 1H).

N-(3-(5,5-Dioxido-10H-dibenzo[b,e][1,4]thiabismin-10-yl)phenyl)acetamide (**2e**)

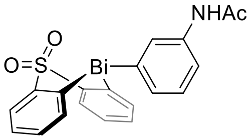

Using 5,5-dioxido-10*H*-dibenzo[*b,e*][1,4]thiabismin-10-yl 4-methylbenzenesulfonate **1-OTs** (596 mg, 1.0 mmol, 1.0 eq.) and 3-acetamidophenyl boronic acid (197 mg, 1.1 mmol, 1.1 eq.) in *GP1* at 60 °C for 18 h afford the title compound as a colorless solid (475 mg, 85%). Characterization data were consistent with literature values.[3]

^{1}H NMR (400 MHz, DMSO): δ 9.77 (s, 1H), 8.26-8.18 (m, 2H), 7.99-7.94 (m, 2H), 7.87 (dd, J = 2.2, 1.2 Hz, 1H), 7.67 (ddd, J = 8.0, 2.2, 1.2 Hz, 1H), 7.48-7.43 (m, 5H), 7.35 (app t, J = 8.0 Hz, 1H), 1.95 (s, 3H).

10-(4-Hydroxyphenyl)-10H-dibenzo[b,e][1,4]thiabismine 5,5-dioxide (**2f**)

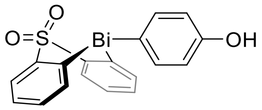

Using 5,5-dioxido-10*H*-dibenzo[*b,e*][1,4]thiabismin-10-yl 4-methylbenzenesulfonate **1-OTs** (596 mg, 1.0 mmol, 1.0 eq.) and 4-hydroxyphenyl boronic acid (152 mg, 1.1 mmol, 1.1 eq.) in *GP1* at 60 °C for 3 h afford the title compound as a colorless solid (430 mg, 83%).

mp: 174-177 °C;

ν_{max} **(ATR, cm^{-1}):** 3439 (br), 2918 (w), 1577 (s), 1489 (m), 1252 (m), 1151 (s), 816 (s), 564 (s);

^{1}H NMR (400 MHz, DMSO-*d*$_6$): δ 9.40 (s, 1H), 8.24-8.16 (m, 2H), 7.99-7.88 (m, 2H), 7.53 (d, *J* = 8.3 Hz, 2H), 7.48-7.37 (m, 4H), 6.77 (d, *J* = 8.3 Hz, 2H);

^{13}C{^{1}H} NMR (101 MHz, DMSO-*d*$_6$): δ 163.3, 158.0, 157.6, 142.4, 140.2, 138.8, 133.5, 128.3, 126.1, 118.3;

HRMS (ESI): *m/z* [M + H]$^+$ calcd for $C_{18}H_{14}BiO_3S$, 519.0462; found, 519.0460.

10-(3-Hydroxyphenyl)-10H-dibenzo[b,e][1,4]thiabismine 5,5-dioxide (**2g**)

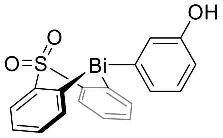

Using 5,5-dioxido-10*H*-dibenzo[*b,e*][1,4]thiabismin-10-yl 4-methylbenzenesulfonate **1-OTs** (596 mg, 1.0 mmol, 1.0 eq.) and 4-hydroxyphenyl boronic acid (152 mg, 1.1 mmol, 1.1 eq.) in *GP1* at 60 °C for 3 h afford the title compound as a colorless solid (497 mg, 96%).

mp: 195-198 °C;

ν_{max} **(ATR, cm^{-1}):** 3385 (br w), 3037 (w), 1573 (m), 1468 (m), 1250 (m), 1145 (s), 738 (s), 564 (s);

^{1}H NMR (400 MHz, CDCl$_3$): δ 8.40 (dd, *J* = 7.4, 1.6 Hz, 2H), 7.91 (dd, *J* = 7.4, 1.6 Hz, 2H), 7.48-7.33 (m, 6H), 7.26 (d, *J* = 2.8 Hz, 1H), 6.85 (app. dt, *J* = 6.0, 2.8 Hz, 1H), 4.80 (s, 1H);

$^{13}C\{^{1}H\}$ NMR (101 MHz, DMSO-d_6): δ 169.3, 163.5, 159.7, 141.9, 138.3, 133.1, 131.1, 128.4, 127.9, 125.8, 125.1, 115.0;

HRMS (ESI): *m/z* $[M + H]^+$ calcd for $C_{18}H_{14}BiO_3S$, 519.0462; found, 519.0458.

Methyl 3-(5,5-dioxido-10H-dibenzo[b,e][1,4]thiabismin-10-yl)benzoate (**2h**)

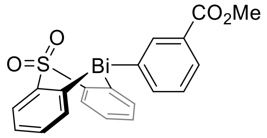

Using 5,5-dioxido-10*H*-dibenzo[*b,e*][1,4]thiabismin-10-yl 4-methylbenzenesulfonate **1-OTs** (1.79 g, 3.0 mmol, 1.0 eq.) and (3-(methoxycarbonyl)phenyl)boronic acid (594 mg, 3.3 mmol, 1.1 eq.) in *GP1* at 80 °C for 18 h afford the title compound as a colorless solid (1.62 g, 96%).

mp:158-163 °C;

ν_{max} (ATR, cm^{-1}): 3053 (w), 2950 (w), 1719 (s), 1562 (m), 1436 (m), 1285 (s), 1151 (s), 1117 (m), 765 (s), 589 (s);

^{1}H NMR (400 MHz, $CDCl_3$): δ 8.56 (app. t, J = 1.5 Hz, 1H), 8.40 (dd, J = 7.6, 1.4 Hz, 2H), 8.03 (app. dt, J = 7.8, 1.5 Hz, 1H), 7.87-7.81 (m, 3H), 7.46-7.39 (m, 3H), 7.36 (app. td, J = 7.6, 1.4 Hz, 2H), 3.90 (s, 3H);

$^{13}C\{^{1}H\}$ NMR (101 MHz, $CDCl_3$): δ 167.4, 165.7, 158.9, 143.8, 141.80, 139.2, 137.6, 133.7, 131.9, 131.3, 129.9, 128.5, 127.4, 52.4;

HRMS (ESI): *m/z* $[M + Na]^+$ calcd for $C_{20}H_{15}BiNaO_4S$, 583.0387; found, 583.0374.

3. Cross-Couplings of Bismacycle Arylhalides (Scheme 3)

10-(4'-Fluoro-[1,1'-biphenyl]-4-yl)-10H-dibenzo[b,e][1,4]thiabismine 5,5-dioxide (**3**)

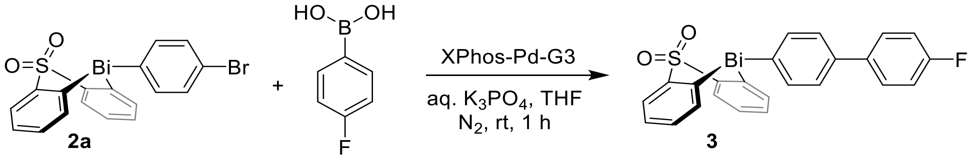

A Schlenk tube equipped with a magnetic stir bar was charged with Pd-XPhos-G3 (6.3 mg, 0.0075 mmol, 5 mol%), bromophenyl bismacycle **2a** (87.0 mg, 0.15 mmol, 1.0 eq.), and 4-fluorophenyl boronic acid (31.5 mg, 0.225 mmol, 1.5 eq.). The tube was then evacuated and backfilled with anhydrous dinitrogen three times. Degassed THF (1.0 mL) was added, followed by degassed 0.5 M aqueous K_3PO_4 solution (2 mL). The reaction was then stirred at rt for 1 h. After completion, the reaction mixture was diluted with water (10 mL) and diethyl ether (10 mL) and the layers are separated. The aqueous layer was extracted with diethyl ether (3 × 10 mL), the combined organic portions were dried over magnesium sulfate, filtered, and concentrated *in vacuo*. Purification *via* flash column chromatography over silica gel (eluent: 5-10% EtOAc in cyclohexane) afforded the title compound as an off-white solid (64 mg, 71%).

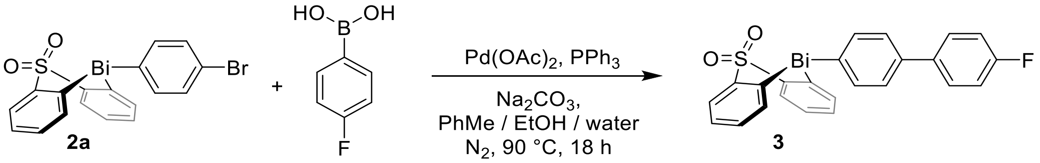

$Pd(OAc)_2$ (2.25 mg, 0.01 mmol, 5 mol%) was added against a flow of dinitrogen to a biphasic solution of bromophenyl bismacycle **2a** (116 mg, 0.2 mmol, 1.0 eq.), 4-fluorophenylboronic acid (30.8 mg, 0.22 mmol, 1.1 eq.), PPh_3 (7.87 mg, 0.03 mmol, 15 mol%) and Na_2CO_3 (42.4 mg, 0.4 mmol, 2 eq.) in degassed toluene (2 mL), EtOH (1 mL) and water (2 mL). The reaction mixture was heated at 90 °C for 18 h and then cooled to rt. The mixture was filtered through celite, diluted with EtOAc (30 mL) and washed with aq. NaOH (2 M; 3 × 10 mL). The organic portion was dried over magnesium sulfate, filtered, and concentrated *in vacuo*. Purification *via* flash column chromatography over silica gel (eluent: 5-10% EtOAc in cyclohexane) afforded the title compound as an off-white solid (59.6 mg, 50%).

mp: 178-181 °C;

ν_{max} **(ATR, cm^{-1}):** 3036 (w), 2921 (w), 2851 (w), 1514 (m), 1302 (s), 1151 (s), 806 (s), 564 (s);

^{1}H NMR (400 MHz, $CDCl_3$): δ 8.40 (dd, *J* = 7.6, 1.5 Hz, 2H), 7.91 (dd, *J* = 7.6, 1.5 Hz, 2H), 7.82 (d, *J* = 8.0 Hz, 2H), 7.58 (d, *J* = 8.0 Hz, 2H), 7.54 (dd, *J* = 8.7, 5.4 Hz, 2H), 7.42 (app. td, *J* = 7.6, 1.5 Hz, 2H), 7.36 (app. td, *J* = 7.6, 1.5 Hz, 2H), 7.13 (app. t, *J* = 8.7 Hz, 2H);

^{13}C{^{1}H} NMR (101 MHz, $CDCl_3$): δ 164.7, 162.8 (d, *J* = 247.0 Hz), 158.6, 141.9, 140.5, 139.4, 137.7, 137.0 (d, *J* = 3.3 Hz), 133.6, 129.4, 128.8 (d, *J* = 8.1 Hz), 128.4, 127.3, 115.9 (d, *J* = 21.2 Hz);

^{19}F NMR (376 MHz, $CDCl_3$): δ -115.10 (tt, *J* = 8.6, 5.3 Hz);

HRMS (ESI): *m/z* $[M + Na]^+$ calcd for $C_{24}H_{16}BiFNaO_2S$, 619.0551; found, 619.0532.

10-(4-((Trimethylsilyl)ethynyl)phenyl)-10H-dibenzo[b,e][1,4]thiabismine 5,5-dioxide (**4**)

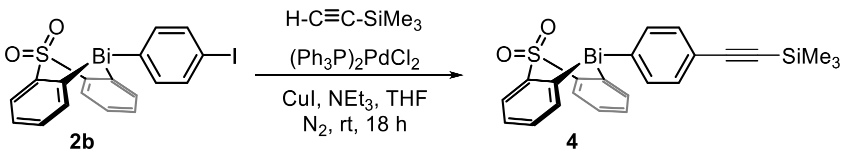

An oven-dried Schlenk tube equipped with a magnetic stir-bar was charged with $(Ph_3P)_2PdCl_2$ (10.5 mg, 0.015 mmol, 10 mol%), CuI (3.7 mg, 0.0195 mmol, 13 mol%) and iodophenyl bismacycle **2b** (94.2 mg, 0.15 mmol, 1.0 eq.). The tube was then evacuated and backfilled with anhydrous dinitrogen three times. Anhydrous THF (2.0 mL) and triethylamine (30.4 mg, 42 μL, 0.3 mmol, 2.0 eq.) were added sequentially, and the reaction was stirred at rt. Trimethylsilylacetylene (16.2 mg, 23.5 μL, 0.165 mmol, 1.1 eq.) was added to the reaction mixture, which was stirred for 18 h at rt. Once the reaction was complete (as determined by TLC), the mixture was diluted with ethyl acetate (5 mL) and filtered through Celite®. The filtrate was concentrated *in vacuo*; *p*urification *via* flash column chromatography over silica gel (eluent: 5-10% EtOAc in cyclohexane) afforded the title compound as a yellow solid (54 mg, 58%). *This compound could not be separated from ca 4% bismacycle-derived impurity*.

mp: 107-110 °C;

ν_{max} (ATR, cm^{-1}): 3047 (w), 2956 (w), 2153 (m), 1563 (m), 1431 (m), 1299 (s), 1149 (s), 842 (s), 585 (s);

^{1}H NMR (400 MHz, CDCl$_3$): δ 8.40 (dd, J = 7.7, 1.4, Hz, 2H), 7.85 (dd, J = 7.7, 1.4, Hz, 2H), 7.73 (d, J = 8.1 Hz, 2H), 7.51 (d, J = 8.1 Hz, 2H), 7.43 (app. td, J = 7.7, 1.4 Hz, 2H), 7.36 (app. td, J = 7.7, 1.4 Hz, 2H), 0.27 (s, 9H);

^{13}C{^{1}H} NMR (101 MHz, CDCl$_3$): δ 166.7, 158.9, 141.8, 138.7, 137.6, 134.1, 133.6, 128.4, 127.3, 123.4, 104.9, 95.7, 0.1;

HRMS (ESI): *m/z* [M + Na]$^+$ calcd for $C_{23}H_{21}BiNaO_2SSi$, 621.0728; found, 621.0746.

10-(3-((4-Fluorophenyl)amino)phenyl)-10H-dibenzo[b,e][1,4]thiabismine 5,5-dioxide (**5**)

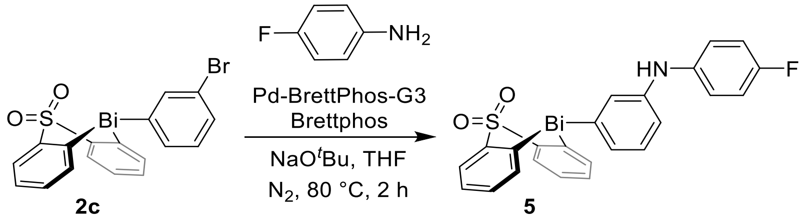

An oven-dried Schlenk tube equipped with a magnetic stir-bar was charged was charged with Pd-BrettPhos-G3 (1.4 mg, 0.0015 mmol, 1.0 mol%), NaO^{t}Bu (17.3 mg, 0.18 mmol, 1.2 eq.), and bromophenyl bismacycle **2c** (87.0 mg, 0.15 mmol, 1.0 eq.). The tube was evacuated and backfilled with anhydrous dinitrogen three times. 4-Fluoroaniline (17.0 μL, 0.18 mmol, 1.2 eq.) was added, followed by anhydrous THF (2 mL). The reaction was stirred at 80 °C until complete consumption of bromophenyl bismacycle **2c** was observed by TLC. The reaction was cooled to room temperature, diluted with ethyl acetate (5 mL), and filtered through a pad of Celite®. Purification by flash column chromatography over silica gel (eluent: 10-15% EtOAc in cyclohexane) afforded the title compound as an off-white solid (71 mg, 77%).

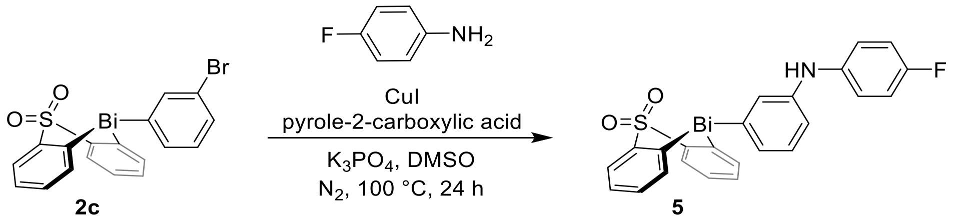

An oven-dried Schlenk tube was charged with K_3PO_4 (42.5 mg, 0.2 mmol, 2.0 eq.). The tube was flame-dried under vacuum, and cooled under n atmosphere of anhydrous dinitrogen. CuI (1.9 mg, 0.01 mmol. 10 mol%), pyrrole-2-carboxylic acid (2.2 mg, 0.02 mmol, 20 mol%), bromophenyl bismacycle **2c** (58.0 mg, 0.1 mmol, 1.0 eq.), and a magnetic stir bar were added to the cooled vessel. The tube was then evacuated and backfilled with anhydrous dinitrogen three times. Anhydrous DMSO (1.0 mL) and 4-fluoroaniline (19 μL, 22.2 mg, 0.2 mmol, 2.0 eq.) were then added by syringe. The reaction was heated at 100 °C with vigorous stirring until TLC of the crude reaction mixture indicated that the aryl halide had been completely consumed (24 h). The reaction mixture was then allowed to cool to room temperature and ethyl acetate (10 mL) and sat. aq. NH_4Cl (10 mL) were added. The organic layer was separated, and filtered through a plug of silica gel. The aqueous layer was extracted twice more with ethyl acetate (10 mL), and each extract was sequentially filtered through the pad of silica gel. The filtrate was

concentrated *in vacuo* and the resulting residue was purified by flash chromatography over triethylamine-washed silica gel (eluent: 10-15% EtOAc in cyclohexane) to afford the title compound as an off-white solid (13 mg, 28%).

mp: 215-218 °C;

ν_{max} (ATR, cm^{-1}): 3386 (m), 3041 (w), 1573 (m), 1503 (s), 1376 (m), 1278 (s), 1147 (s), 760 (s), 506 (s);

^{1}H NMR (400 MHz, $CDCl_3$): δ 8.40 (dd, *J* = 7.6, 1.5 Hz, 2H), 7.95 (dd, *J* = 7.6, 1.5 Hz, 2H), 7.43 (app. td, *J* = 7.6, 1.5 Hz, 2H), 7.41-7.35 (m, 4H), 7.31 (app. dt, *J* = 7.2, 1.1 Hz, 1H), 7.02-6.89 (m, 5H), 5.54 (s, 1H);

^{13}C{^{1}H} NMR (101 MHz, $CDCl_3$): δ 166.6, 158.8, 158.2 (d, *J* = 240.7 Hz), 146.8, 141.8, 138.6 (d, *J* = 2.0 Hz), 137.7, 133.5, 131.6, 130.4, 128.3, 127.3, 127.0, 120.7 (d, *J* = 7.8 Hz), 116.9, 116.1 (d, *J* = 22.7 Hz);

^{19}F{^{1}H} NMR (376 MHz, $CDCl_3$): δ -121.51 (tt, *J* = 8.1, 5.0 Hz);

HRMS (ESI): *m/z* $[M + H]^+$ calcd for $C_{24}H_{18}BiFNO_2S$, 612.0841; found, 612.0811.

4. Functionalizations of Styrenyl Bismacycle 2d (Scheme 4)

Methyl (E)-3-(4-(5,5-dioxido-10H-dibenzo[b,e][1,4]thiabismin-10-yl)phenyl)acrylate (**7**)

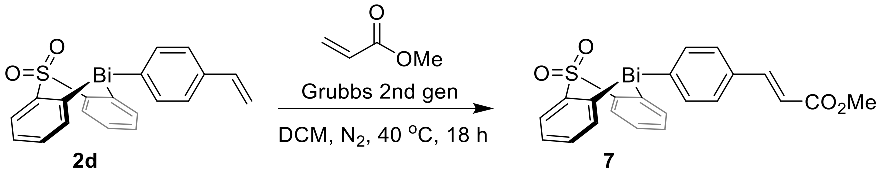

Styrenyl bismacycle **2d** (79.2 mg, 0.15 mmol, 1.0 eq.) and methyl acrylate (27 μL, 0.3 mmol, 2.0 eq.) were added to a solution of Grubbs 2nd generation catalyst (6.4 mg, 0.0075 mmol, 5.0 mol%) in CH_2Cl_2 (2 mL) in a Schlenk tube under nitrogen atmosphere. The reaction was heated to 40 °C under an atmosphere of anhydrous dinitrogen and stirred for 18 h. The reaction mixture was then reduced in volume to 0.5 mL; purification by flash column chromatography over silica gel (eluent: 15-20% EtOAc in cyclohexane) afforded the title compound as an off-white solid (75 mg, 85%).

mp: 217-219 °C;

ν_{max} (ATR, cm^{-1}): 3048 (w), 2942 (w), 2923 (w), 1707 (s), 1624 (s), 1567 (m), 1428 (m), 1288 (s), 1147 (s), 734 (s), 559 (s);

^{1}H NMR (400 MHz, $CDCl_3$): δ 8.39 (d, *J* = 7.4 Hz, 2H), 7.85 (d, *J* = 7.4 Hz, 2H), 7.79 (d, *J* = 7.6 Hz, 2H), 7.65 (d, *J* = 16.0 Hz, 1H), 7.53 (d, *J* = 7.6 Hz, 2H), 7.41 (app. t, *J* = 7.4 Hz, 2H), 7.35 (app. t, *J* = 7.4 Hz, 2H), 6.44 (d, *J* = 16.0 Hz, 1H), 3.80 (s, 3H);

^{13}C{^{1}H} NMR (101 MHz, $CDCl_3$): δ 168.9, 167.4, 158.9, 144.8, 141.8, 139.5, 137.6, 134.7, 133.7, 130.3, 128.5, 127.4, 118.5, 51.9;

HRMS (ESI): *m/z* [M + Na]$^+$ calcd for $C_{22}H_{17}BiNaO_4S$, 609.0544; found, 609.0574.

10-(4-(1,2-dihydroxyethyl)phenyl)-10H-dibenzo[b,e][1,4]thiabismine 5,5-dioxide (**8**)

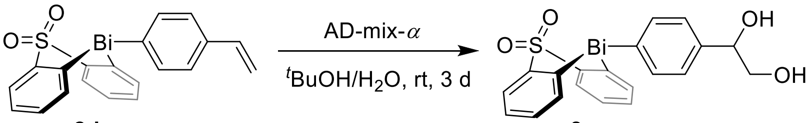

AD-mix-α (195 mg, 1.3 g/mmol) was added to a solution of styrenyl bismacycle **2d** (79.2 mg, 0.15 mmol, 1.0 eq.) in ^{t}BuOH (1.0 mL) and water (1.0 mL), and the mixture was stirred for 3 days at room temperature. After completion of the reaction (determined by TLC), the reaction mixture was quenched with saturated aqueous $Na_2S_2O_3$ solution. Brine was added, and the mixture was extracted with ethyl acetate (3 × 100 mL). The organic portions were combined, washed with brine, dried over $MgSO_4$, filtered and concentrated *in vacuo*. Purification by flash column chromatography over silica gel (eluent: 25-30% EtOAc in cyclohexane) afforded the title compound as a colorless solid (80 mg, 95%).

mp: 116-119 °C;

ν_{max} (ATR, cm^{-1}): 3334 (br m), 2921 (w), 2852 (w), 1561 (w), 1380 (m), 1282 (m), 1148 (s), 759 (s), 585 (s);

^{1}H NMR (400 MHz, $CDCl_3$): δ 8.37 (d, J = 7.7 Hz, 2H), 7.84 (d, J = 7.7 Hz, 2H), 7.75 (d, J = 8.0 Hz, 2H), 7.36-7.43 (m, 4H), 7.33 (app. t, J = 7.7 Hz, 2H), 4.87-4.72 (m, 1H), 3.83-3.73 (m, 1H), 3.71-3.56 (m, 1H), 2.72 (br s, 1H), 2.22 (br s, 1H);

^{13}C{^{1}H} NMR (101 MHz, $CDCl_3$): δ 165.3, 158.5, 141.7, 140.9, 138.9, 137.6, 133.5, 128.6, 128.3, 127.2, 74.6, 68.0;

HRMS (ESI): *m/z* [M + H]$^+$ calcd for $C_{20}H_{18}BiO_4S$, 563.0724; found, 563.0731.

10-(4-(2-Hydroxyethyl)phenyl)-10H-dibenzo[b,e][1,4]thiabismine 5,5-dioxide (**9**)

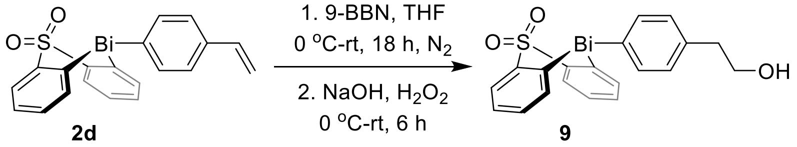

A Schlenk flask containing styrenyl bismacycle **2d** (79.2 mg, 0.15 mmol, 1.0 eq.) was evacuated and back-filled with dinitrogen three times, then anhydrous THF (2.0 mL) was added and the resulting solution was cooled to 0 °C. A solution of 9-BBN (0.5 M solution in THF; 0.90 mL, 0.45 mmol, 3.0 eq.) was added, then the mixture was allowed to warm to room temperature, and was stirred overnight. The mixture was then cooled to 0 °C before 2 M aqueous NaOH (1.0 mL) and 30% aqueous H_2O_2 (1.0 mL) were added slowly. The mixture was stirred for 6 h at room temperature, then extracted with EtOAc (20 mL); the organic portion was washed with brine, dried over $MgSO_4$, filtered and concentrated *in vacuo*. Purification by flash column chromatography over silica gel (eluent: 50-60% EtOAc in cyclohexane) afforded the title compound as a colorless oil (65 mg, 77%). *This compound could not be separated from ca 15% impurities due to stability on silica gel.*

***ν_{max}* (ATR, cm^{-1}):** 3366 (br w), 3047 (w), 2923 (m), 1562 (m), 1437 (m), 1284 (s), 1149 (s), 1087 (m), 739 (s), 585 (s);

^{1}H NMR (400 MHz, $CDCl_3$): δ 8.38 (dd, J = 7.6, 1.4 Hz, 2H), 7.87 (dd, J = 7.2, 1.4 Hz, 2H), 7.71 (d, J = 7.9 Hz, 2H), 7.40 (app. td, J = 7.5, 1.4 Hz, 2H), 7.34 (app. td, J = 7.3, 1.5 Hz, 2H), 7.28 (d, J = 7.9 Hz, 2H), 3.88 (t, J = 6.6 Hz, 2H), 2.86 (t, J = 6.6 Hz, 2H);

^{13}C{^{1}H} NMR (101 MHz, $CDCl_3$): δ 163.6, 158.5, 141.8, 139.3, 139.1, 137.7, 133.5, 131.7, 128.3, 127.2, 63.6, 39.4;

HRMS (ESI): *m/z* $[M + H]^+$ calcd for $C_{20}H_{18}BiO_3S$, 547.0775; found, 547.0774.

5. Electrophilic Substitutions (Scheme 5)

N-(3-(5,5-Dioxido-10H-dibenzo[b,e][1,4]thiabismin-10-yl)phenyl)-N-methylacetamide (**12**)

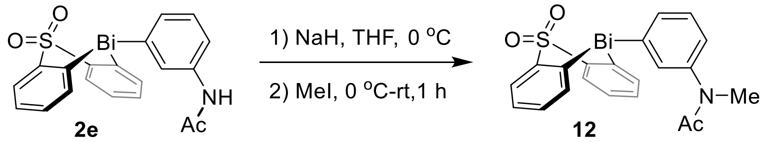

NaH (60% in mineral oil; 13.2 mg, 0.33 mol, 2.2 eq.) was added to a solution of bismacyclic anilide **2e** (83.4 mg, 0.15 mmol, 1.0 eq.) in anhydrous THF (2.0 mL) at 0 °C. The resulting mixture was stirred for 15 min at 0 °C, then iodomethane (37.4 µL, 0.60 mmol, 4.0 eq.) was added slowly and the reaction mixture was stirred at room temperature until completion (determined by TLC). The reaction was concentrated *in vacuo* before water (10 mL) was added and the mixture was extracted with ethyl acetate (3 × 20 mL). The combined organic portions were washed with brine (3 × 20 mL), dried over Na_2SO_4, filtered and concentrated *in vacuo*. Purification by flash column chromatography over silica gel (eluent: 40-50% EtOAc in cyclohexane) afforded the title compound as a colorless solid (83 mg, 97%).

mp: 98-101 °C;

ν_{max} (ATR, cm^{-1}): 3043 (br m), 2923 (w), 1636 (m), 1575 (m), 1376 (m), 1284 (s), 1148 (s), 739 (s), 586 (s);

^{1}H NMR (400 MHz, $CDCl_3$): δ 8.40 (dd, *J* = 7.7, 1.4 Hz, 2H), 7.78-7.86 (m, 3H), 7.55-7.46 (m, 2H), 7.43 (app. t, *J* = 7.7 Hz, 2H), 7.35 (app. t, *J* = 7.7 Hz, 2H), 7.18 (d, *J* = 7.6 Hz, 1H), 3.14 (s, 3H), 1.73 (s, 3H);

^{13}C{^{1}H} NMR (101 MHz, $CDCl_3$): δ 170.3, 167.3, 159.3, 147.6, 141.9, 138.0, 137.6, 137.5, 133.6, 131.7, 128.7, 127.5, 127.3, 37.3, 22.6;

HRMS (ESI): *m/z* $[M + H]^+$ calcd for $C_{21}H_{19}BiNO_3S$, 574.0884; found, 574.0873.

N-Allyl-N-(3-(5,5-dioxido-10H-dibenzo[b,e][1,4]thiabismin-10-yl)phenyl)acetamide (**13**)

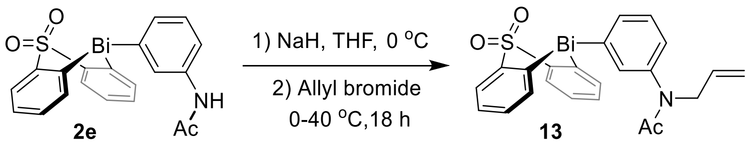

Bismacyclic anilide **2e** (83.4 mg, 0.15 mmol, 1.0 eq.) was added to a suspension of NaH (60% in mineral oil; 7.9 mg, 0.33 mmol, 2.2 eq.) in anhydrous THF (2.0 mL) at 0 °C. The mixture was stirred for 15 min at 0 °C, then allyl bromide (39.0 μL, 0.45 mol, 3.0 eq.) was then added dropwise and the reaction mixture was heated at 40 °C overnight. The reaction mixture was cooled to 0 °C and saturated aqueous NH_4Cl solution (10 mL) was added dropwise. The reaction mixture was extracted with EtOAc (2 × 20 mL). The combined organic portions were dried over $MgSO_4$, filtered and concentrated *in vacuo*. Purification by flash column chromatography over silica gel (eluent: 40-50% EtOAc in cyclohexane) afforded the title compound as a colorless solid (81 mg, 87%). *This compound could not be separated from ca 10% arene-derived impurity*.

mp: 164-167 °C;

ν_{max} (ATR, cm^{-1}): 2920 (m), 2851 (w), 1662 (s), 1562 (m), 1387 (m), 1284 (s), 1151 (s), 1086 (m), 1073 (m), 740 (s), 563 (s);

^{1}H NMR (400 MHz, $CDCl_3$): δ 8.42 (d, *J* = 7.7 Hz, 2H), 7.88 (d, *J* = 7.7 Hz, 1H), 7.83 (d, *J* = 7.7 Hz, 2H), 7.55 (t, *J* = 7.7 Hz, 1H), 7.45 (app. td, *J* = 7.7, 1.3 Hz, 2H), 7.41 (s, 1H), 7.35 (app. td, *J* = 7.7, 1.3 Hz, 2H), 7.18 (d, *J* = 7.7 Hz, 1H), 5.67 (ddt, *J* = 17.1, 10.1, 8.3 Hz, 1H), 5.02 (d, *J* = 10.1 Hz, 1H), 4.96 (d, *J* = 17.1 Hz, 1H), 4.19 (d, *J* = 6.3 Hz, 2H), 1.71 (s, 3H);

^{13}C{^{1}H} NMR (101 MHz, $CDCl_3$): δ 169.9, 167.1, 159.3, 146.1, 141.9, 139.5, 137.5, 137.4, 133.6, 133.1, 131.4, 128.6, 128.3, 127.5, 118.0, 52.0, 22.8;

HRMS (ESI): *m/z* [M + H]$^+$ calcd for $C_{23}H_{21}BiNO_3S$, 600.1041; found, 600.1031.

4-(5,5-Dioxido-10H-dibenzo[b,e][1,4]thiabismin-10-yl)phenyl (4-fluorophenyl)carbamate (**14**)

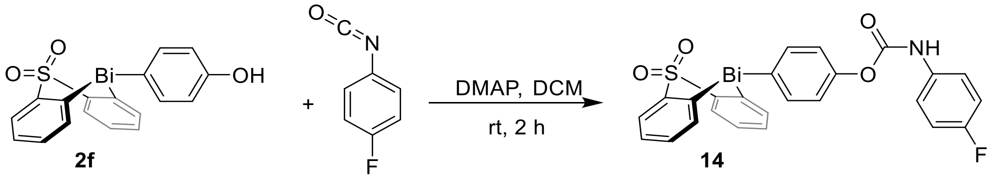

4-Fluorophenyl isocyanate (18.8 μL, 0.165 mmol, 1.1 eq.) was added dropwise to a solution of hydroxyphenyl bismacycle **2f** (77.7 mg, 0.15 mmol, 1.0 eq.) and 4-dimethylaminopyridine (3.66 mg, 0.03 mmol, 2.0 mol%) in anhydrous CH_2Cl_2 (2 mL). The reaction mixture was stirred at room temperature for 2 h, then the precipitate was collected by Büchner filtration, washed with cold CH_2Cl_2 and dried *in vacuo* to afford the title compound as a colorless solid (96 mg, 98%).

mp: 166-169 °C;

ν_{max} (ATR, cm^{-1}): 3299 (m), 2921 (w), 2851 (w), 1737 (s), 1542 (m), 1508 (m), 1283 (m), 1196 (s), 740 (s), 563 (s);

^{1}H NMR (400 MHz, DMSO-*d*$_6$): δ 10.23 (s, 1H), 8.27-8.20 (m, 2H), 8.02-7.96 (m, 2H), 7.78 (d, *J* = 8.4 Hz, 2H), 7.51 (dd, *J* = 8.8, 4.9 Hz, 2H), 7.48-7.43 (m, 4H), 7.21 (d, *J* = 8.4 Hz, 2H), 7.17 (app. t, *J* = 8.9 Hz, 2H);

^{13}C{^{1}H} NMR (101 MHz, DMSO-*d*$_6$): δ 165.5, 164.2, 158.0 (d, *J* = 238.4 Hz), 151.7, 150.0, 142.0, 139.5, 138.3, 134.9, 133.2, 127.9, 125.8, 123.9, 120.1, 115.5 (d, *J* = 22.4 Hz);

^{19}F NMR (376 MHz, acetone-*d*$_6$): δ -121.52 (br s);

HRMS (ESI): *m/z* [M + Na]$^+$ calcd for $C_{25}H_{17}BiFNNaO_4S$, 678.0559; found, 678.0550.

4-(5,5-Dioxido-10H-dibenzo[b,e][1,4]thiabismin-10-yl)phenyl 4-methylbenzenesulfonate (**15**)

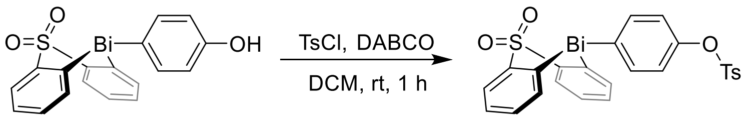

A solution of tosyl chloride (34.3 mg, 0.18 mmol, 1.2 eq.) in anhydrous CH_2Cl_2 (1.0 mL) was added dropwise to a solution of hydroxyphenyl bismacycle **2f** (77.7 mg, 0.15 mmol, 1.0 eq.) and DABCO (33.7 mg, 0.3 mmol, 2.0 eq.) in anhydrous CH_2Cl_2 (2 mL) at 0 °C. The mixture was allowed to warm to room temperature and was stirred for 1 h. The mixture was then diluted with Et_2O (20 mL), and water (10 mL) was added. The organic phase was separated and washed successively with saturated aqueous $NaHCO_3$ (2 × 25 mL) and brine (25 mL). After drying over $MgSO_4$, the solvent was removed *in vacuo*. Purification by flash column chromatography over silica gel (eluent: 10-15% EtOAc in cyclohexane) afforded the title compound as a colorless solid (98 mg, 97%).

mp: 199-201 °C;

ν_{max} (ATR, cm^{-1}): 3052 (w), 1593 (w), 1483 (w), 1302 (s), 1149 (s), 854 (s), 560 (s);

^{1}H NMR (400 MHz, $CDCl_3$): δ 8.37 (dd, *J* = 7.3, 1.5 Hz, 2H), 7.80 (dd, *J* = 7.3, 1.5 Hz, 2H), 7.72 (d, *J* = 8.4 Hz, 2H), 7.68 (d, *J* = 8.3 Hz, 2H), 7.41 (app. td, *J* = 7.3, 1.5 Hz, 2H), 7.35 (app. td, *J* = 7.3, 1.5 Hz, 2H), 7.32 (d, *J* = 8.2 Hz, 2H), 7.01 (d, *J* = 8.4 Hz, 2H), 2.45 (s, 3H);

^{13}C{^{1}H} NMR (101 MHz, $CDCl_3$): δ 164.3, 158.8, 149.9, 145.6, 141.8, 140.3, 137.5, 133.7, 132.7, 129.9, 128.6, 128.5, 127.4, 125.0, 21.9;

HRMS (ESI): *m/z* [M + Na]$^+$ calcd for $C_{25}H_{19}BiNaO_5S_2$, 695.0370; found, 695.0377.

10-(4-(Allyloxy)phenyl)-10H-dibenzo[b,e][1,4]thiabismine 5,5-dioxide (**16**)

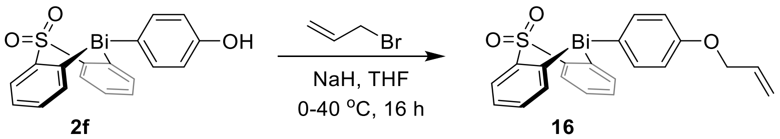

Hydroxyphenyl bismacycle **2f** (77.7 g, 0.15 mmol, 1.0 eq.) was added to a suspension of NaH (60% in mineral oil; 13.2 mg, 0.33 mol, 2.2 eq.) in anhydrous THF (2.0 mL) at 0 °C. The mixture was stirred for 15 min at 0 °C, then allyl bromide (38.9 μL, 0.45 mmol, 3.0 eq.) was added dropwise and the reaction mixture was heated at 40 °C for 16 h. The reaction mixture was cooled to 0 °C and saturated aqueous NH_4Cl (10 mL) was added dropwise. The reaction mixture was extracted with ethyl acetate (20 mL), the combined organic portions were dried over $MgSO_4$, filtered and concentrated *in vacuo*. Purification by flash column chromatography over silica gel (eluent: 5-10% EtOAc in cyclohexane) afforded the title compound as a colorless solid (82 mg, 98%).

mp: 110-112 °C;

ν_{max} (ATR, cm^{-1}): 3046 (w), 2921 (w), 2856 (w), 1574 (m), 1485 (m), 1300 (s), 1235 (s), 1148 (s), 736 (s), 562 (s);

^{1}H NMR (400 MHz, $CDCl_3$): δ 8.37 (dd, *J* = 7.7, 1.5 Hz, 2H), 7.87 (dd, *J* = 7.7, 1.5 Hz, 2H), 7.64 (d, *J* = 8.6 Hz, 2H), 7.39 (app. td, *J* = 7.7, 1.5 Hz, 2H), 7.33 (app. td, *J* = 7.7, 1.5 Hz, 2H), 6.94 (d, *J* = 8.6 Hz, 2H), 6.05 (app. ddt, *J* = 17.3, 10.6, 5.5 Hz, 1H), 5.41 (dd, *J* = 17.3, 1.6 Hz, 1H), 5.29 (dd, *J* = 10.6, 1.6 Hz, 1H), 4.53 (d, *J* = 5.5 Hz, 2H);

^{13}C{^{1}H} NMR (101 MHz, $CDCl_3$): δ 159.1, 158.5, 157.0, 141.9, 140.4, 137.7, 133.5, 133.2, 128.3, 127.2, 118.0, 117.6, 68.8;

HRMS (ESI): *m/z* $[M + Na]^+$ calcd for $C_{21}H_{17}BiNaO_3S$, 581.0595; found, 581.0568.

10-(4-Hydroxyphenyl)-10H-dibenzo[b,e][1,4]thiabismine 5,5-dioxide (**2f**)

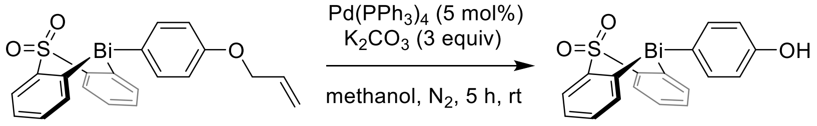

16 **2f**

$Pd(PPh_3)_4$ (8.66 mg, 0.0075 mmol, 5.0 mol%) was added to a stirred solution of allyl ether **16** (83.7 mg, 0.15 mmol, 1.0 eq.) in anhydrous MeOH (2.0 mL) under a nitrogen atmosphere. The resulting pale yellow solution was stirred for 5 min, then K_2CO_3 (62.2 mg, 0.45 mmol, 3.0 eq.) was added. After 5 h, the reaction mixture was concentrated *in vacuo*, and the residue was dissolved in EtOAc (20 mL), washed with brine, dried over Na_2SO_4, filtered and concentrated *in vacuo*. Purification by flash column chromatography over silica gel (eluent: 10-15% EtOAc in cyclohexane) afforded the title compound as a colorless solid (65 mg, 83%).

Characterisation data were consistent with those presented in *Section 2*.

10-(3-((tert-Butyldimethylsilyl)oxy)phenyl)-10H-dibenzo[b,e][1,4]thiabismine 5,5-dioxide (**17**)

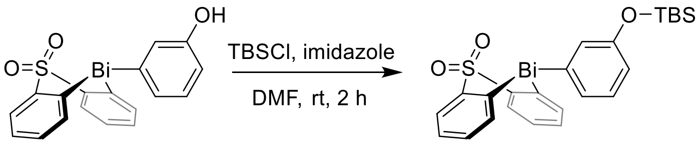

Imidazole (56.5 mg, 0.375 mmol, 2.5 eq.) was added to a solution of hydroxyphenyl bismacycle **2g** (77.7 mg, 0.15 mmol, 1.0 eq.) in DMF (2 mL) at room temperature. *tert*-Butyldimethylsilyl chloride (12.3 mg, 0.18 mmol, 1.2 eq.) was added and the resulting solution was stirred at room temperature for 2 h. After the complete consumption of bismacycle **2g** (determined by TLC), water (20 mL) was added and the mixture was extracted with EtOAc (3 × 30 mL). The combined organic portions were washed with brine (2 × 20 mL), dried over anhydrous $MgSO_4$, filtered and concentrated *in vacuo*. Purification by flash column chromatography over silica gel (eluent: 5-10% EtOAc in cyclohexane) afforded the title compound as a colorless solid (87 mg, 92%).

mp: 156-159 °C;

ν_{max} (ATR, cm^{-1}): 3043 (w), 2929 (w), 2855 (w), 1570 (m), 1466 (m), 1296 (s), 1245 (s), 1149 (s), 919 (s), 561 (s);

^{1}H NMR (400 MHz, $CDCl_3$): δ 8.40 (dd, J = 7.6, 1.4 Hz, 2H), 7.90 (dd, J = 7.6, 1.4 Hz, 2H), 7.45-7.34 (m, 6H), 7.23 (d, J = 3.0 Hz, 1H), 6.84 (app. dt, J = 7.2, 3.0 Hz, 1H), 0.89 (s, 9H), 0.03 (s, 6H);

^{13}C{^{1}H} NMR (101 MHz, $CDCl_3$): δ 166.3, 158.8, 158.7, 141.7, 137.7, 133.5, 131.6, 131.1, 130.3, 128.2, 127.1, 120.8, 25.7, 18.3, -4.3;

HRMS (ESI): *m/z* $[M + Na]^+$ calcd for $C_{24}H_{27}BiNaO_3SSi$, 655.1146; found, 655.1137.

10-(3-Hydroxyphenyl)-10H-dibenzo[b,e][1,4]thiabismine 5,5-dioxide (**2g**)

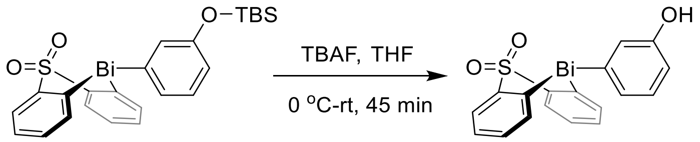

Tetrabutylammonium fluoride (50 mg, 0.18 mmol, 1.2 eq.) was added to a solution of silyl ether **17** (95 mg, 0.15 mmol, 1.0 eq.) in dry THF (2.0 mL) at 0 °C. After stirring for 45 min at room temperature, water (20 mL) and ethyl acetate (50 mL) were added. The organic portion was separated, washed with water (2 × 25 mL), dried over anhydrous $MgSO_4$, filtered and concentrated *in vacuo*. Purification by flash column chromatography over silica gel (eluent: 10-15% EtOAc in cyclohexane) afforded the title compound as a colorless solid (66 mg, 85%).

Characterisation data were consistent with those presented in *Section 2*.

6. Functional Group Interconversions of Aryl Esters (Scheme 6)

3-(5,5-Dioxido-10H-dibenzo[b,e][1,4]thiabismin-10-yl)benzoic acid (**18**)

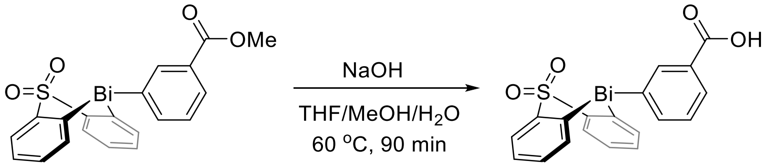

2h **18**

A solution of NaOH (1.0 M in water; 0.93 mL, 0.932 mmol, 2.0 eq.) was added to a solution of ester **2h** (261 mg, 0.466 mmol, 1.0 eq.) in THF (3 mL) and MeOH (3 mL). The solution was stirred at 60 °C for 90 min, then cooled to room temperature and water (10 mL) was added. The aqueous layer was separated and acidified with 1.0 M aqueous HCl to *p*H 4. The resulting precipitate was collected by Büchner filtration, washed with water (3 × 10 mL) and Et_2O (3 × 10 mL), then dried under a flow of air to afford the title compound as an off-white solid (180 mg, 71%).

mp: 167-168 °C;

ν_{max} **(ATR, cm^{-1}):** 3280 (br w), 3049 (w), 2957 (m), 1689 (s), 1563 (m), 1301 (s), 1287 (s), 1252 (s), 741 (s), 588 (s), 567 (w);

^{1}H NMR (400 MHz, DMSO-*d*$_6$): δ 12.84 (s, 1H), 8.47 (app. t, *J* = 1.4 Hz, 1H), 8.27-8.20 (m, 2H), 7.98-7.92 (m, 2H), 7.91 (app. dt, *J* = 7.4, 1.3 Hz, 1H), 7.87 (app. dt, *J* = 7.8, 1.5 Hz, 1H), 7.50-7.40 (m, 5H);

^{13}C{^{1}H} NMR (101 MHz, DMSO-*d*$_6$): δ 169.5, 167.8, 164.9, 143.0, 142.1, 139.2, 138.3, 133.2, 131.9, 130.3, 128.6, 127.9, 125.8;

HRMS (ESI): *m/z* [M - H]$^-$ calcd for $C_{19}H_{12}BiO_4S$, 545.0266; found, 545.0248.

Methyl 3-(5,5-dioxido-10H-dibenzo[b,e][1,4]thiabismin-10-yl)benzoate (**2h**)

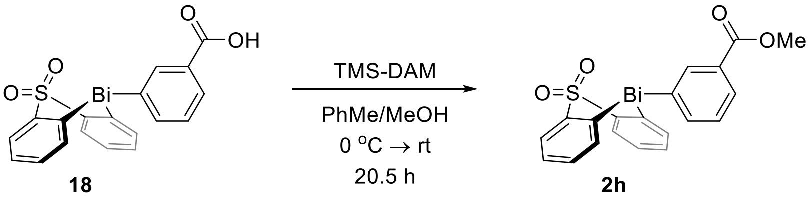

A Schlenk tube containing carboxylic acid **18** (33 mg, 0.06 mmol, 1.0 eq.) was evacuated and backfilled with anhydrous dinitrogen three times, then toluene (2 mL) and methanol (0.4 mL) were added and the mixtue was cooled to 0 °C. Trimethylsilyldiazomethane (2 M in hexane; 45.5 μL, 0.091 mmol, 1.5 eq.) was added and the reaction was stirred at 0 °C for 30 min, then at room temperature for 20 h. 1 M aqueous HCl (1 mL) was added, followed by sat. aq. $NaHCO_3$ (2 mL). The resulting mixture was extracted with ethyl acetate (10 mL) and the organic phase was washed with brine (10 mL), dried over anhydrous $MgSO_4$, filtered and concentrated *in vacuo* Purification by flash chromatography over silica gel (eluent: 10-15% EtOAc in cyclohexane) afforded the title compound as an off-white solid (19 mg, 57%).

Characterisation data were consistent with those presented in *Section 2*.

(3-(5,5-Dioxido-10H-dibenzo[b,e][1,4]thiabismin-10-yl)phenyl)(morpholino)methanone (**19**)

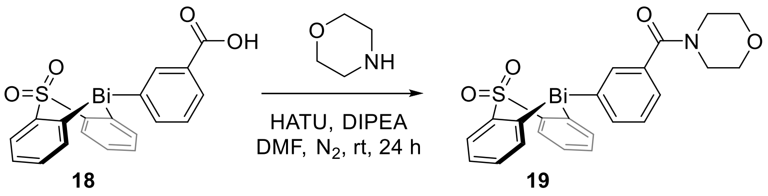

A flame-dried Schlenk tube containing carboxylic acid **18** (55 mg, 0.1 mmol, 1.0 eq.) was evacuated and backfilled with anhydrous dinitrogen three times, then anhydrous DMF (3 mL) was added. Morpholine (10.0 μL, 0.12 mmol, 1.2 eq.), HATU (46 mg, 0.12 mmol, 1.2 eq.) and *N,N*-diisopropylethylamine (44 μL, 0.25 mmol, 2.5 eq.) were added sequentially, and the resulting mixture was stirred at room temperature for 24 h. The reaction was then diluted with EtOAc (10 mL) and washed with brine (5 × 15 mL). The organic portion was dried over $MgSO_4$, filtered and concentrated *in vacuo*. Purification by flash column chromatography over silica gel (eluent: 75-80% EtOAc in cyclohexane) afforded the title compound as a colorless solid (42 mg, 64%). *This compound could not be separated from ca 10% bismacycle-derived impurity*.

mp: 185-187 °C;

ν_{max} (ATR, cm^{-1}): 2959 (w), 2924 (m), 2855 (w), 1729 (m), 1621 (s), 1453 (s), 1302 (s), 1279 (s), 1152 (s), 1115 (s), 741 (m), 589 (s);

^{1}H NMR (400 MHz, $CDCl_3$): δ 8.39 (dd, J = 7.7, 1.4 Hz, 2H), 7.87-7.81 (m, 3H), 7.73 (s, 1H), 7.47 (app. t, J = 7.4 Hz, 1H), 7.45-7.38 (m, 3H), 7.33 (app. td, J = 7.7, 1.4 Hz, 2H), 3.68 (br s, 4H), 3.44 (s, 2H), 3.27 (s, 2H);

^{13}C{^{1}H} NMR (101 MHz, $CDCl_3$): δ 170.4, 166.0, 159.2, 141.9, 140.2, 137.7, 137.6, 137.5, 133.7, 130.9, 128.5, 127.6, 127.4, 66.9, 42.6;

HRMS (ESI): *m/z* [M + Na]$^+$ calcd for $C_{23}H_{20}BiNNaO_4S$, 638.0809; found, 638.0781.

(S)-3-(5,5-Dioxido-10H-dibenzo[b,e][1,4]thiabismin-10-yl)-N-(1-hydroxy-3-methylbutan-2-yl)benzamide (**20**)

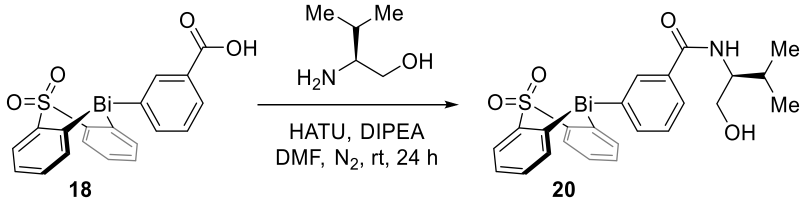

A flame-dried Schlenk tube containing carboxylic acid **18** (59 mg, 0.108 mmol, 1.0 eq.) was evacuated and backfilled with anhydrous dinitrogen three times, then anhydrous DMF (3 mL) was added. (*S*)-Valinol (13 mg, 0.13 mmol, 1.2 eq.), HATU (46 mg, 0.12 mmol, 1.2 eq.) and *N,N*-diisopropylethylamine (44 μL, 0.25 mmol, 2.5 eq.) were added sequentially, and the resulting mixture was stirred at room temperature for 24 h. The reaction was then diluted with saturated aqueous $NaHCO_3$ (10 mL) and extracted with EtOAc (3 ×10 mL). The combined organic layers were washed with brine (5 × 20 mL), dried over $MgSO_4$, filtered and concentrated *in vacuo*. Trituration with Et_2O afforded the title compound as a colorless solid (44 mg, 65%).

mp: 164-167 °C;

ν_{max} **(ATR, cm^{-1}):** 3377 (br m), 2960 (m), 2930 (w), 1640 (s), 1530 (s), 1302 (s), 1151 (s), 1088 (m), 1073 (m), 741 (s), 589 (s);

^{1}H NMR (400 MHz, $CDCl_3$): δ 8.39 (d, *J* = 7.7 Hz, 2H), 8.30 (app. t, *J* = 1.5 Hz, 1H), 7.84 (ddd, *J* = 7.4, 3.7, 1.2 Hz, 2H), 7.79 (app. dt, *J* = 7.4, 1.5 Hz, 1H), 7.75 (ddd, *J* = 7.8, 1.9, 1.2 Hz, 1H), 7.45-7.38 (m, 3H), 7.34 (app. tdd, *J* = 7.4, 3.7, 1.2 Hz, 2H), 6.27 (d, *J* = 8.4 Hz, 1H), 4.01-3.86 (m, 1H), 3.77 (d, *J* = 6.8 Hz, 2H), 2.51 (s, 1H), 2.06-1.86 (m, 1H), 1.00 (d, *J* = 6.8 Hz, 3H), 0.96 (d, *J* = 6.8 Hz, 3H);

^{13}C{^{1}H} NMR (101 MHz, $CDCl_3$): δ 168.6, 166.2, 159.1, 142.5, 141.8, 137.6, 136.9, 136.3, 133.8, 131.4, 128.5, 127.4, 127.1, 64.1, 57.7, 29.4, 19.8, 19.1;

HRMS (ESI): *m/z* $[M + Na]^+$ calcd for $C_{24}H_{24}BiNNaO_4S$, 654.1122; found, 654.1118.

10-(3-(Hydroxymethyl)phenyl)-10H-dibenzo[b,e][1,4]thiabismine 5,5-dioxide (**21**)

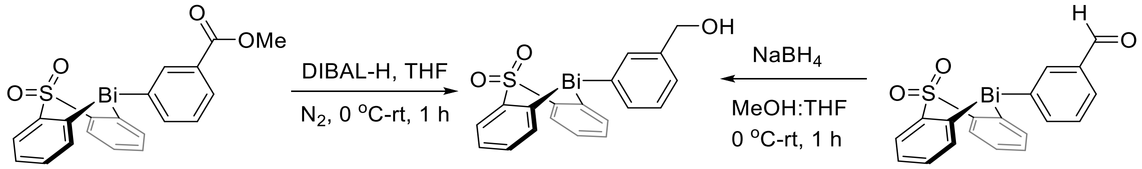

Route 1: A flame-dried Schlenk tube containing ester **2h** (336 mg, 0.6 mmol, 1.0 eq.) was evacuated and backfilled with anhydrous dinitrogen three times, then anhydrous THF (10 mL) was added. The solution was cooled to 0 °C and DIBAL-H (1.0 M in hexanes; 2.40 mL, 2.40 mmol, 4.0 eq.) was added dropwise. The resulting mixture was stirred and allowed to warm to room temperature over 1 h. The reaction was then diluted with Et_2O (20 mL) and cooled to 0 °C. Water (0.3 mL) was added dropwise followed by a solution of NaOH (1.0 M in water; 0.3 mL). The mixture was stirred at room temperature for 20 min then water (20 mL) was added and the organic layer was separated, dried over $MgSO_4$, filtered and concentrated *in vacuo* to afford the title compound as a white solid (285 mg, 89%).

Route 2: A solution of aryl aldehyde **22** (79.5 mg, 0.15 mmol, 1.0 eq.) in MeOH (1.0 mL) and THF (1.0 mL) was cooled to 0 °C, then sodium borohydride (8.0 mg, 0.195 mmol, 1.3 eq.) was added in one portion. The resulting mixture was stirred at 0 °C for 10 min and then at room temperature for 1 h. Saturated aqueous NH_4Cl was added and the mixture was stirred for 5 min, then extracted with EtOAc (3 × 20 mL). The combined organic portions were washed with brine (3 × 20 mL), dried over magnesium sulfate, filtered and concentrated *in vacuo*. Purification by flash column chromatography over silica gel (eluent: 30-35% EtOAc in cyclohexane) afforded the title compound as a colorless solid (77 mg, 97%).

mp: 149-153 °C;

ν_{max} (ATR, cm^{-1}): 3504 (br w), 3046 (w), 2920 (w), 2852 (w), 1563 (m), 1301 (s), 1286 (s), 1151 (s), 764 (m), 741 (s), 588 (s);

^{1}H NMR (400 MHz, $CDCl_3$): δ 8.38 (dd, J = 7.6, 1.4 Hz, 2H), 7.89-7.82 (m, 3H), 7.63 (app. dt, J = 6.8, 1.6 Hz, 1H), 7.43-7.37 (m, 4H), 7.33 (app. td, J = 7.3, 1.5 Hz, 2H), 4.65 (d, J = 5.0 Hz, 2H), 1.79 (t, J = 5.7 Hz, 1H);

$^{13}C\{^1H\}$ NMR (101 MHz, $CDCl_3$): δ 165.9, 158.7, 142.9, 141.8, 138.3, 137.7, 137.0, 133.6, 131.3, 128.4, 127.4, 127.3, 65.4;

HRMS (ESI): *m/z* $[M + Na]^+$ calcd for $C_{19}H_{15}BiNaO_3S$, 555.0438; found, 555.0444.

3-(5,5-Dioxido-10H-dibenzo[b,e][1,4]thiabismin-10-yl)benzaldehyde (**22**)

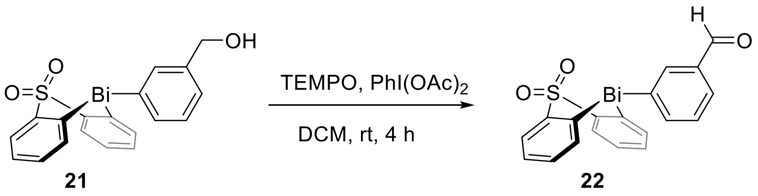

(Diacetoxyiodo)benzene (63 mg, 0.195 mmol, 1.3 eq.) and TEMPO (4.7 mg, 0.03 mmol, 0.2 eq.) were added to a stirred solution of alcohol **21** (80 mg, 0.15 mmol, 1.0 eq.) in anhydrous CH_2Cl_2 (2 mL), and the resulting mixture was stirred vigorously at rt for 4 h. The solution was then concentrated in *vacuo*. Purification by flash column chromatography over silica gel (eluent: 20-25% EtOAc in cyclohexane) afforded the title compound as a pale pink solid (52 mg, 65%).

mp: 234-237 °C;

ν_{max} (ATR, cm^{-1}): 3047 (w), 1690 (s), 1563 (m), 1303 (m), 1287 (m), 1196 (s), 765 (s), 741 (s), 588 (s);

^{1}H NMR (400 MHz, $CDCl_3$): δ 10.0 (s, 1H), 8.4 (dd, *J* = 7.7, 1.4 Hz, 2H), 8.3 (app. t, *J* = 1.4 Hz, 1H), 8.0 (app. dt, *J* = 7.4, 1.4 Hz, 1H), 7.9 (app. dt, *J* = 7.4, 1.4 Hz, 1H), 7.8 (dd, *J* = 7.7, 1.4 Hz, 2H), 7.5 (app. t, *J* = 7.4 Hz, 1H), 7.4 (app. td, *J* = 7.7, 1.4 Hz, 2H), 7.4 (app. td, *J* = 7.7, 1.4 Hz, 2H);

^{13}C{^{1}H} NMR (101 MHz, $CDCl_3$): δ 192.7, 166.4, 158.9, 145.2, 141.8, 139.6, 138.0, 137.5, 133.8, 131.6, 130.1, 128.6, 127.5;

HRMS (ESI): *m/z* [M + Na]$^+$ calcd for $C_{19}H_{13}BiNaO_3S$, 553.0282; found, 553.0283.

7. Applications of Arylbismacycles to Electrophilic Arylation (Scheme 7)

2-(3-((tert-Butyldimethylsilyl)oxy)phenoxy)pyridine (**23**)

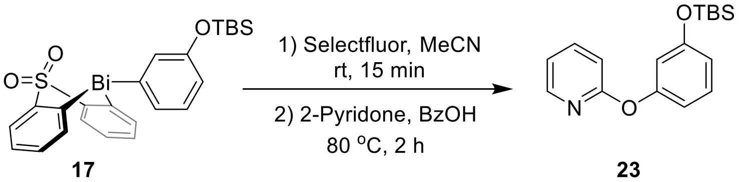

Selectfluor (70.9 mg, 0.20 mmol, 1.0 eq.) was added to a solution of silyl ether **17** (126.0 mg, 0.2 mmol, 1.0 eq.) in MeCN (2.0 mL). The reaction mixture was stirred at room temperature for 15 mins, then 2-pyridone (19 mg, 0.20 mmol, 1.0 eq.) and benzoic acid (24.4 mg, 0.2 mmol, 1.0 eq.) were added. The reaction mixture was heated at 80 °C for 2 h before cooling to room temperature. The mixture was diluted with Et_2O (10 mL), washed with 2 M aqueous. NaOH (3 × 20 mL), dried over $MgSO_4$, filtered and concentrated to dryness. Purification by flash column chromatography over silica gel (eluent: 100% cyclohexane) afforded the title compound as a yellow liquid (33 mg, 55%).

ν_{max} (ATR, cm^{-1}): 2953 (m), 2857 (w), 1711 (m), 1579 (m), 1360 (m), 1254 (s), 938 (m), 923 (s), 838 (s);

^{1}H NMR (400 MHz, $CDCl_3$): δ 8.22 (ddd, *J* = 5.0, 2.0, 0.8 Hz, 1H), 7.67 (ddd, *J* = 8.4, 7.2, 2.0 Hz, 1H), 7.23 (app. t, *J* = 8.2 Hz, 1H), 6.99 (ddd, *J* = 7.2, 5.0, 0.9 Hz, 1H), 6.86 (app. dt, *J* = 8.3, 0.9 Hz, 1H), 6.74 (ddd, *J* = 8.1, 2.3, 0.9 Hz, 1H), 6.68 (ddd, *J* = 8.1, 2.3, 0.9 Hz, 1H), 6.63 (app. t, *J* = 2.3 Hz, 1H), 0.97 (s, 9H), 0.20 (s, 6H);

^{13}C{^{1}H} NMR (101 MHz, $CDCl_3$): δ 163.7, 156.9, 155.1, 147.9, 139.4, 129.9, 118.5, 116.5, 113.9, 113.2, 111.4, 25.7, 18.2, -4.4;

HRMS (ESI): *m/z* [M + Na]$^+$ calcd for $C_{17}H_{23}NNaO_2Si$, 324.1390; found, 324.1393.

3-(Pyridin-2-yloxy)phenol (**24**)

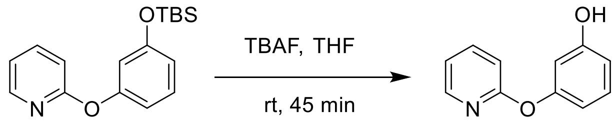

Tetrabutylammonium fluoride (65 mg, 0.25 mmol, 1.1 eq.) was added to a solution of silyl ether **23** (68 mg, 0.225 mmol, 1.0 eq.) in anhydrous THF (3.0 mL). After stirring for 45 min at room temperature, water (20 mL) and EtOAc (20 mL) were added. The organic portion was separated, washed with water (2 × 25 mL), dried over anhydrous $MgSO_4$, filtered and concentrated *in vacuo*. Purification by flash column chromatography over silica gel (eluent: 15-20% EtOAc in cyclohexane) afforded the title compound as a colourless solid (42 mg, 99%).

mp: 165-167 °C;

ν_{max} (ATR, cm^{-1}): 3061 (br w), 2922 (m), 2852 (w), 1594 (s), 1469 (s), 1127 (s), 767 (m), 693 (s);

^{1}H NMR (400 MHz, acetone-*d*$_6$): δ 8.51 (s, 1H), 8.17 (dd, *J* = 5.1, 2.0 Hz, 1H), 7.82 (ddd, *J* = 8.4, 7.2, 2.1 Hz, 1H), 7.22 (app. t, *J* = 8.1 Hz, 1H), 7.10 (dd, *J* = 6.8, 4.9 Hz, 1H), 6.96 (d, *J* = 8.3 Hz, 1H), 6.70 (dd, *J* = 8.2, 1.9 Hz, 1H), 6.65-6.57 (m, 2H);

^{13}C{^{1}H} NMR (101 MHz, acetone-*d*$_6$): δ 164.5, 159.4, 156.7, 148.5, 140.5, 130.8, 119.6, 112.9, 112.5, 112.3, 109.2;

HRMS (ESI): *m/z* $[M + H]^+$ calcd for $C_{11}H_{10}NO_2$, 188.0706; found, 188.0710.

1-(3-((tert-Butyldimethylsilyl)oxy)phenyl)naphthalen-2-ol (**25**)

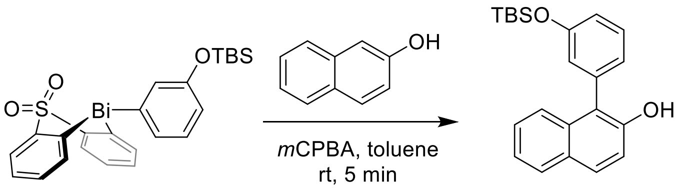

2-Naphthol (32.0 mg, 0.22 mmol, 1.1 eq.) and *m*CPBA (52 mg, 0.3 mmol, 1.5 eq.) were added sequentially to a solution of silyl ether **17** (126 mg, 0.2 mmol, 1.0 eq.) in toluene (2.0 mL) and the reaction mixture was stirred for 5 min. The reaction was quenched with MeOH (2.0 mL) and Et_2O (20 mL) was added. The reaction mixture was washed with saturated aqueous $NaHCO_3$ solution, dried over $MgSO_4$, filtered and concentrated *in vacuo*. Purification by flash column chromatography over silica gel (eluent: 100% cyclohexane) afforded the title compound as a colorless liquid (67 mg, 96%).

ν_{max} (ATR, cm^{-1}): 3540 (br w) 2954 (m), 2856 (w), 1595 (m), 1574 (m), 1388 (m), 1250 (s), 1205 (s), 1009 (m), 923 (s), 838 (s), 780 (s);

^{1}H NMR (400 MHz, $CDCl_3$): δ 7.84-7.78 (m, 2H), 7.49-7.44 (m, 2H), 7.40-7.31 (m, 2H), 7.28 (d, J = 8.8 Hz, 1H), 7.05-6.98 (m, 2H), 6.93 (dd, J = 2.5, 1.5 Hz, 1H), 5.24 (s, 1H), 1.01 (s, 9H), 0.25 (s, 6H);

^{13}C{^{1}H} NMR (101 MHz, $CDCl_3$): δ 156.8, 150.1, 135.5, 133.2, 130.8, 129.5, 128.9, 128.0, 126.5, 124.7, 123.9, 123.3, 122.9, 120.7, 120.4, 117.4, 25.7, 18.3, -4.3, -4.4;

HRMS (ESI): *m/z* $[M + Na]^+$ calcd for $C_{22}H_{26}NaO_2Si$, 373.1594; found, 373.1599.

1-(3-((tert-Butyldimethylsilyl)oxy)phenyl)naphthalen-2-yl 4-methylbenzenesulfonate (**26**)

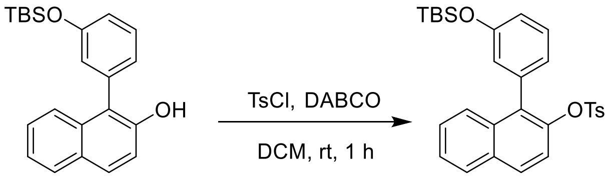

25 26

A solution of tosyl chloride (35 mg, 0.185 mmol, 1.2 eq.) in anhydrous CH_2Cl_2 (2.0 mL) was added dropwise to a solution of naphthol **25** (54 mg, 0.154 mmol, 1.0 eq.) and DABCO (35 mg, 0.308 mmol, 2.0 eq.) in anhydrous CH_2Cl_2 (2 mL) at 0 °C. The reaction was allowed to warm to room temperature and was stirred for 1 h. The mixture was then diluted with Et_2O (10 mL), and washed successively with water (10 mL), saturated aqueous $NaHCO_3$ (2 × 25 mL) and brine (25 mL). The organic portion was dried over $MgSO_4$, filtered and concentrated *in vacuo*. the solvent was removed under reduced pressure to give a solid. Purification by flash column chromatography over silica gel (eluent: 5-10% EtOAc in cyclohexane)afforded the title compound as a yellow liquid (77 mg, 99%).

ν_{max} (ATR, cm^{-1}): 3540 (br w) 2954 (w), 2929 (w), 1596 (m), 1577 (m), 1374 (s), 1172 (s), 1205 (s), 924 (s), 780 (s);

^{1}H NMR (400 MHz, $CDCl_3$): δ 7.89 (d, *J* = 9.0 Hz, 2H), 7.65 (dd, *J* = 9.1, 1.0 Hz, 1H), 7.57 (d, *J* = 8.6 Hz, 1H), 7.48 (ddd, *J* = 8.2, 6.8, 1.3 Hz, 1H), 7.39 (ddd, *J* = 8.3, 6.8, 1.5 Hz, 1H), 7.32 (d, *J* = 8.4 Hz, 2H), 7.18 (app. t, *J* = 7.9 Hz, 1H), 7.12-7.07 (m, 2H), 6.86 (app. ddt, *J* = 8.3, 2.3, 1.1 Hz, 1H), 6.67 (dd, *J* = 7.8, 1.1 Hz, 1H), 6.59 (dd, *J* = 2.5, 1.5 Hz, 1H), 2.40 (s, 3H), 0.99 (s, 9H), 0.20 (s, 3H), 0.19 (s, 3H);

^{13}C{^{1}H} NMR (101 MHz, $CDCl_3$): δ 155.4, 144.9, 144.0, 135.3, 133.2, 132.9, 132.3, 131.7, 129.6, 129.3, 129.0, 128.2, 128.1, 126.8, 126.6, 126.2, 124.0, 123.0, 121.7, 119.5, 25.8, 21.8, 18.4, -4.3, -4.3;

HRMS (ESI): *m/z* $[M + H]^+$ calcd for $C_{29}H_{33}O_4SSi$, 505.1863; found, 505.1862.

3'-((tert-Butyldimethylsilyl)oxy)-1,3-dimethyl-[1,1'-biphenyl]-2(1H)-one (**27**)

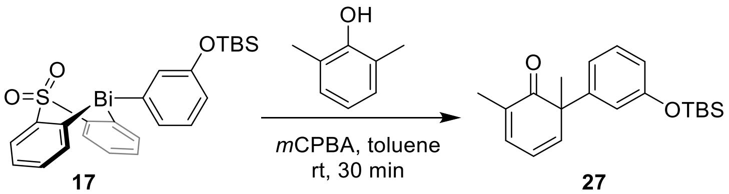

17 **27**

2,6-Dimethylphenol (41.1 mg, 0.337 mmol, 1.0 eq.) and *m*CPBA (89% purity; 64 mg, 0.337 mmol, 1.0 eq.) were added sequentially to stirred solution of silyl ether **17** (213 mg, 0.337 mmol, 1.0 eq.) in toluene (4.0 mL) at room temperature. After 30 mins, saturated aqueous $Na_2S_2O_5$ (2.0 mL) was added and the resulting mixture was stirred for 5 mins. The mixture was diluted with water (20 mL) and extracted with EtOAc (20 mL), then the organic portion was washed with saturated aqueous Na_2CO_3 (2 × 10 mL). The combined aqueous portions were extracted with EtOAc (3 × 10 mL), then the combined organic portions were dried over $MgSO_4$, filtered, filtered and concentrated *in vacuo*. Purification by flash column chromatography over silica gel (eluent: 100% cyclohexane) afforded the title compound as a colorless liquid (94 mg, 85%).

$\boldsymbol{\nu}_{\mathbf{max}}$ **(ATR, cm^{-1}):** 2931 (m), 2851 (w), 1657 (m), 1588 (m), 1477 (m), 1365 (m), 1263 (s), 959 (m), 836 (s), 764 (s);

^{1}H NMR (400 MHz, $CDCl_3$): δ 7.14 (app. t, *J* = 8.0 Hz, 1H), 6.88 (ddd, *J* = 7.9, 1.9, 0.9 Hz, 2H), 6.78 (app. t, *J* = 1.9 Hz, 1H), 6.71 (ddd, *J* = 8.0, 2.4, 1.0 Hz, 1H), 6.34 (ddd, *J* = 9.5, 1.9, 1.0 Hz, 1H), 6.24 (dd, *J* = 9.5, 5.9 Hz, 1H), 1.87 (s, 3H), 1.58 (s, 3H), 0.96 (s, 9H), 0.18 (s, 3H), 0.17 (s, 3H);

^{13}C{^{1}H} NMR (101 MHz, $CDCl_3$): δ 203.5, 155.9, 145.3, 142.3, 138.0, 132.6, 129.5, 119.8, 119.5, 118.9, 118.6, 54.1, 25.9, 23.8, 18.4, 15.9, -4.3, -4.3;

HRMS (ESI): *m/z* [M + Na]$^+$ calcd for $C_{20}H_{28}NaO_2Si$, 351.1751; found, 351.1750.

2,4-Dimethyl-[1,1'-biphenyl]-3,3'-diol (**28**)

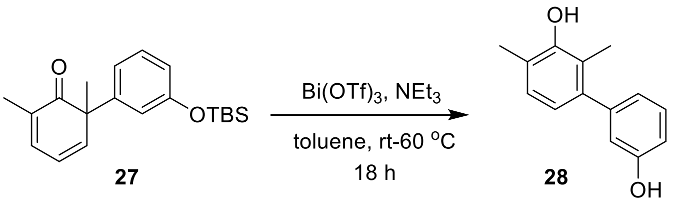

Bismuth trifluoromethanesulfonate (30.2 mg, 0.046 mmol, 20 mol%) and triethylamine (6.0 μL, 0.046 mmol, 20 mol%) were added to a solution of cyclohexadienone **27** (75.0 mg, 0.229 mmol, 1.0 eq.) in anhydrous toluene (3.0 mL). The reaction was stirred at 60 °C for 18 h, then saturated aqueous $NaHCO_3$ (5 mL) was added. The mixture was stirred for 15 mins before the aqueous layer was separated and extracted with EtOAc (3 × 20 mL). The combined organic portions were dried over $MgSO_4$, filtered, and concentrated *in vacuo*. Purification by flash column chromatography over silica gel (eluent: 10-15% EtOAc in cyclohexane) afforded the title compound as a colorless liquid (40 mg, 82%).

ν_{max} (ATR, cm^{-1}): 3420 (w), 3321 (w), 2930 (m), 2861 (m), 1699 (m), 1585 (s), 1468 (s), 1269 (s), 1199 (s), 958 (m), 767 (m);

^{1}H NMR (400 MHz, $CDCl_3$): δ 7.29 (app. t, J = 7.8 Hz, 1H), 7.05 (d, J = 7.7 Hz, 1H), 6.89 (app. dt, J = 7.6, 1.2 Hz, 1H), 6.84 (ddd, J = 8.1, 2.6, 1.0 Hz, 1H), 6.83-6.76 (m, 2H), 4.98 (s, 1H), 4.80 (s, 1H), 2.33 (s, 3H), 2.19 (s, 3H);

^{13}C{^{1}H} NMR (101 MHz, $CDCl_3$): δ 155.3, 152.4, 143.6, 141.0, 129.4, 127.9, 122.2, 122.1, 121.8, 120.9, 116.5, 113.8, 16.1, 13.3;

HRMS (ESI): *m/z* $[M + H]^+$ calcd for $C_{14}H_{15}O_2$, 215.1067; found, 215.1069.

3'-((tert-Butyldimethylsilyl)oxy)-6-hydroxy-4,5-dihydro-[1,1'-biphenyl]-2(3H)-one (**29**)

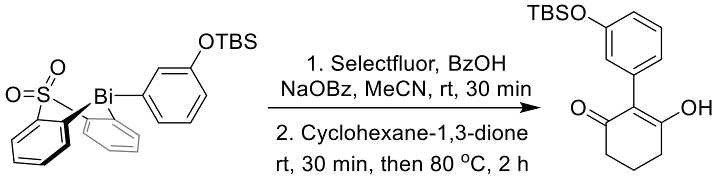

17 **29**

Selectfluor (70.8 mg, 0.2 mmol, 1.0 eq.), sodium benzoate (57.6 mg, 0.4 mmol, 2.0 eq.) and benzoic acid (24.4 mg, 0.2 mmol, 1.0 eq.) were added to a solution of silyl ether **17** (126.0 mg, 0.2 mmol, 1.0 eq.) in MeCN (2.0 mL). The reaction was stirred at room temperature for 30 min, then diluted with toluene (6.0 mL). 1,3-Cyclohaxanedione (22.4 mg, 0.2 mmol, 1.0 eq.) was added and the reaction mixture was stirred at room temperature for 30 min then heated at 80 °C for 2 h. After cooling to room temperature, the reaction mixture was diluted with EtOAc (20 mL) and extracted with saturated aqueous $NaHCO_3$ solution (3 × 10 mL). The combined aqueous portions were acidified to *p*H 1 with 2 M aqueous HCl then extracted with EtOAc (3 × 20 mL). The combined organic portions were dried over $MgSO_4$, filtered and concentrated *in vacuo*. Purification by flash column chromatography over silica gel (eluent: 15-20% EtOAc in cyclohexane) afforded the title compound as a colorless liquid (41 mg, 65%).

ν_{max} (ATR, cm^{-1}): 3396 (br m), 2956 (m), 2858 (w), 1710 (s), 1586 (m), 1466 (m), 1427 (s), 1242 (s), 1136 (s), 980 (s), 831 (s), 779 (s);

^{1}H NMR (400 MHz, $CDCl_3$): δ 7.32 (app. t, *J* = 7.9 Hz, 1H), 6.84 (ddd, *J* = 8.2, 2.5, 1.0 Hz, 1H), 6.81 (app. dt, *J* = 7.5, 1.3 Hz, 1H), 6.71 (dd, *J* = 2.5, 1.6 Hz, 1H), 6.16 (s, 1H), 2.63 (t, *J* = 6.3 Hz, 2H), 2.52 (t, *J* = 6.6 Hz, 2H), 2.10 (app. p, *J* = 6.4 Hz, 2H), 1.00 (s, 9H), 0.23 (s, 6H);

^{13}C{^{1}H} NMR (101 MHz, $CDCl_3$): δ 196.6, 170.6, 156.4, 132.1, 130.4, 123.2, 122.4, 119.9, 117.9, 37.0, 27.9, 25.7, 20.4, 18.2, -4.4;

HRMS (ESI): *m/z* [M + Na]$^+$ calcd for $C_{18}H_{26}NaO_3Si$, 341.1543; found, 341.1538.

3'-((tert-Butyldimethylsilyl)oxy)-6-oxo-3,4,5,6-tetrahydro-[1,1'-biphenyl]-2-yl methyl carbonate (**30**)

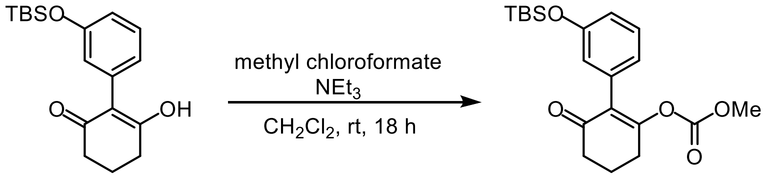

Triethylamine (47 μL, 0.33 mmol, 2.2 eq.) and methyl chloroformate (14 μL, 0.18 mmol, 1.2 eq.) were added sequentially to a solution of dione **29** (48 mg, 0.15 mmol, 1.0 eq.) in anhydrous CH_2Cl_2 (2.0 mL) at room temperature. The reaction was stirred at room temperature for 18 hours, then diluted with CH_2Cl_2 (10 mL) and washed with water (3 × 10 mL). The organic portion was dried over $MgSO_4$, filtered and concentrated *in vacuo* to afford the compound as a light yellow liquid (50 mg, 89%).

ν_{max} (ATR, cm^{-1}): 2954 (m), 2854 (m), 1760 (s), 1679 (s), 1578 (m), 1438 (s), 1231 (s), 1171 (s), 937 (s), 839 (s), 781 (s);

^{1}H NMR (400 MHz, $CDCl_3$): δ 7.20 (app. t, *J* = 8.0 Hz, 1H), 6.78 (ddd, *J* = 8.0, 2.5, 1.0 Hz, 1H), 6.72 (ddd, *J* = 7.6, 1.6, 1.0 Hz, 1H), 6.62 (dd, *J* = 2.5, 1.6 Hz, 1H), 3.70 (s, 3H), 2.74 (t, *J* = 6.2 Hz, 2H), 2.58 (t, *J* = 6.2 Hz, 2H), 2.15 (app. p, *J* = 6.4 Hz, 2H), 0.97 (s, 9H), 0.18 (s, 6H);

^{13}C{^{1}H} NMR (101 MHz, $CDCl_3$): δ 197.7, 163.9, 155.3, 151.9, 132.1, 130.2, 129.0, 122.8, 121.7, 119.8, 55.7, 37.6, 29.8, 28.6, 25.8, 20.7, -4.4;

HRMS (ESI): *m/z* $[M + H]^+$ calcd for $C_{20}H_{29}O_5Si$, 377.1779; found, 377.1776.

8. Attempted Functionalizations with Reactive Organometallic Reagents

Mg-Br Exchange / Borylation of 3-Bromophenyl Bismacycle **2c**

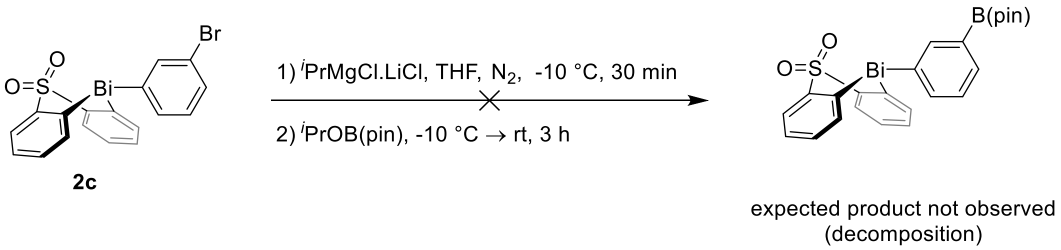

According to a modified literature procedure:[4] An oven-dried reaction tube containing a magnetic stir bar was sealed, flame-dried under vacuum, and cooled to rt under an atmosphere of anhydrous dinitrogen. 3-Bromophenyl bismacycle **2c** (116 mg, 0.2 mmol, 1.0 eq.) was added, then the tube was evacuated and backfilled with anhydrous dinitrogen three times. Anhydrous THF (2.0 mL) was added, and the resulting solution was cooled to -10 °C. ^{i}PrMgCl·LiCl (1.3 M solution in THF; 170 μL, 0.22 mmol, 1.1 eq.) was added dropwise, and the reaction was stirred at -10 °C for 30 min. In a second flame-dried reaction tube (evacuated and backfilled with anhydrous dinitrogen), 2-isopropoxy-4,4,5,5-tetramethyl-1,3,2-dioxaborolane (61 μL, 55.8 mg, 0.3 mmol, 1.5 eq.) was dissolved in THF (2 mL) then cooled to -10 °C. The Grignard solution was then added to this dropwise at -10 °C, and the resulting mixture was stirred at rt for 3 h. A small aliquot was quenched with sat. aq. NH_4Cl and extracted with EtOAc to check the progress of the reaction; analysis by ^{1}H NMR spectroscopy indicated that the desired product was not formed, and that the bismacycle had decomposed to form a complex mixture of species.

Mg-Br Exchange / Methoxycarbonylation of 3-Bromophenyl Bismacycle **2c**

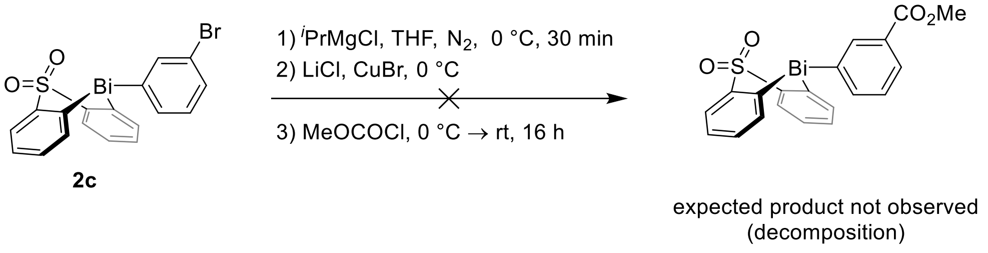

According to a modified literature procedure:[5] An oven-dried reaction tube containing a magnetic stir bar was sealed, flame-dried under vacuum, and cooled to rt under an atmosphere of anhydrous dinitrogen. 3-Bromophenyl bismacycle **2c** (116 mg, 0.2 mmol, 1.0 eq.) was added, then the tube was evacuated and backfilled with anhydrous dinitrogen three times. Anhydrous THF (2.0 mL) was added, and the resulting solution was cooled to 0 °C. ^{i}PrMgCl (2.0 M solution in THF; 110 μL, 0.22 mmol, 1.1 eq.) was added dropwise, and the reaction was stirred at 0 °C for 30 min. In a second flame-dried reaction tube (evacuated and backfilled with anhydrous dinitrogen), LiBr (35 mg, 0.4 mmol, 2.0 eq.) and CuBr (28 mg, 0.2 mmol, 1.0 eq.) were dissolved in THF (2 mL) then cooled to 0 °C. The Grignard solution was added to this dropwise at 0 °C, then methyl chloroformate (15 μL, 19 mg, 0.2 mmol, 1.0 eq) was added quickly and the resulting mixture was stirred at rt for 16 h. A small aliquot was quenched with sat. aq. NH_4Cl and extracted with EtOAc to check the progress of the reaction; analysis by ^{1}H NMR spectroscopy indicated that the desired product was not formed, and that the bismacycle had decomposed to form a complex mixture of species.

Shi-type Cyclopropanation of Styrenyl Bismacycle **2d**

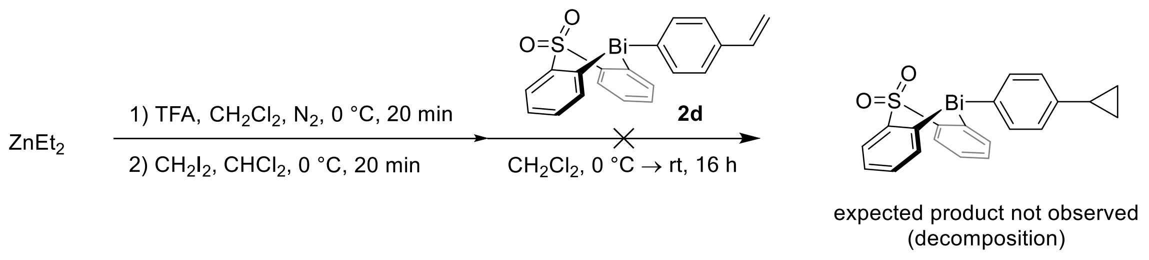

According to a modified literature procedure:[6] An oven-dried reaction tube containing a magnetic stir bar was sealed, flame-dried under vacuum, and cooled to rt under an atmosphere of anhydrous dinitrogen. Anhydrous CH_2Cl_2 (1.0 mL) was added, followed by Et_2Zn (0.9 M in hexane; 440 μL, 0.4 mmol, 2.0 eq.). The resulting solution was stirred and cooled to 0 °C, then

a solution of TFA (31 μL, 0.4 mmol, 2.0 eq.) in anhydrous CH_2Cl_2 (1 mL) was added dropwise and the reaction mixture was stirred for 20 min. A solution of CH_2I_2 (33 μL, 0.4 mmol, 2.0 eq.) in anhydrous CH_2Cl_2 (1 mL) was added dropwise at 0°C, and the reaction mixture was stirred for a further 20 min. Finally, a solution of styrenyl bismacycle **2d** (106 mg, 0.2 mmol, 1.0 eq.) in anhydrous CH_2Cl_2 (1 mL) was added dropwise at 0 °C. The reaction mixture was gradually warmed to room temperature and stirred for an additional 16 h. A small aliquot was quenched with sat. aq NH_4Cl and extracted with DCM to check the progress of the reaction.

A small aliquot was quenched with sat. aq. NH_4Cl and extracted with CH_2Cl_2 to check the progress of the reaction; analysis by ^{1}H NMR spectroscopy indicated that the desired product was not formed, and that the bismacycle had undergone formal protodebismuthation to give the corresponding iodobismacycle.[7]

9. Attempted Functionalizations *via* Photoredox Catalysis

Thioetherification of 4-Iodophenyl Bismacycle **2b**

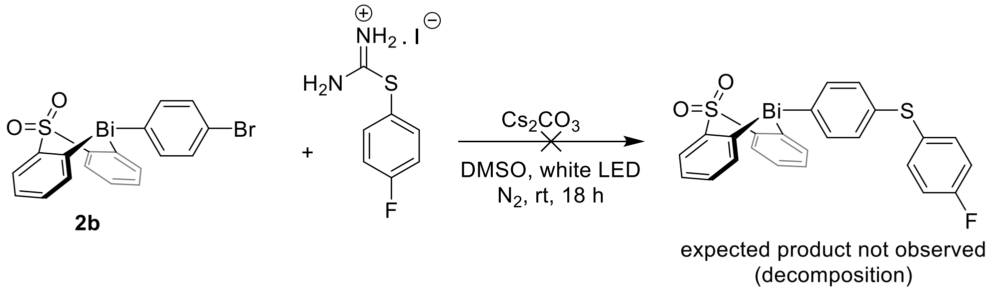

According to literature procedure:[8] An oven-dried microwave tube containing a stir bar, Cs_2CO_3 (195.5 mg, 0.6 mmol, 3.0 eq.), 4-iodophenyl bismacycle **2b** (116.0 mg, 0.2 mmol, 1.0 eq.) and 2-(4-fluorophenyl)isothiouronium iodide (0.3 mmol, 89.4 mg, 1.5 eq.) was evacuated and back-filled with anhydrous dinitrogen three times. Degassed DMSO (2 mL) was then added and the reaction was stirred at room temperature under irradiation with white light (16 W LEDs; 6200 K) for 18 h. Analysis of an aliquot by ^{1}H NMR spectroscopy indicated that the desired product was not formed, and that the bismacycle had decomposed to form a complex mixture of species.

As a control reaction, a solution of iodophenyl bismacycle **2b** (29.0 mg, 0.05 mmol) in degassed DMSO (0.5 mL) was stirred at room temperature under irradiation with white light (16 W LEDs; 6200 K) for 18 h. >95% of the starting material was recovered, demonstrating the stability of bismacycle towards direct irradiation with visible light.

Iodoamination of Styrenyl Bismacycle **2d**

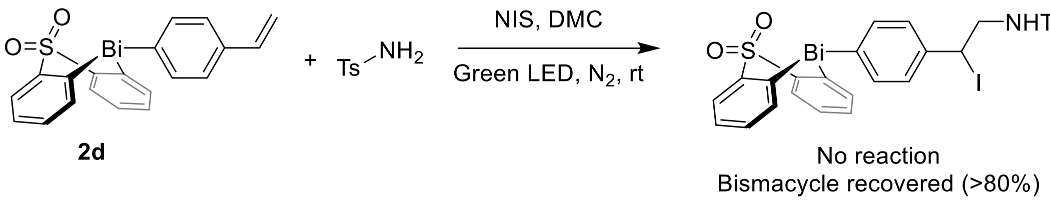

According to literature procedure:[9] A flame-dried Schlenk tube containing a stir bar, styrenyl bismacycle **2d** (79.2 mg, 0.15 mmol, 1.0 eq.), tosylamide (25.7 mg, 0.15 mmol, 1.0 eq.), NIS (33.7 mg, 0.15 mmol, 1.0 eq.) and DMC (0.6 mL) was degassed by three consecutive freeze-

pump-thaw cycles. It was then irradiated with a with green light (16 W LEDs; 525 nm) while the reaction mixture was magnetically stirred at room temperature. Analysis of an aliquot by ^{1}H NMR spectroscopy indicated that the desired product was not formed, and that the bismacycle was unreacted (>80%).

10.Summary of Reaction Conditions Employed

Note: the (in)compatibility of reaction conditions with aryl bismacycles is highly dependent on the nature and position of any functionality present on the exocyclic aryl moiety of the substrate / product. The following list therefore summarizes only the specific cases that we have tested.

Summary of reaction conditions compatible with aryl bismacycles:

Substitutions

- NaH, MeI, rt
- NaH, allyl bromide, rt or 40 °C
- DMAP, ArNCO, rt
- DABCO, TsCl, rt
- TBSCl, imidazole, rt
- TBAF, rt
- Aq. NaOH, 60 °C
- TMS-diazomethane
- Amine, HATU, DIPEA, rt

Transition Metal Catalysis

- Pd-cat., aq. K_3PO_4, rt
- Pd-cat, Na_2CO_3, 90 °C
- Pd-cat., Cu-cat., NEt_3, rt
- Pd-cat., NaO^{t}Bu, 80 °C
- Cu-cat., K_3PO_4, 100 °C
- Pd.-cat, K_2CO_3, MeOH, rt
- Grubbs-II, 40 °C

Oxidations

- AD-mix-α, rt
- Aq. NaOH, H_2O_2, rt
- Cat. TEMPO, PhI(OAc)$_2$, rt
- Mn-cat., pyridine *N*-oxide, NaOCl, rt

Reductions

- 9-BBN, rt
- DIBAL-H, rt
- $NaBH_4$, MeOH, rt
- Pd/C, H_2 (1 atm.), rt
- $(Ph_3P)_3RhCl$, H_2 (1 atm.), rt

Summary of reaction conditions incompatible with aryl bismacycles:

Transition Metal Catalysis

- Pd-cat., B_2pin_2, potassium 2-ethylhexanoate, 35 °C
- Pd-cat., B_2pin_2, KOAc, 80 °C
- Pd-cat., $B_2(OH)_4$, KOAc, 80 °C
- Pd-cat., HBpin, rt
- Ni-cat, NaOH, hydrazine, 60 °C

Photochemistry

- Thiophenol, Cs_2CO_3, white light, rt
- Tosamide, NIS, green light, rt

11.References

(1) Jurrat, M.; Maggi, L.; Lewis, W.; Ball, L. T. Modular Bismacycles for the Selective C–H Arylation of Phenols and Naphthols. *Nature Chem.* **2020**, *12* (3), 260–269. https://doi.org/10.1038/s41557-020-0425-4.

(2) Sakurai, N.; Mukaiyama, T. Direct α-Oxytosylation of Ketones by Using Pentavalent Organobismuth Reagents. *Chem. Lett.* **2008**, *37* (4), 388–389. https://doi.org/10.1246/cl.2008.388.

(3) Senior, A.; Ruffell, K.; Ball, L. T. Meta-Selective C-H Arylation of Phenols via Regiodiversion of Electrophilic Aromatic Substitution. *Nature Chem.* **2023**, *15*, 386–394. https://doi.org/10.1038/s41557-022-01101-0.

(4) Myslinska, M.; Heise, G. L.; Walsh, D. J. Practical and Efficient Applications of Novel Dioxaborolanes and Dioxaborinanes in the Synthesis of Corresponding Boronates and Their Use in the Palladium-Catalyzed Cross Coupling Reactions. *Tetrahedron Lett.* **2012**, *53* (24), 2937–2941. https://doi.org/https://doi.org/10.1016/j.tetlet.2012.03.048.

(5) Bottalico Vito; Marchese, Giuseppe; Punzi, Angela, D. F. A New Versatile Synthesis of Esters from Grignard Reagents and Chloroformates. *Synlett* **2007**, *2007* (06), 974–976. https://doi.org/10.1055/s-2007-973861.

(6) Roy, A.; Bonetti, V.; Wang, G.; Wu, Q.; Klare, H. F. T.; Oestreich, M. Silylium-Ion-Promoted Ring-Opening Hydrosilylation and Disilylation of Unactivated Cyclopropanes. *Org. Lett.* **2020**, *22* (3), 1213–1216. https://doi.org/10.1021/acs.orglett.0c00173.

(7) Worrell, B. T.; Ellery, S. P.; Fokin, V. V. Copper(I)-Catalyzed Cycloaddition of Bismuth(III) Acetylides with Organic Azides: Synthesis of Stable Triazole Anion Equivalents. *Angew. Chem. Int. Ed.* **2013**, *52* (49), 13037–13041. https://doi.org/https://doi.org/10.1002/anie.201306192.

(8) Swan, C.; Maggi, L.; Park, M.; Taylor, S.; Shepherd, W.; Ball, L. T. Generation of Thiyl Radicals from Air-Stable, Odorless Thiophenol Surrogates: Application to Visible-Light Promoted C-S Cross-Coupling. *Synthesis* **2022**, *54* (15), 3399–3408. https://doi.org/10.1055/s-0041-1737816.

(9) Engl, S.; Reiser, O. Catalyst-Free Visible-Light-Mediated Iodoamination of Olefins and Synthetic Applications. *Org. Lett.* **2021**, *23* (14), 5581–5586. https://doi.org/10.1021/acs.orglett.1c02035.

12.NMR Spectra

2a - ¹H NMR (400 MHz, $CDCl_3$):

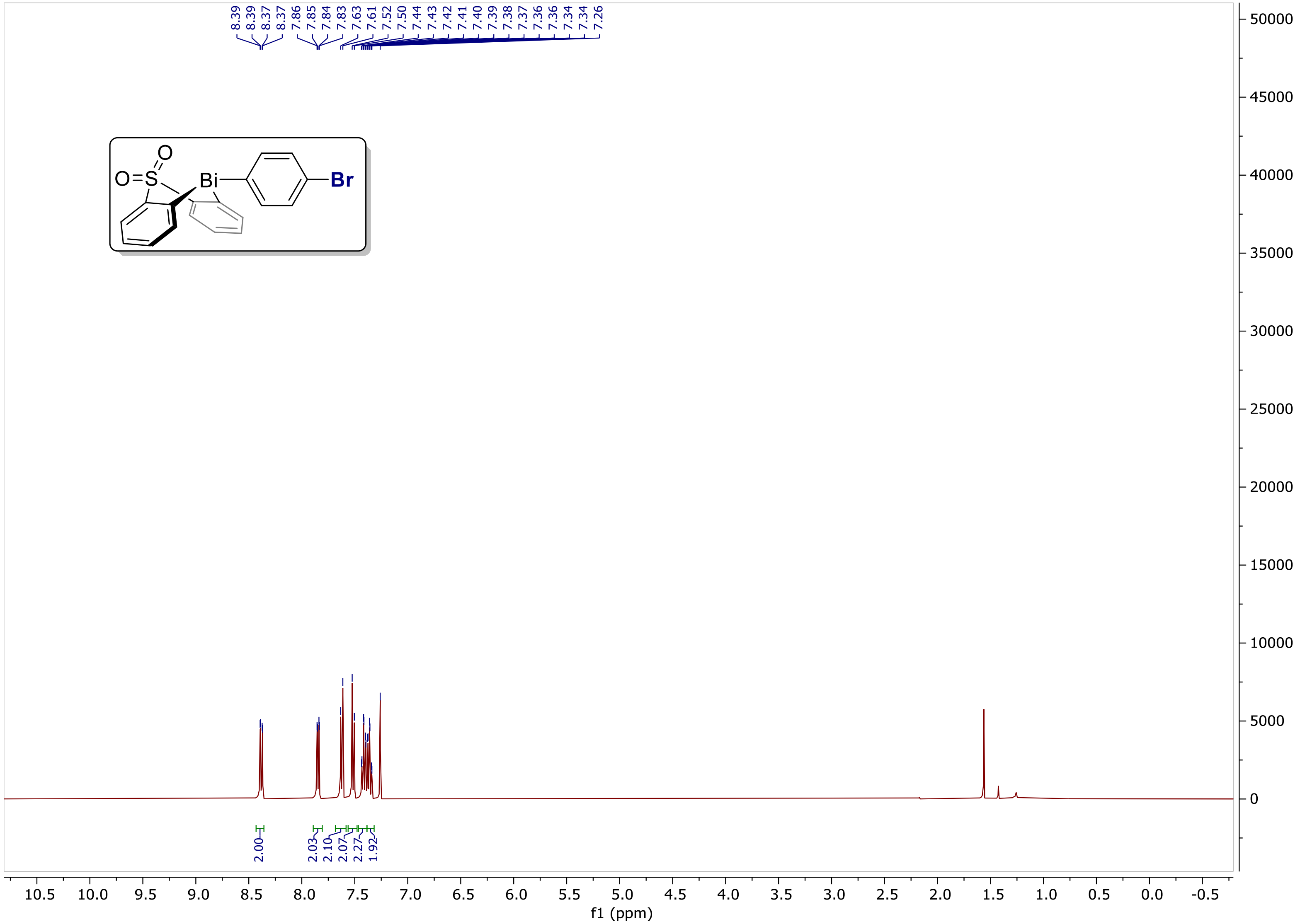

2b - ^{1}H NMR (400 MHz, $CDCl_3$):

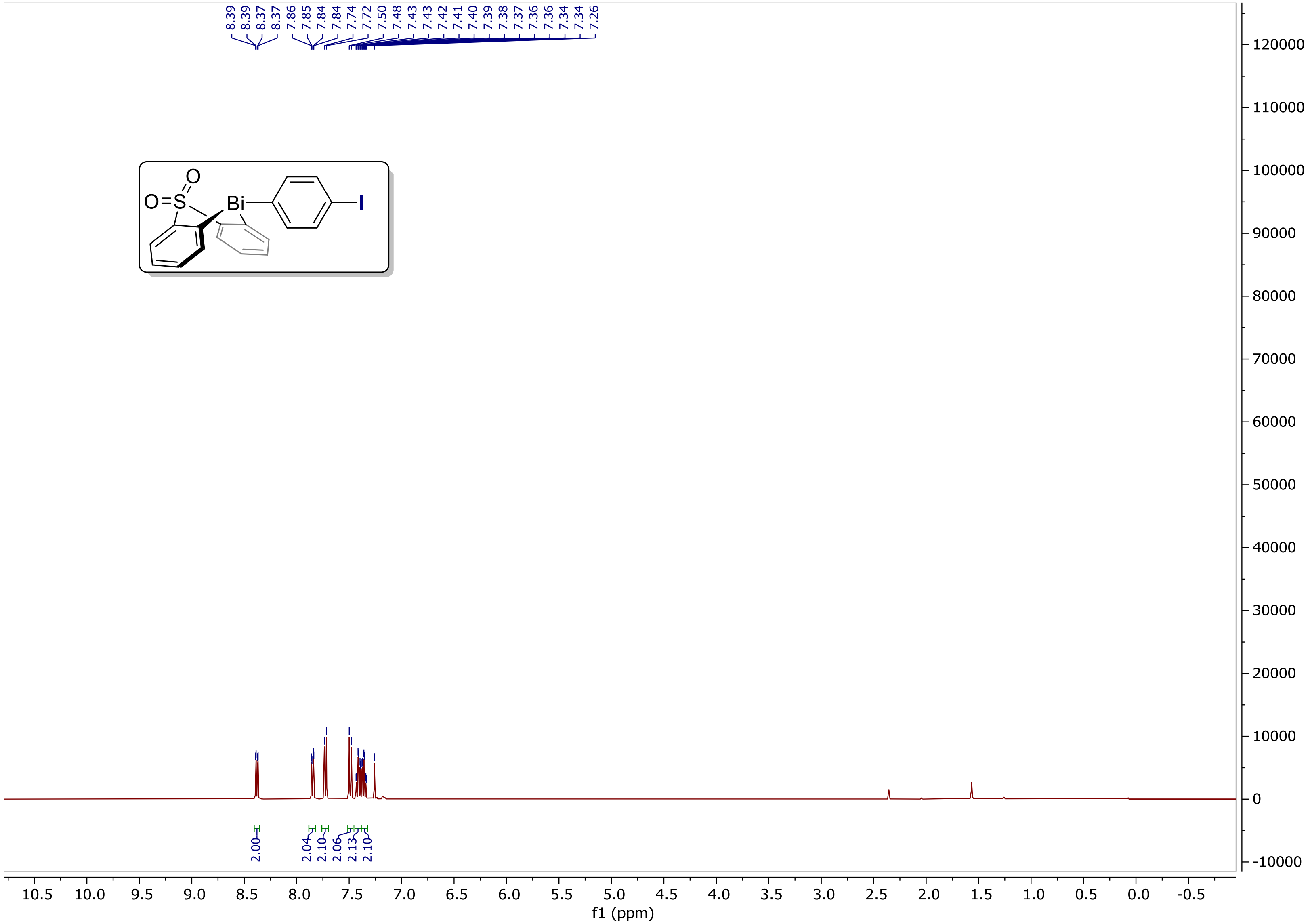

2c - ^{1}H NMR (400 MHz, $CDCl_3$):

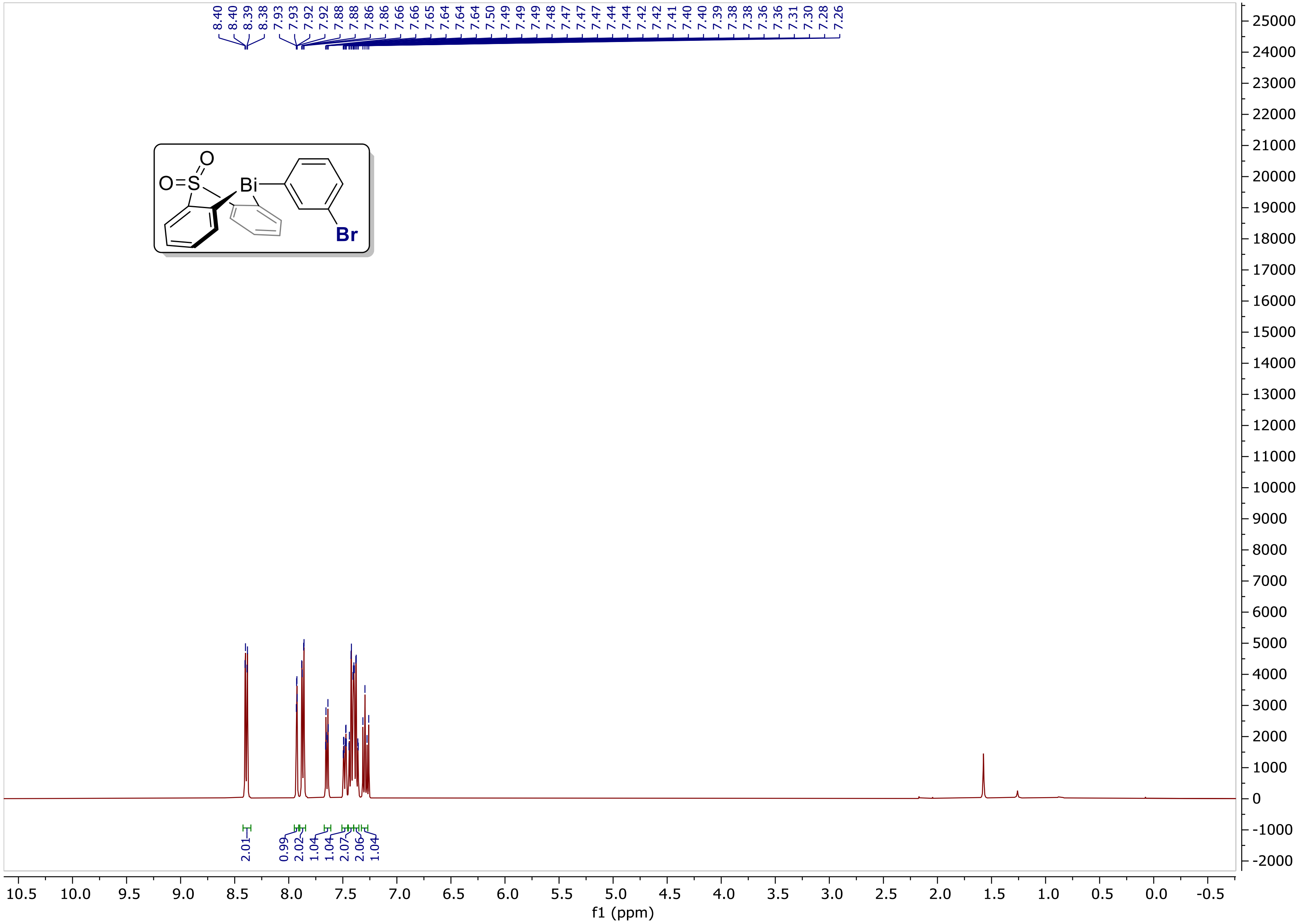

2d - ^{1}H NMR (400 MHz, $CDCl_3$):

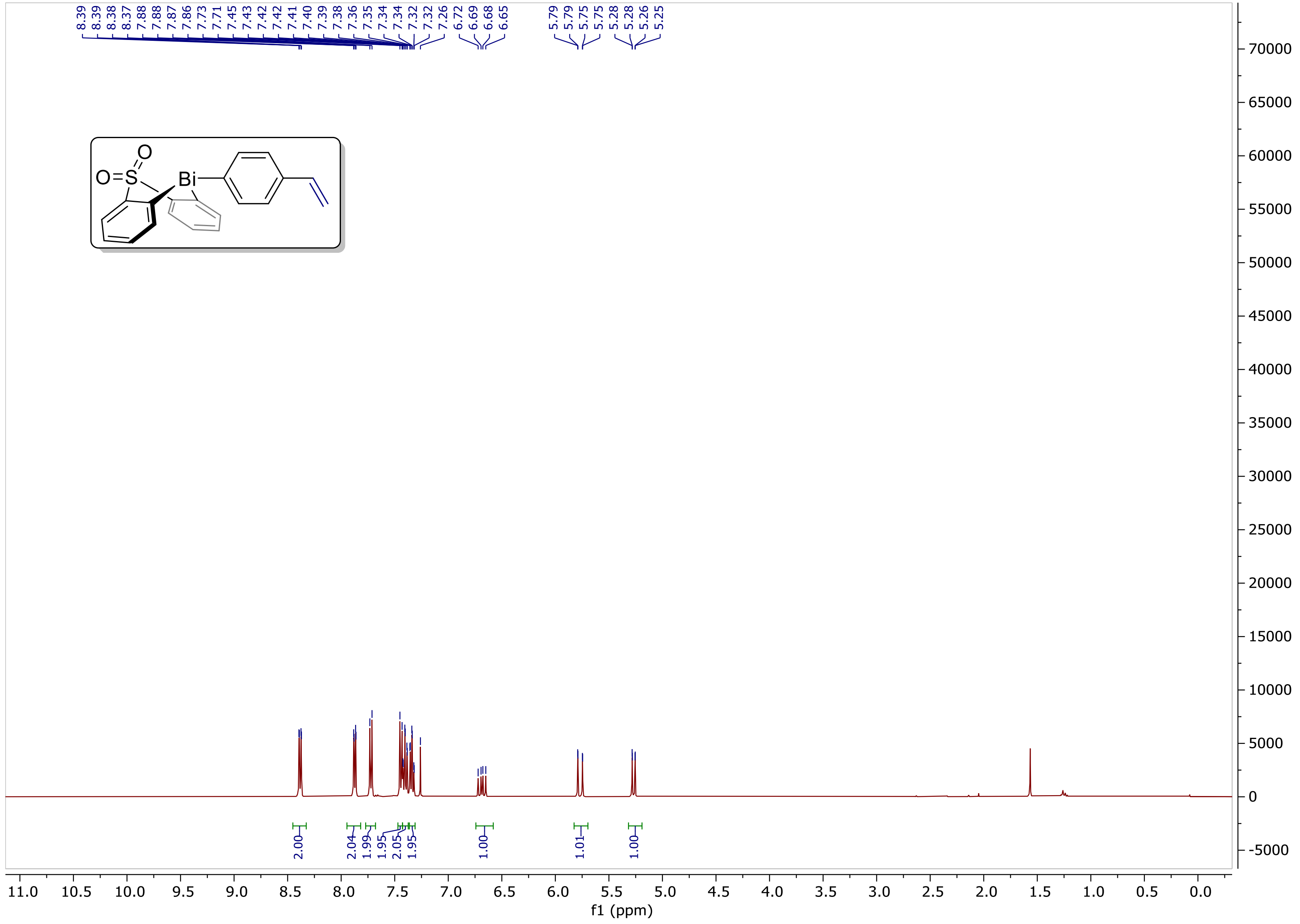

2e - ^{1}H NMR (400 MHz, CDCl3):

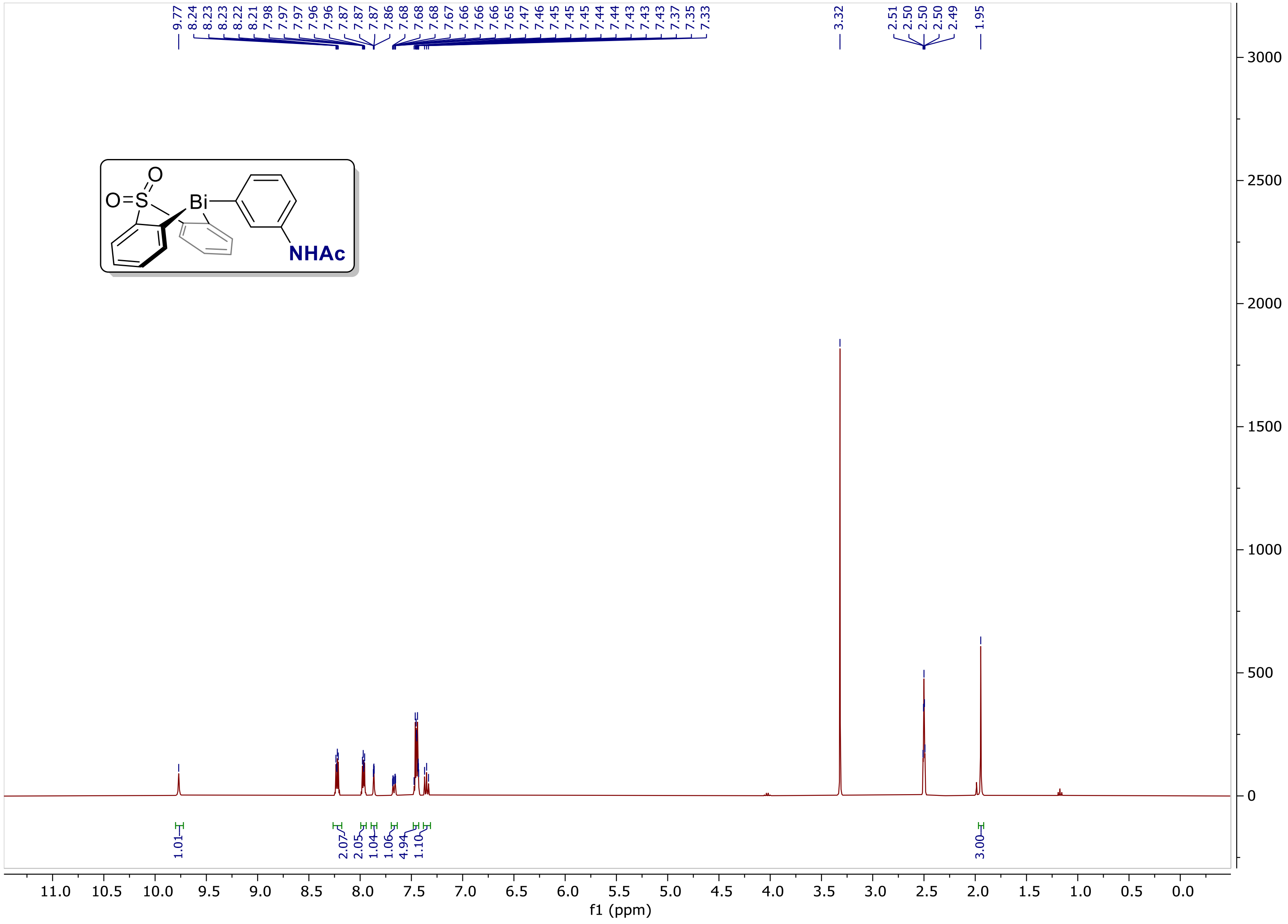

2f - ^{1}H NMR (400 MHz, DMSO-d_6):

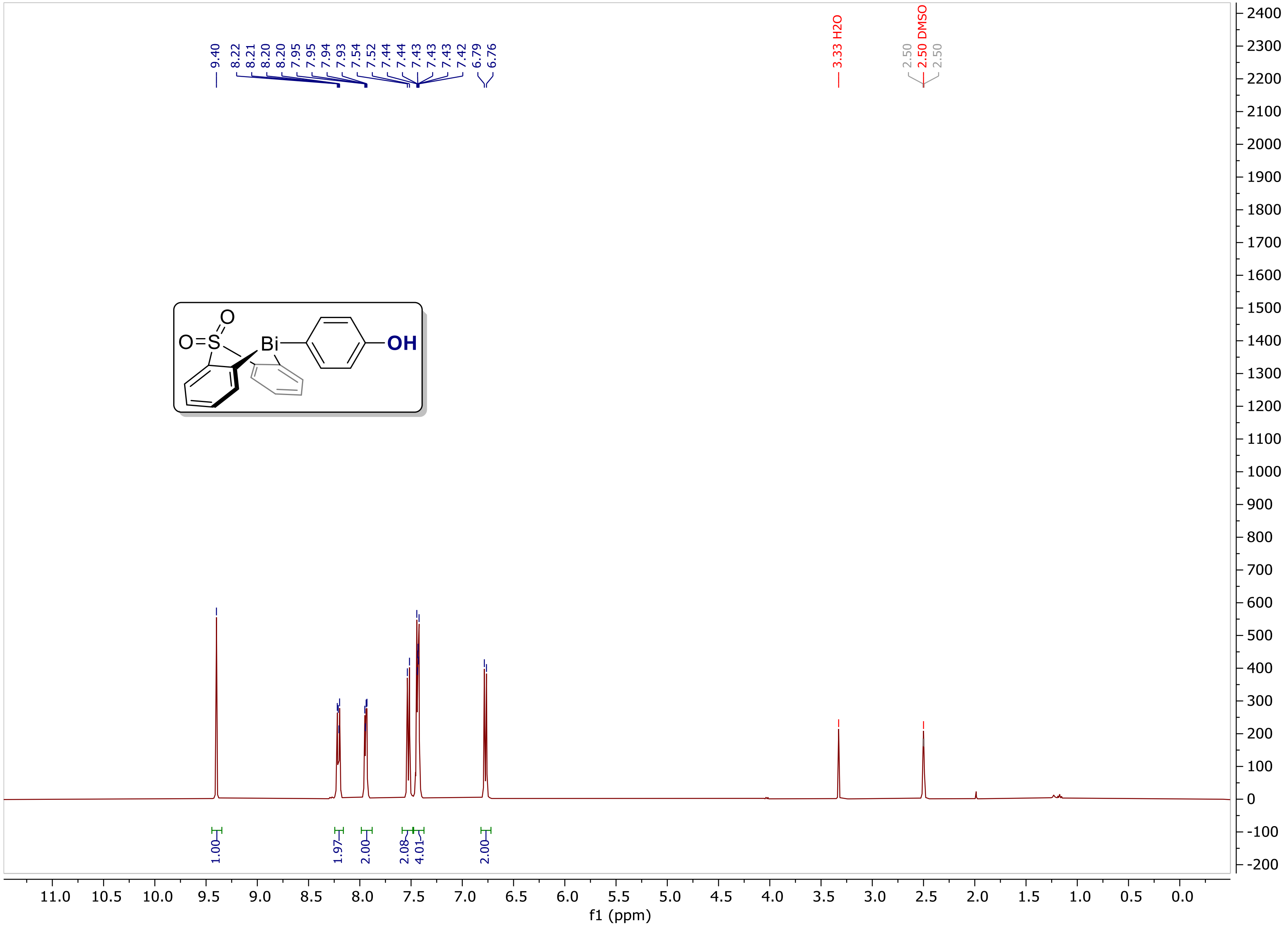

2f - $^{13}C\{^1H\}$ NMR (101 MHz, DMSO-d_6):

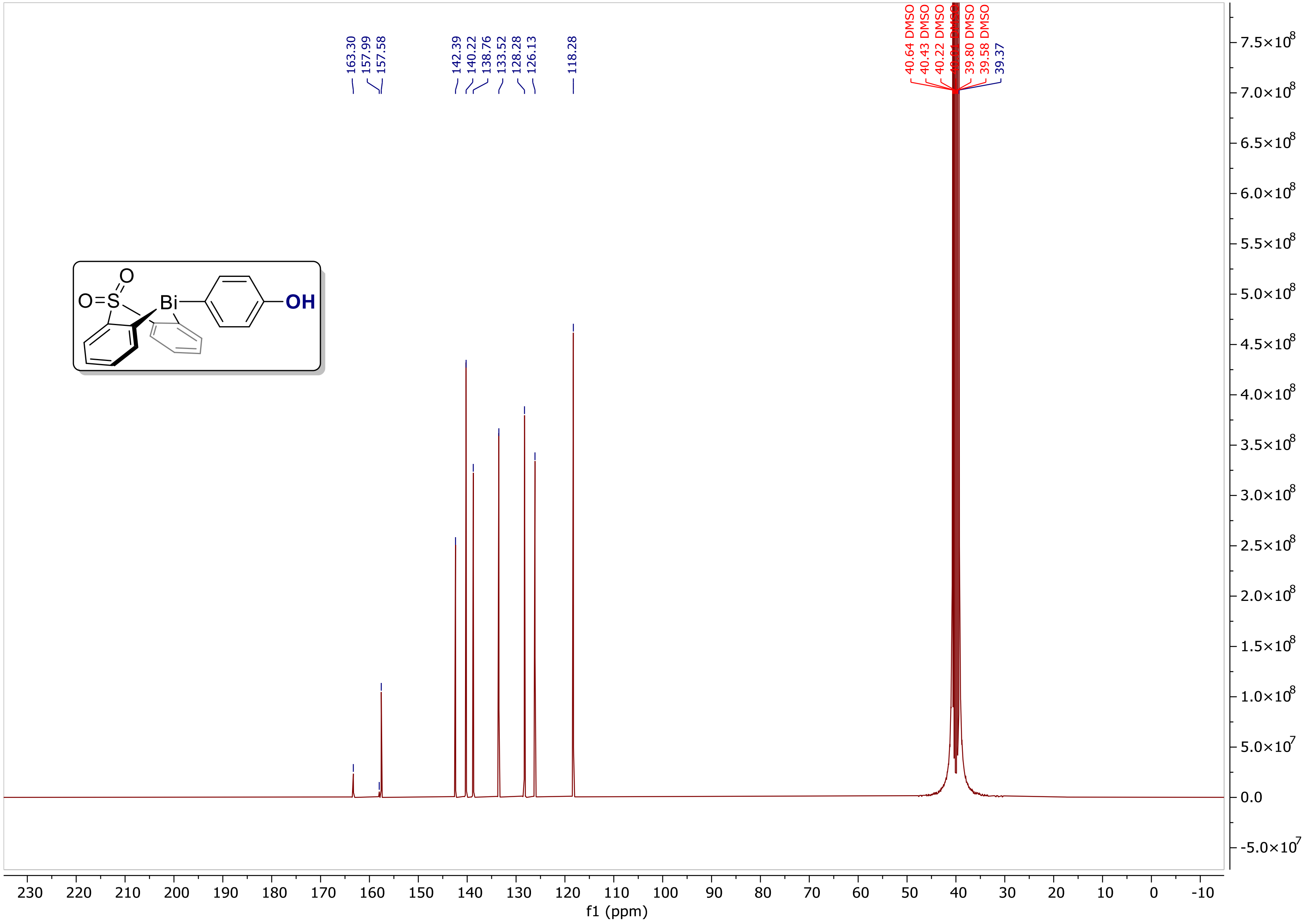

2g - ^{1}H NMR (400 MHz, $CDCl_3$):

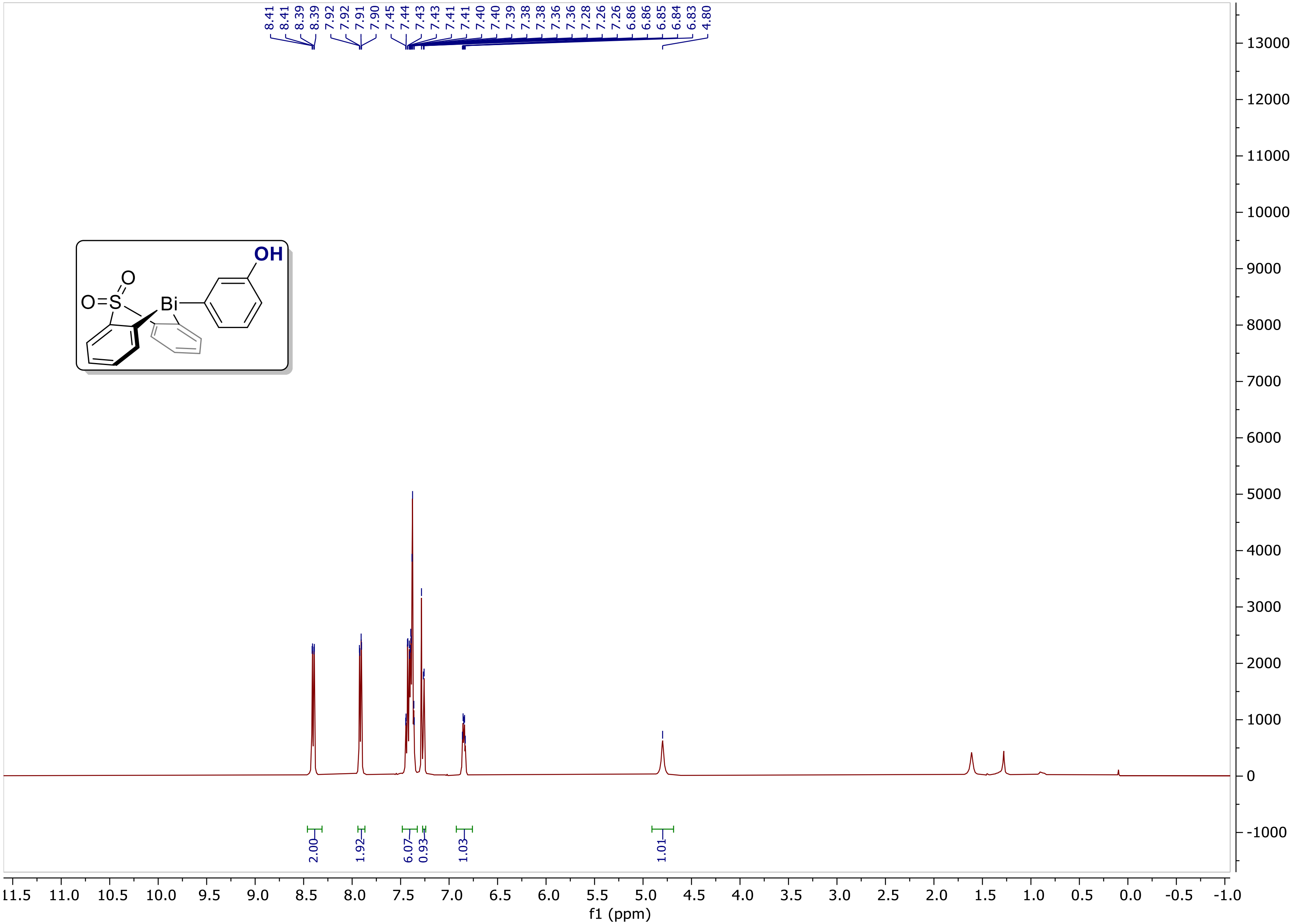

2g - $^{13}C\{^{1}H\}$ NMR (101 MHz, DMSO-d_6):

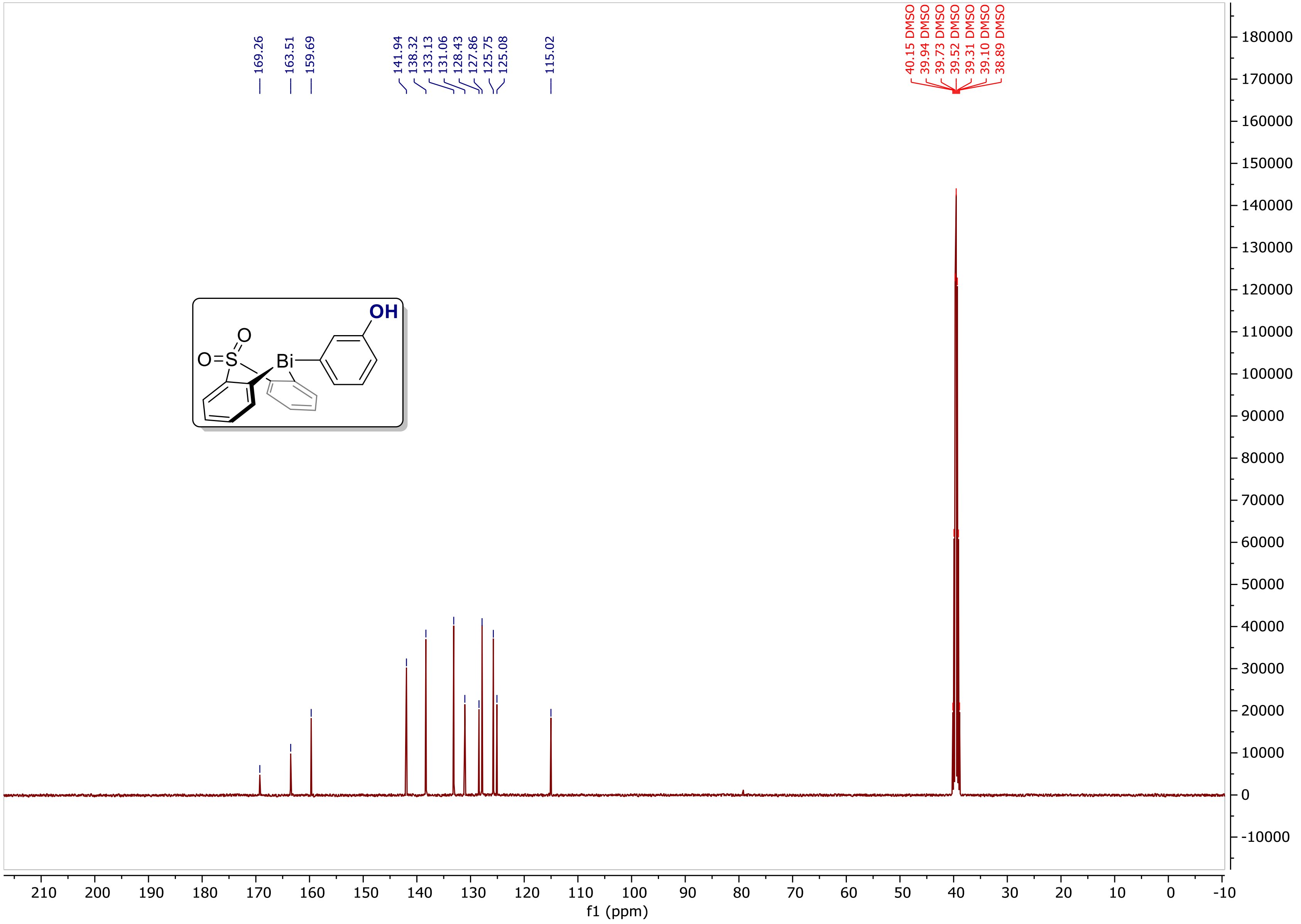

2h - ^{1}H NMR (400 MHz, $CDCl_3$):

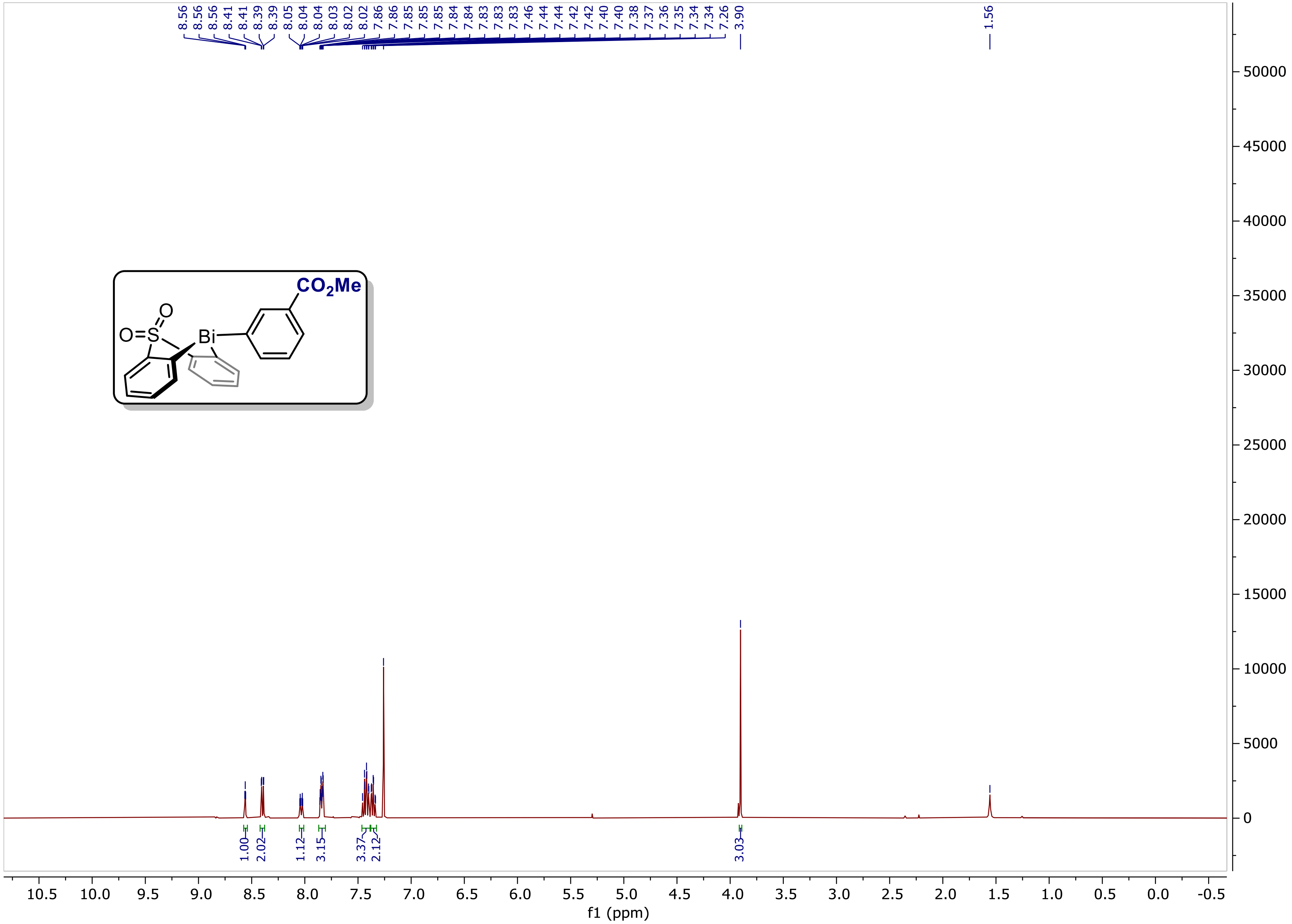

2h - $^{13}C\{^1H\}$ NMR (101 MHz, $CDCl_3$):

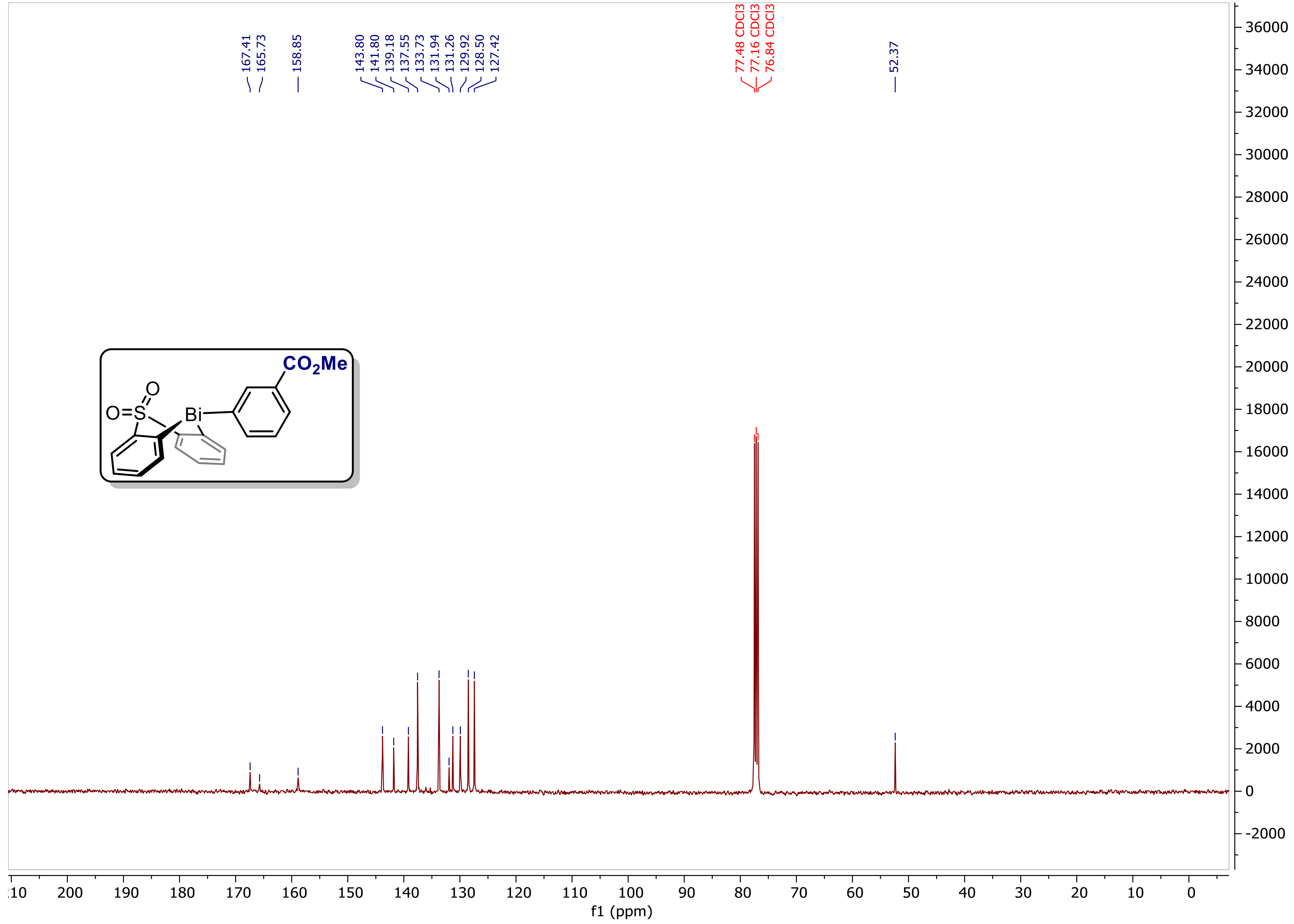

3 - ^{1}H NMR (400 MHz, $CDCl_3$):

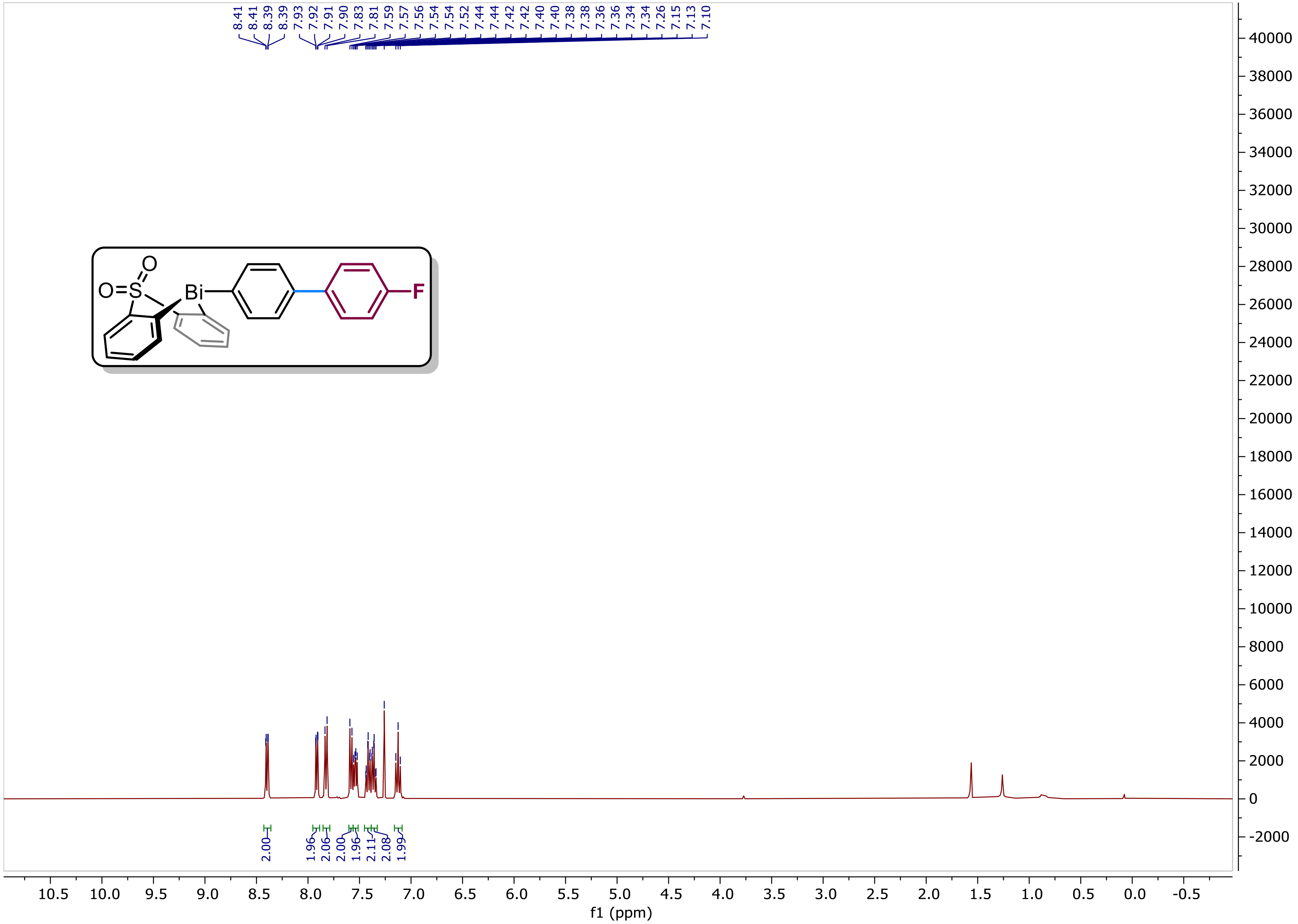

3 - $^{13}C\{^1H\}$ NMR (101 MHz, $CDCl_3$):

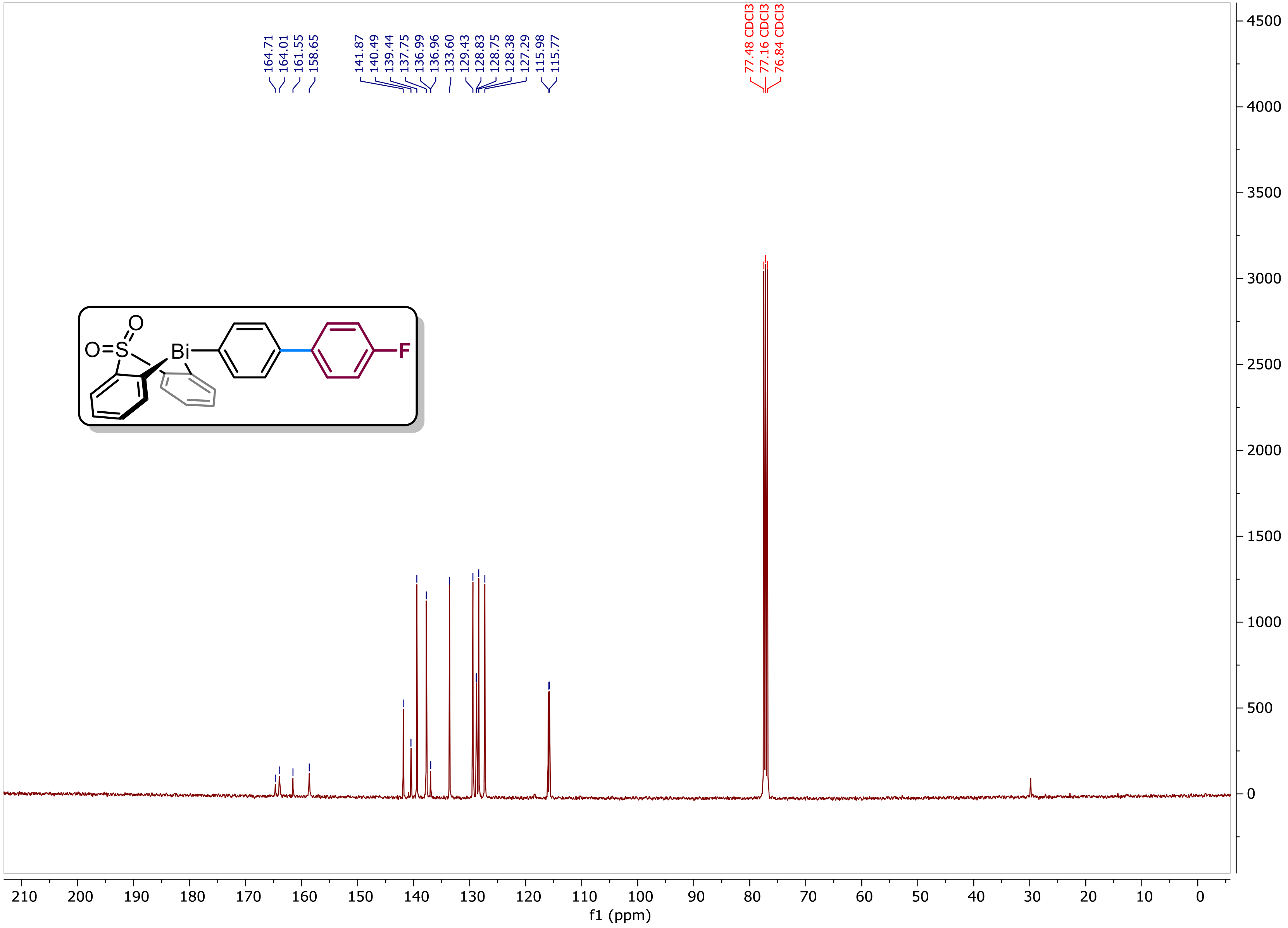

3 - ^{19}F NMR (376 MHz, $CDCl_3$):

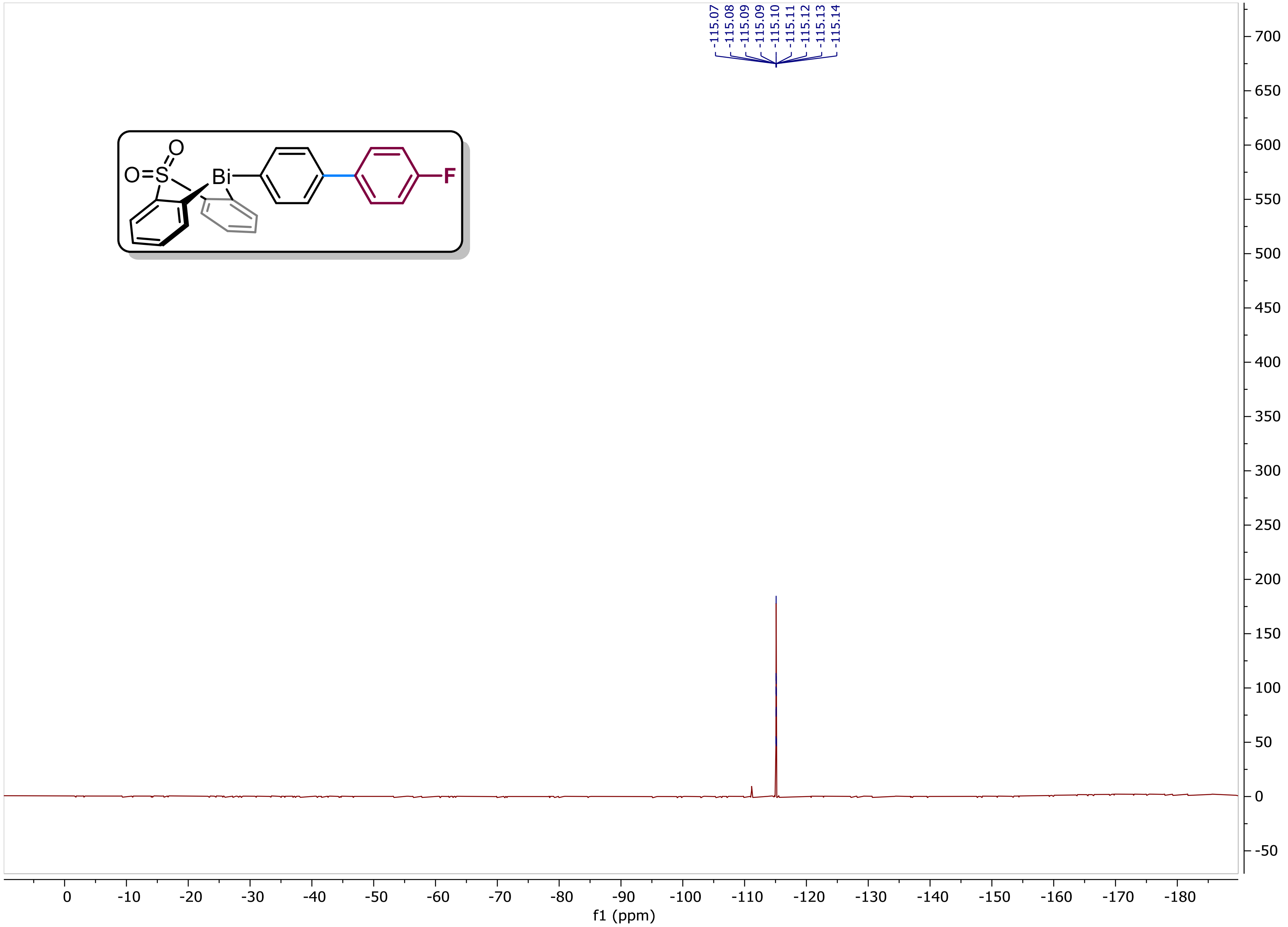

4 - ^{1}H NMR (400 MHz, CDCl$_3$):

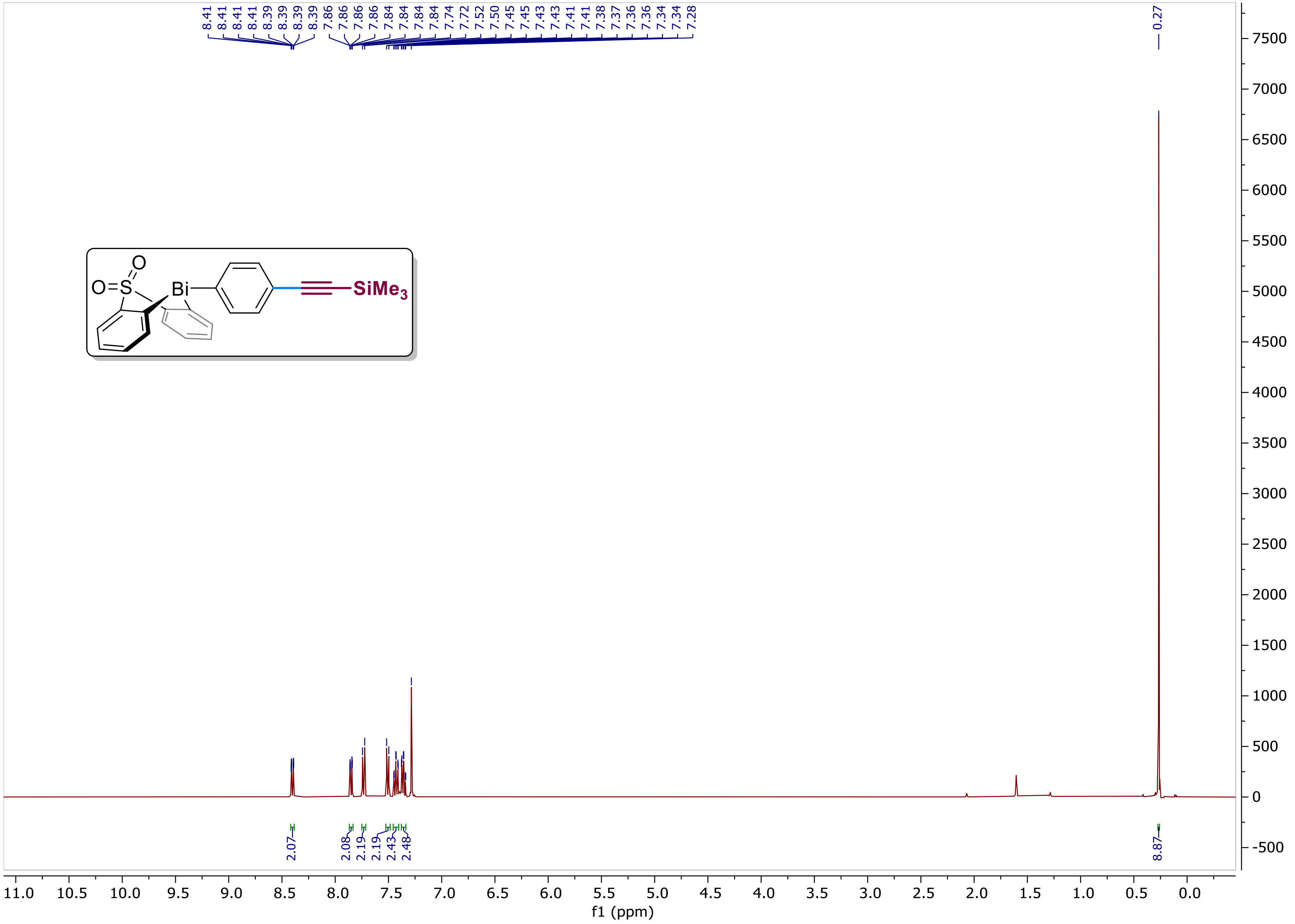

4 - $^{13}C\{^{1}H\}$ NMR (101 MHz, CDCl$_3$):

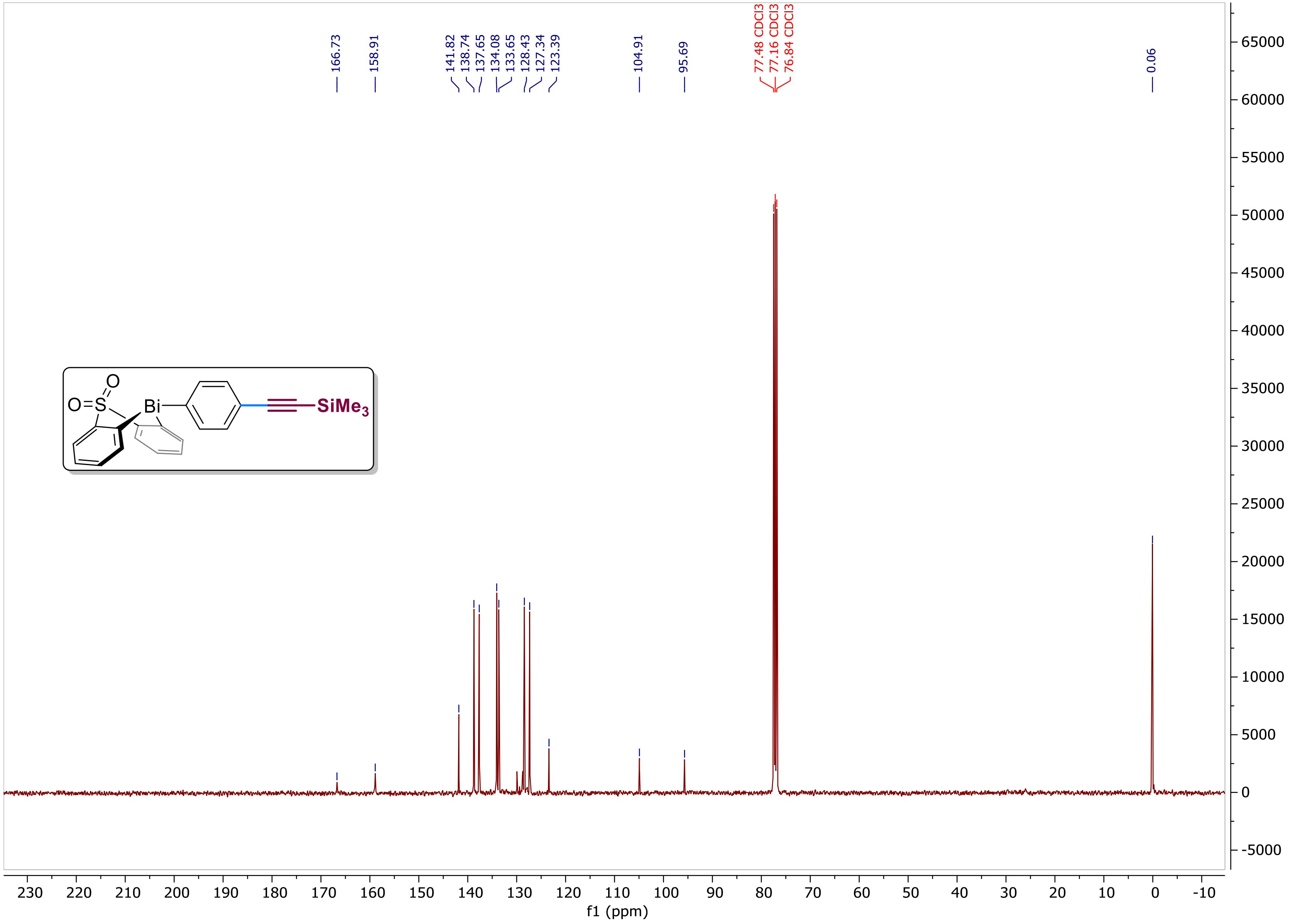

5 - ^{1}H NMR (400 MHz, $CDCl_3$):

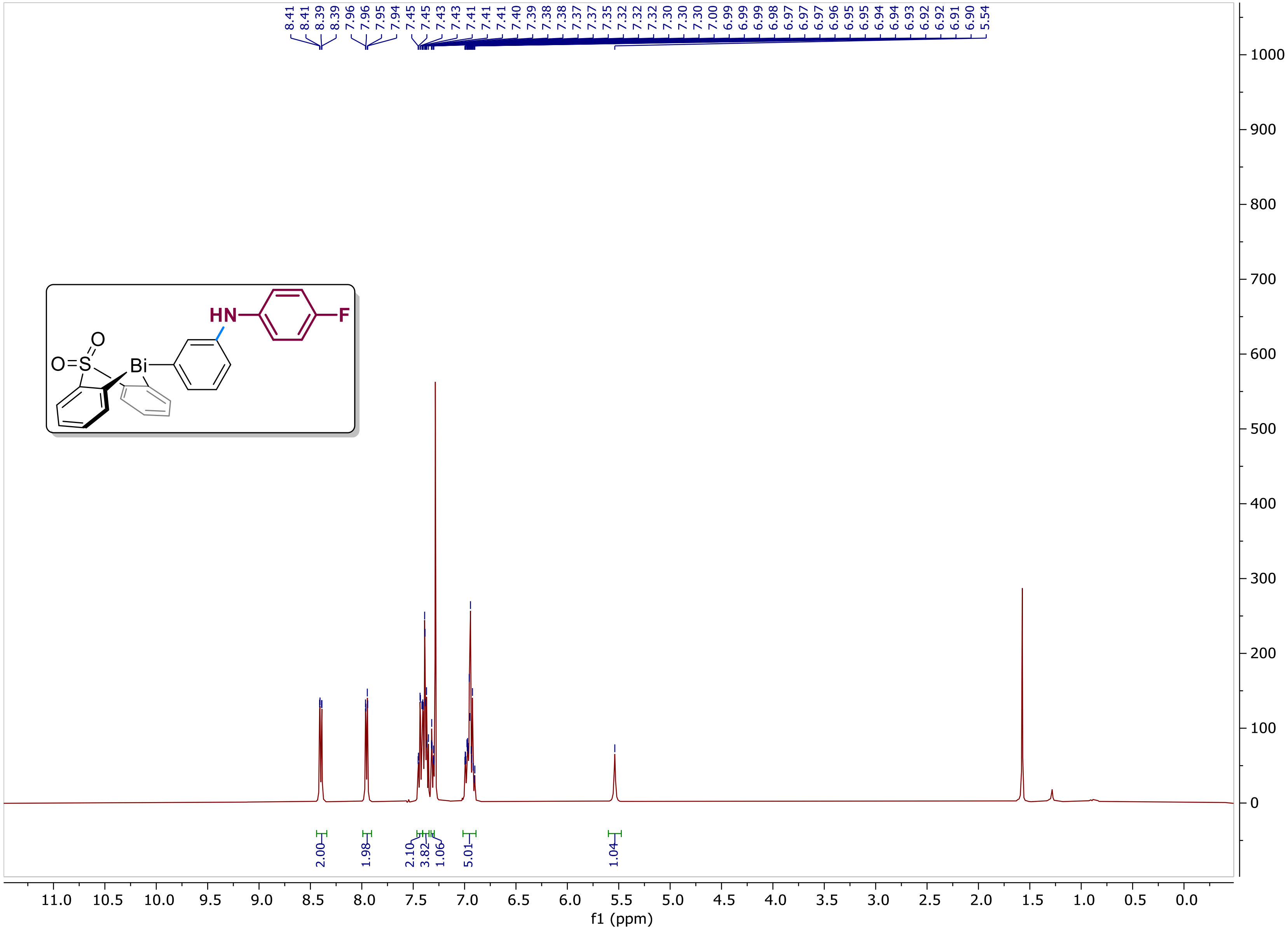

5 - $^{13}C\{^1H\}$ NMR (101 MHz, CDCl3):

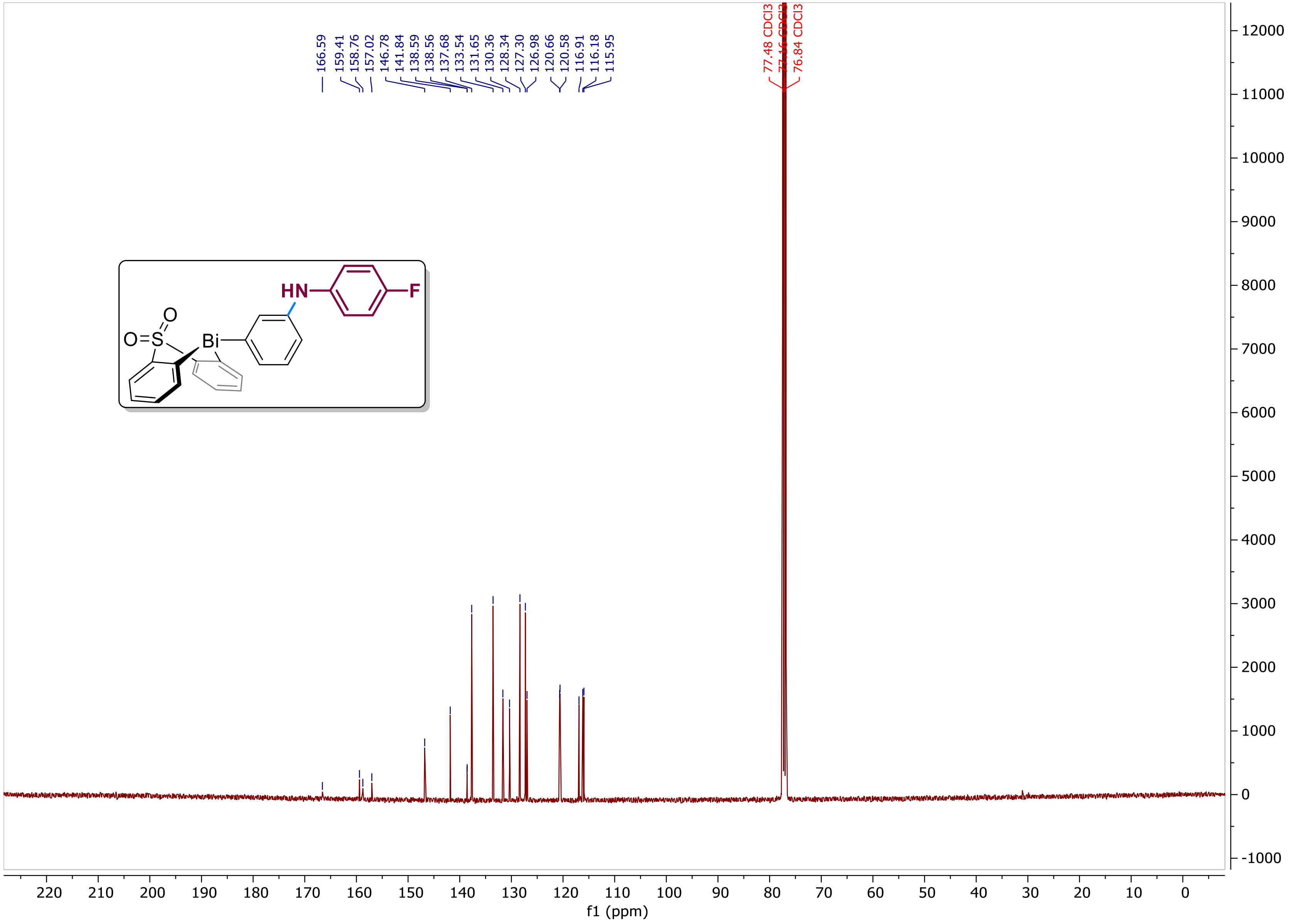

5 - $^{19}F\{^{1}H\}$ NMR (376 MHz, $CDCl_3$):

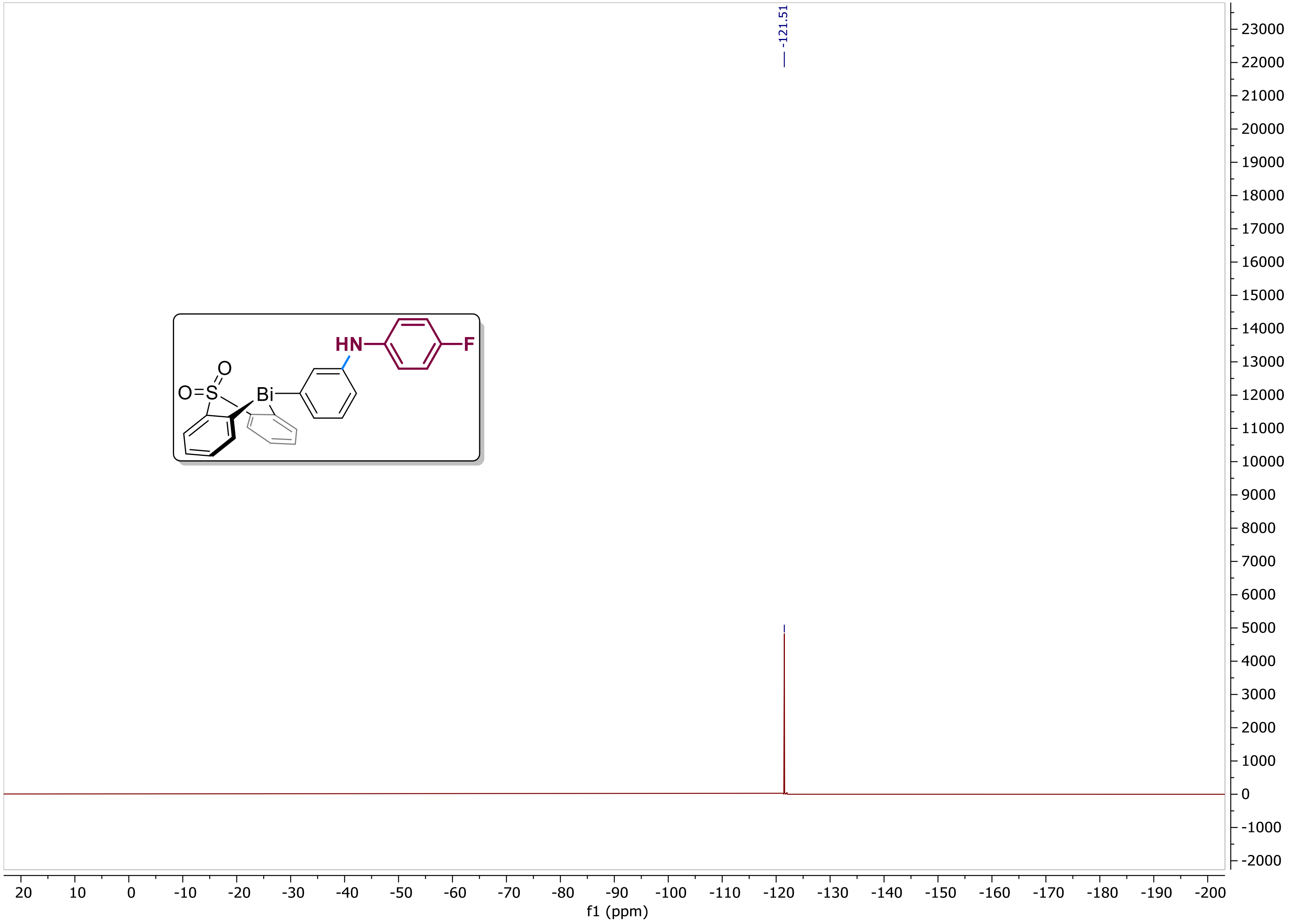

7 - ^{1}H NMR (400 MHz, $CDCl_3$):

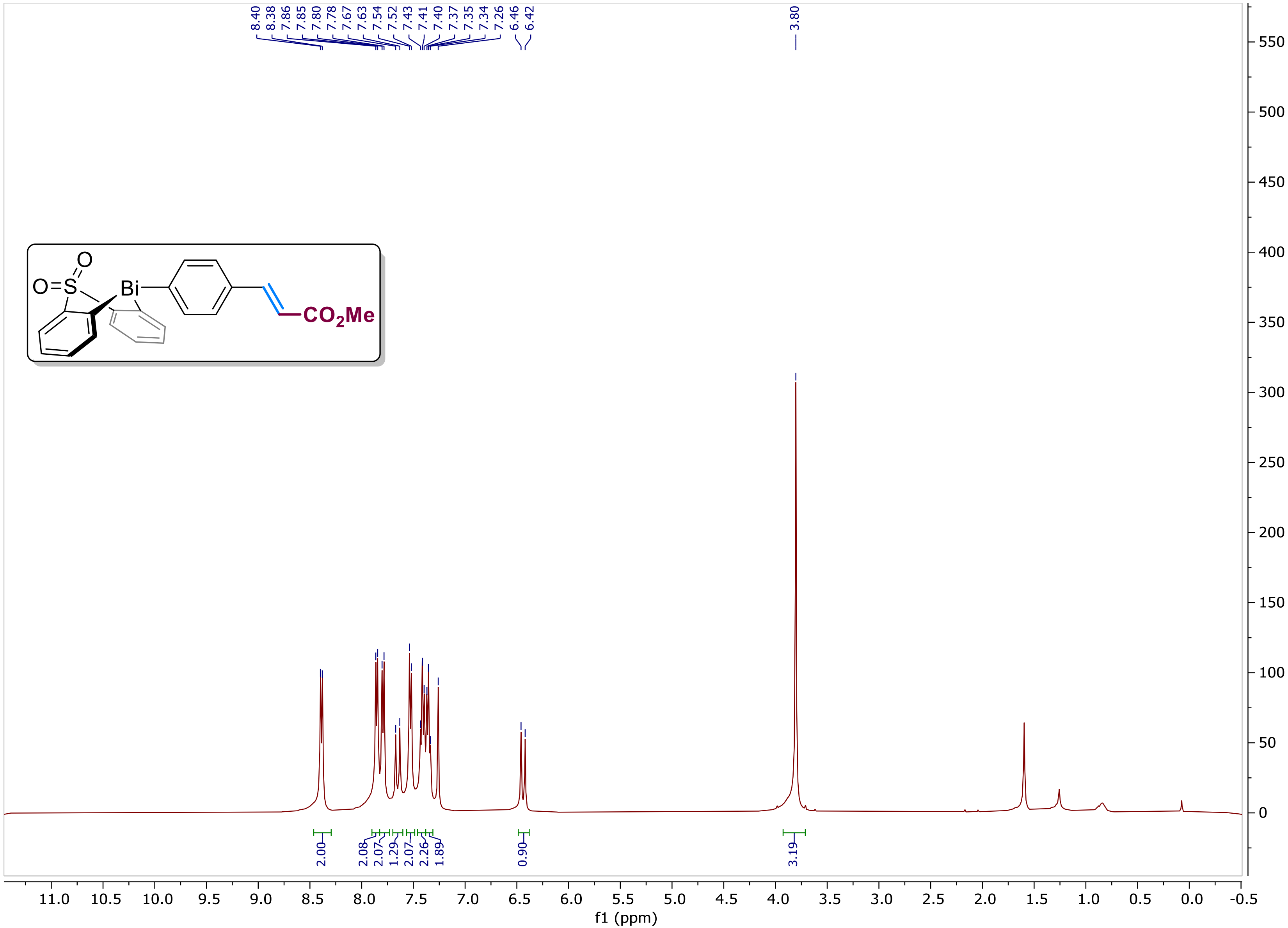

7 - $^{13}C\{^1H\}$ NMR (101 MHz, $CDCl_3$):

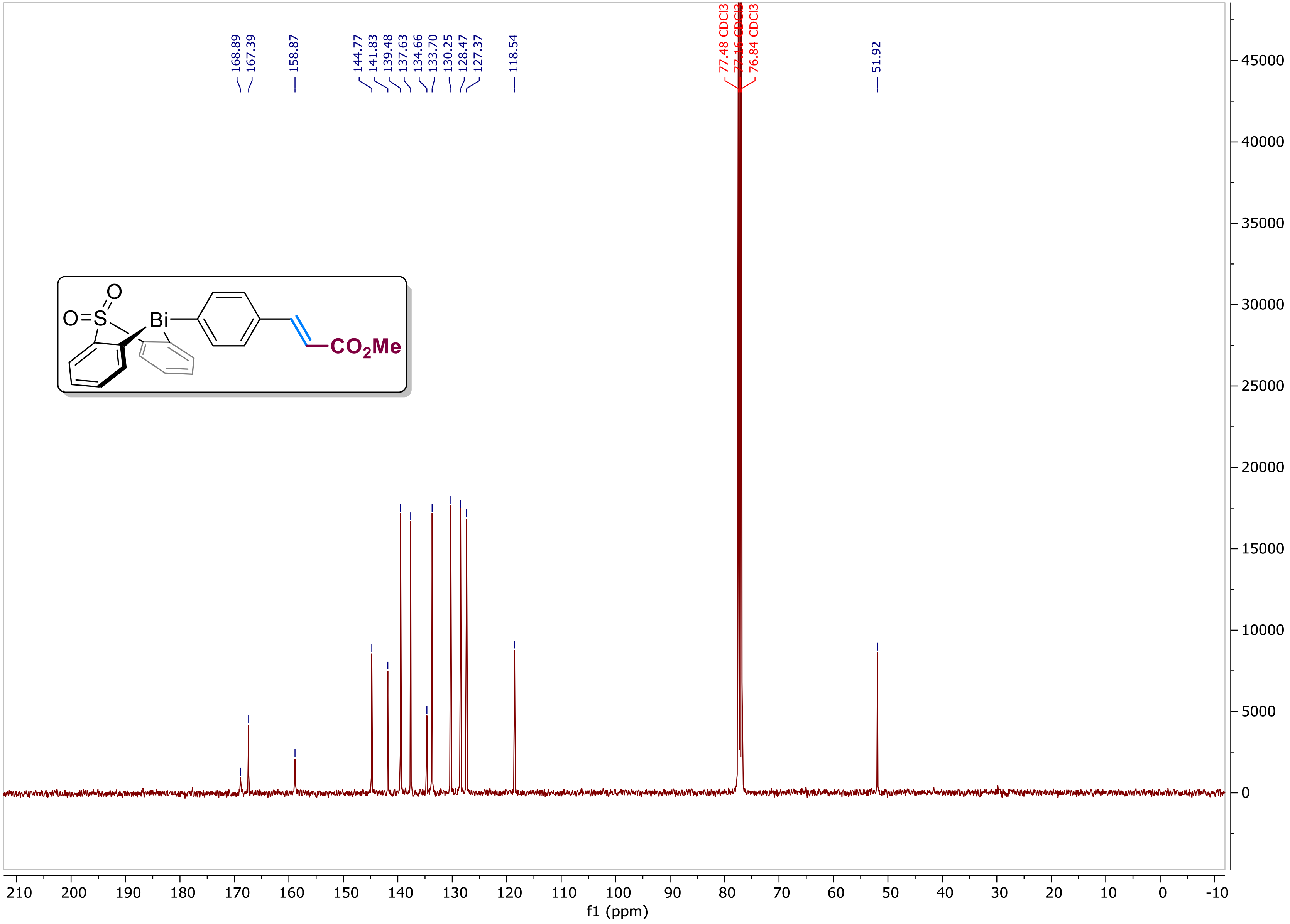

8 - ^{1}H NMR (400 MHz, CDCl$_3$):

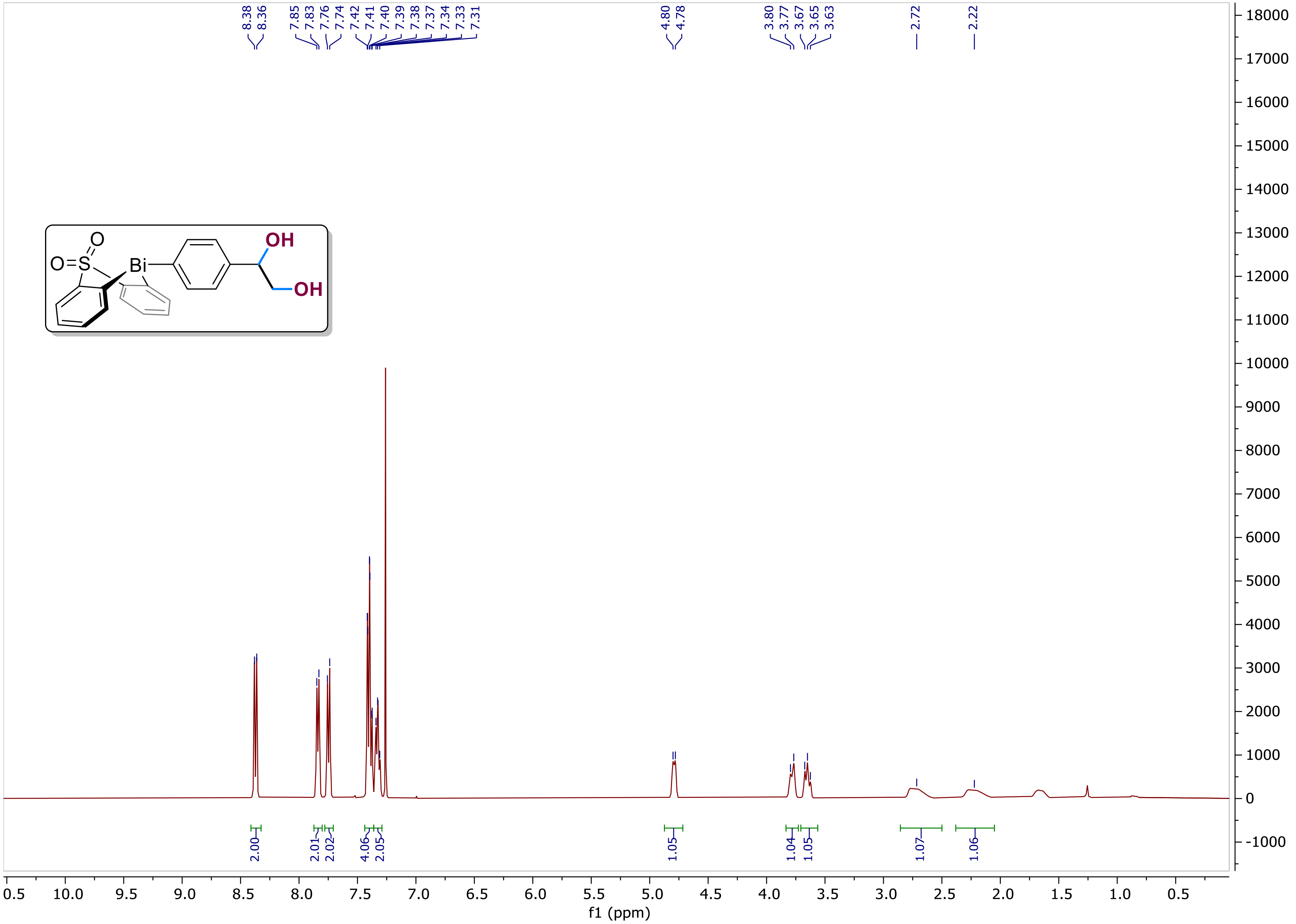

8 - $^{13}C\{^1H\}$ NMR (101 MHz, CDCl₃):

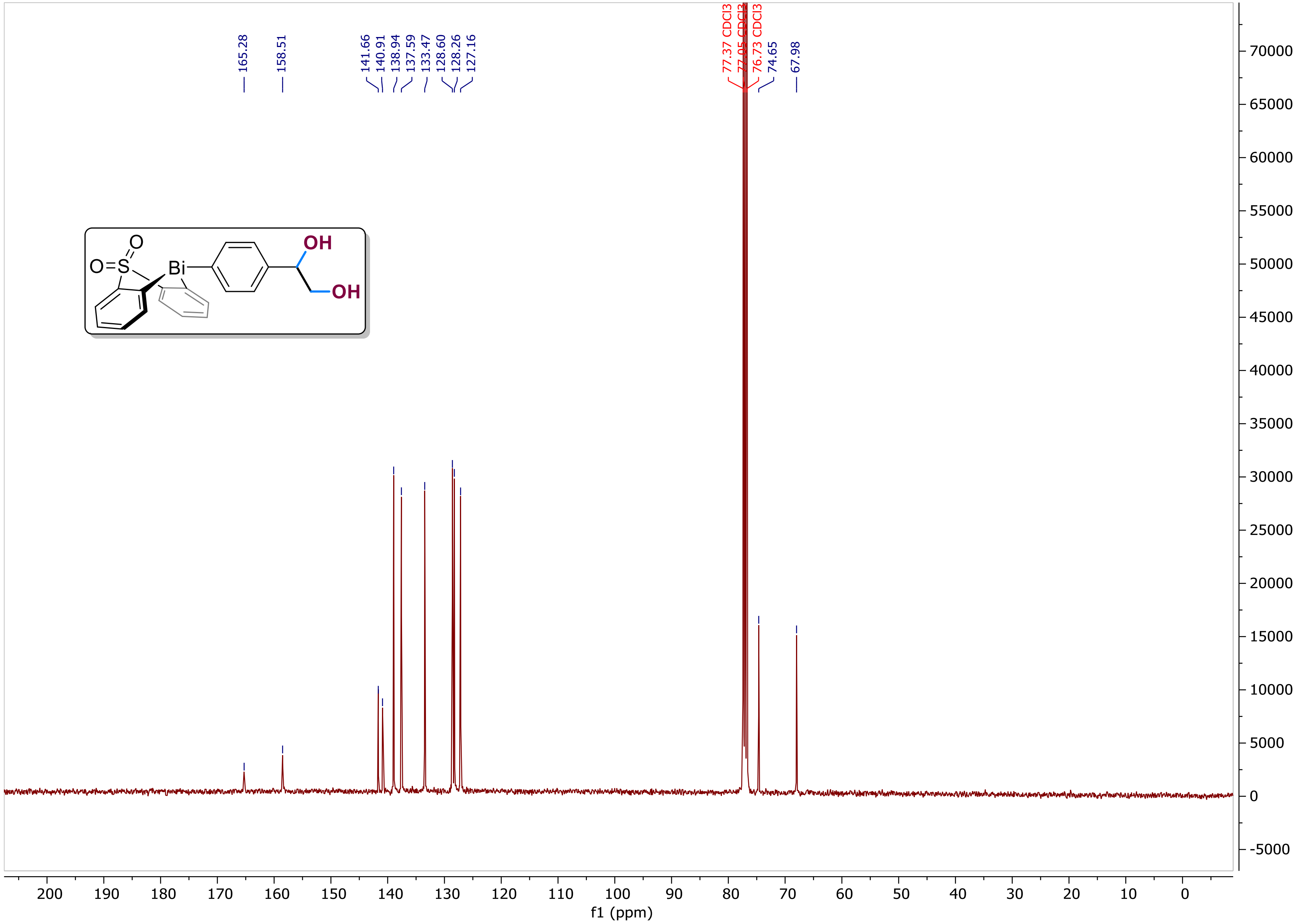

9 - ¹H NMR (400 MHz, $CDCl_3$):

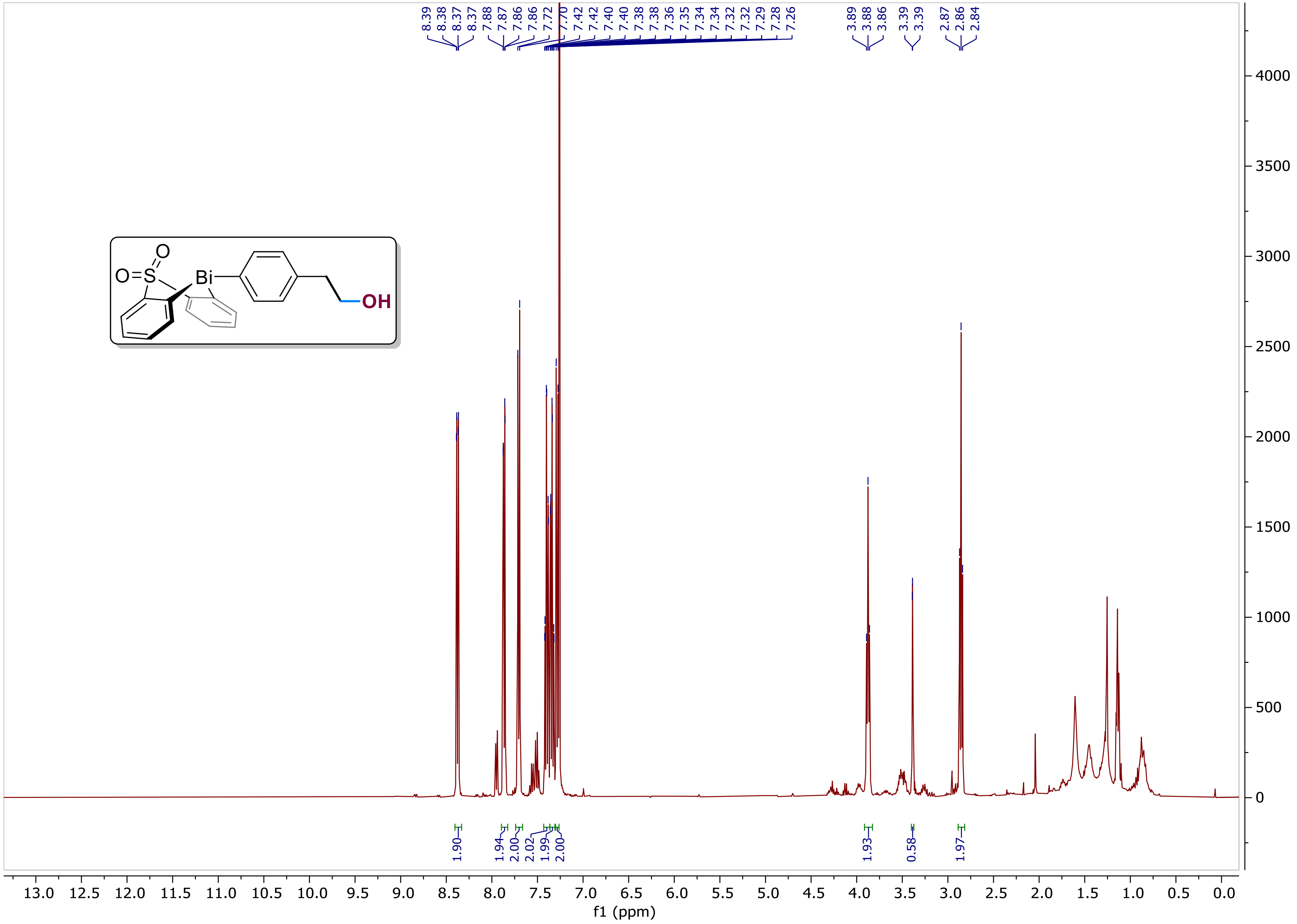

9 - $^{13}C\{^{1}H\}$ NMR (101 MHz, $CDCl_3$):

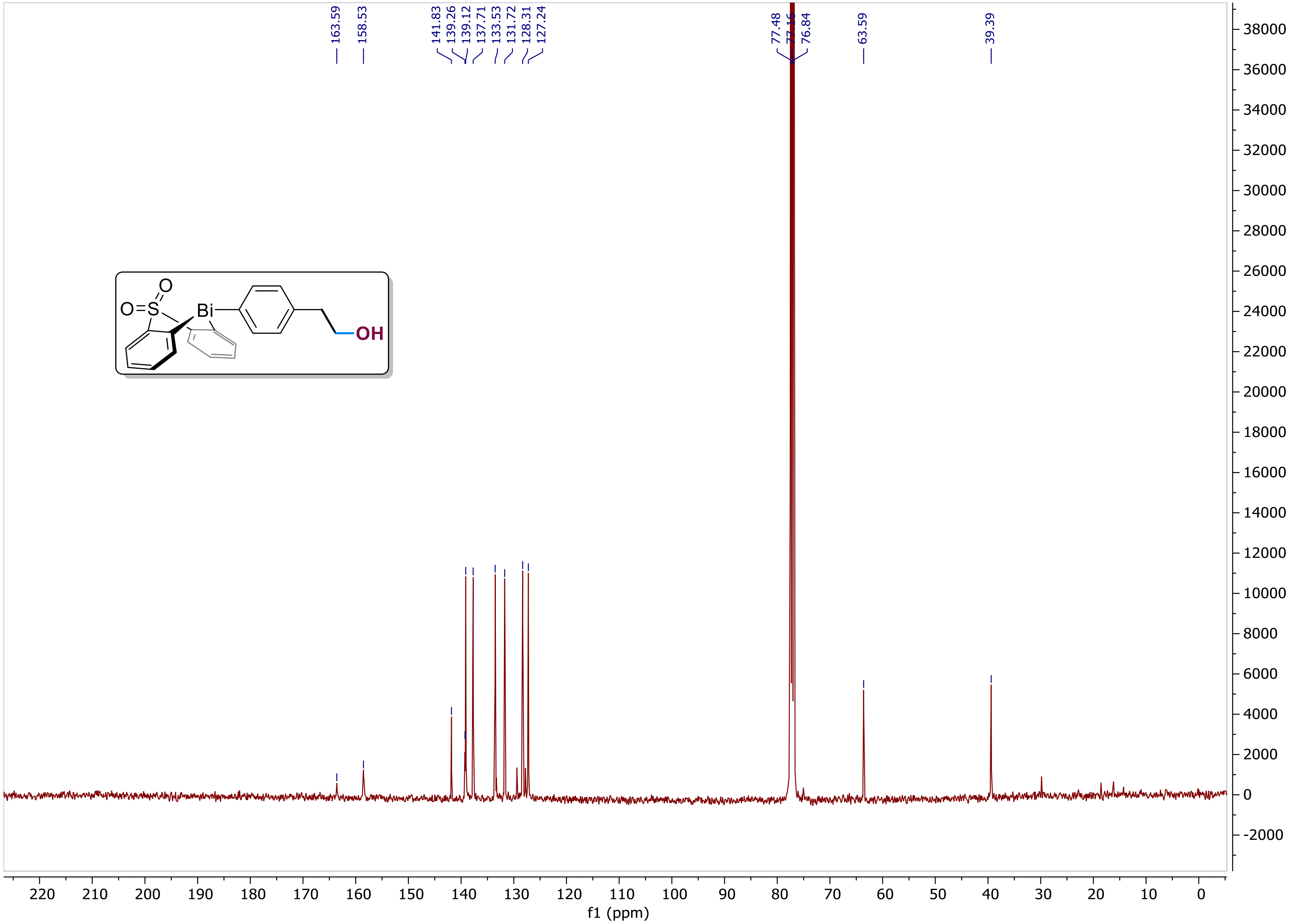

12 - ^{1}H NMR (400 MHz, CDCl$_3$):

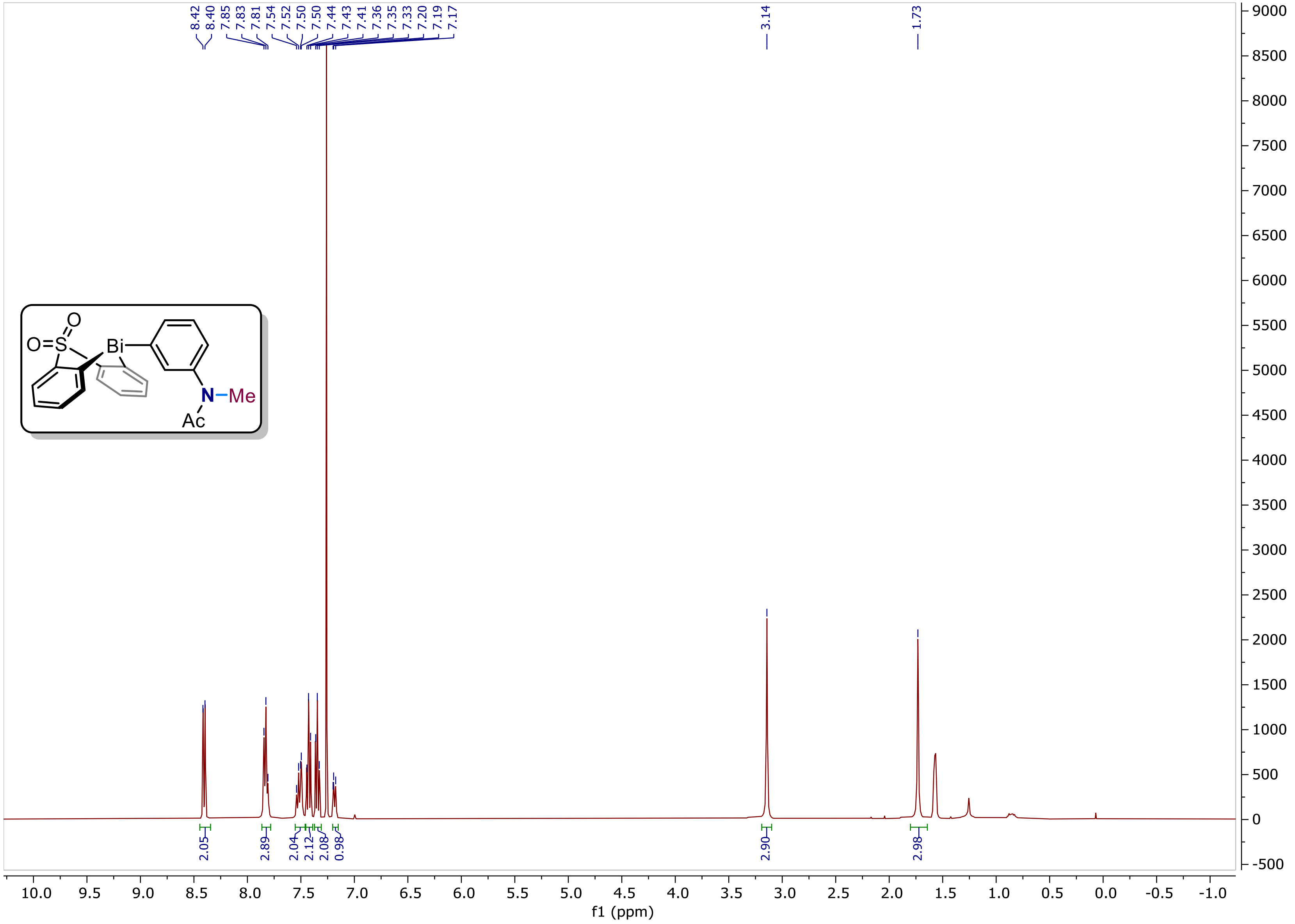

12 - $^{13}C\{^{1}H\}$ NMR (101 MHz, $CDCl_3$):

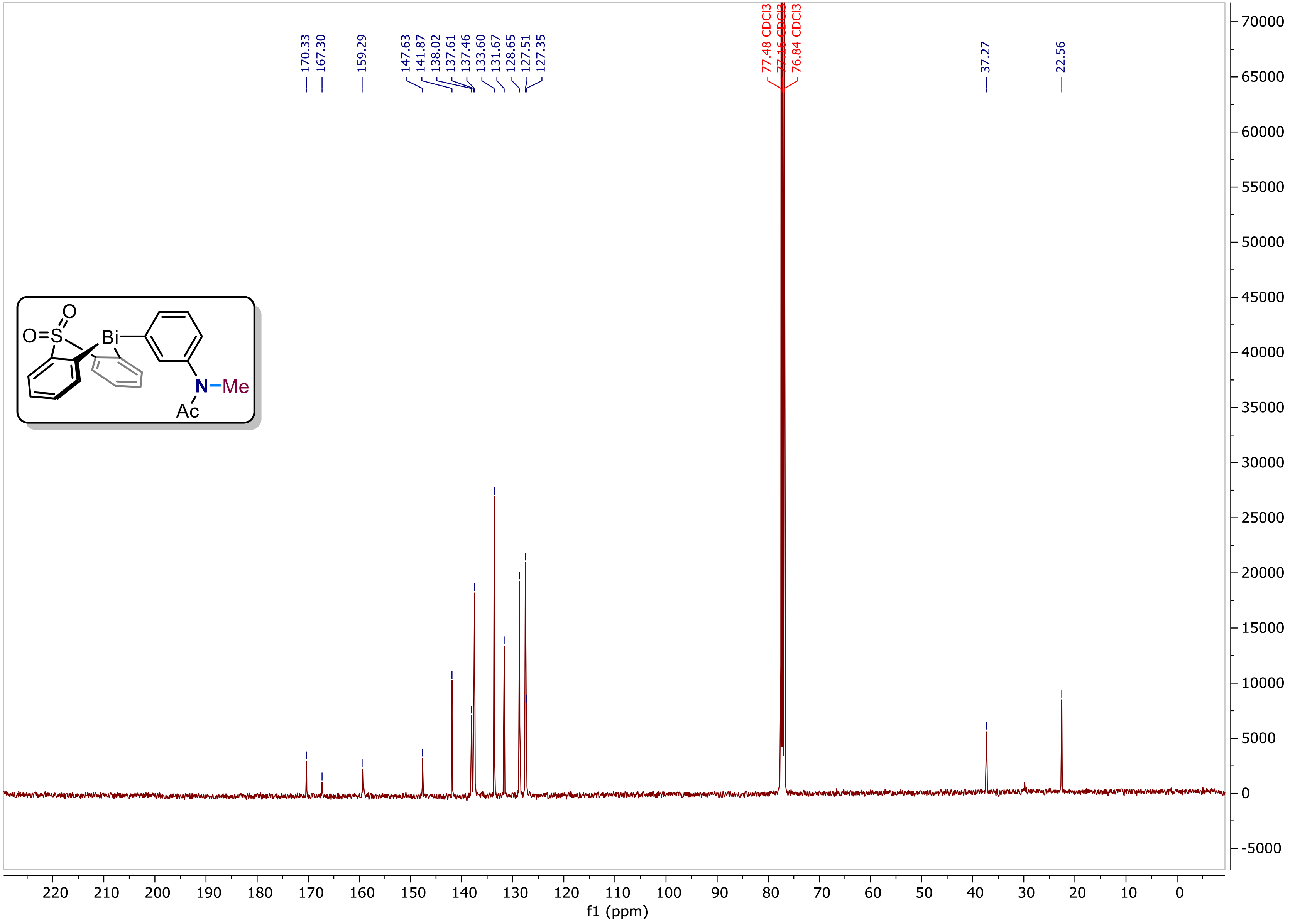

13 - ^{1}H NMR (400 MHz, CDCl3):

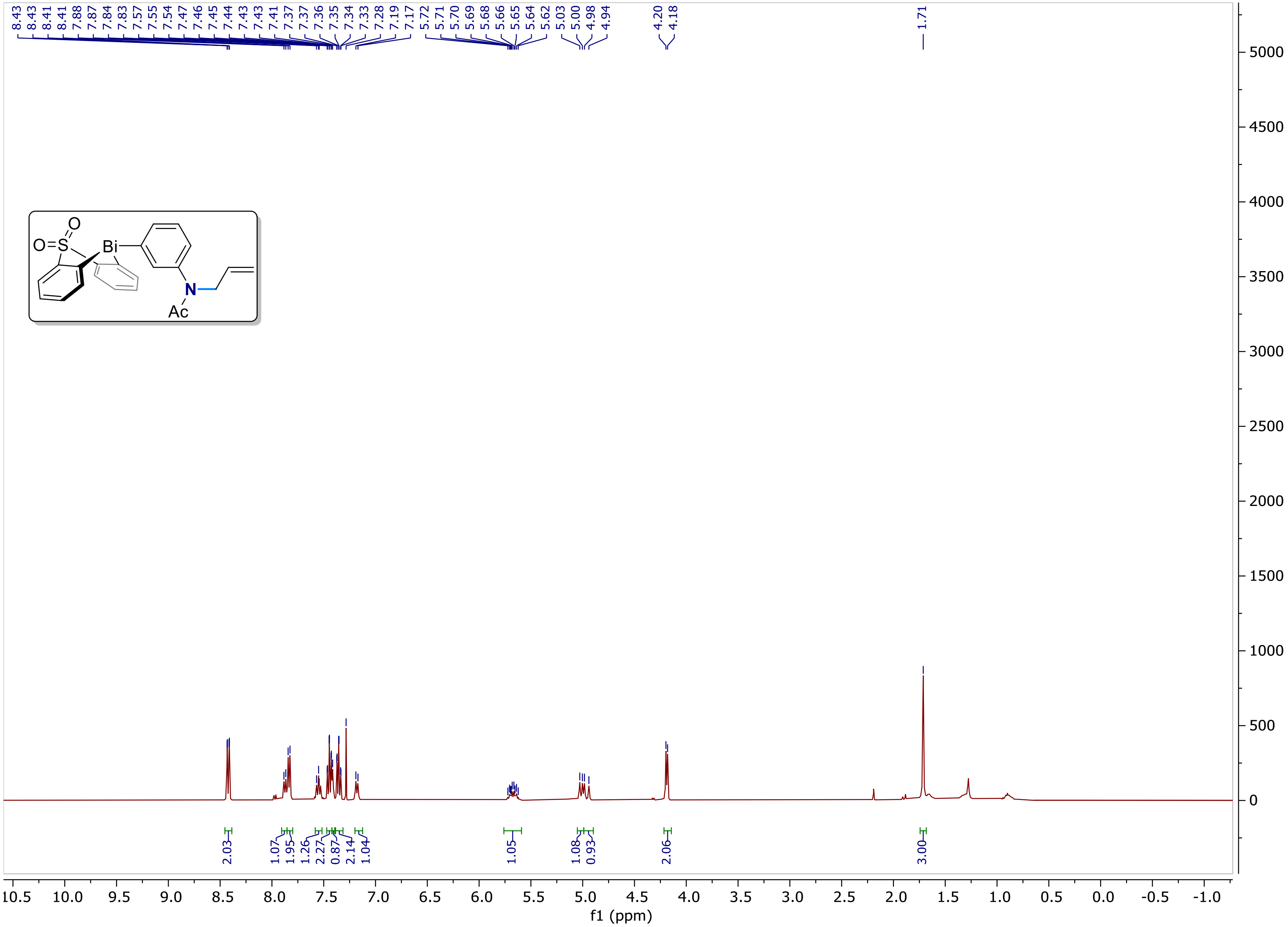

13 - $^{13}C\{^1H\}$ NMR (101 MHz, $CDCl_3$):

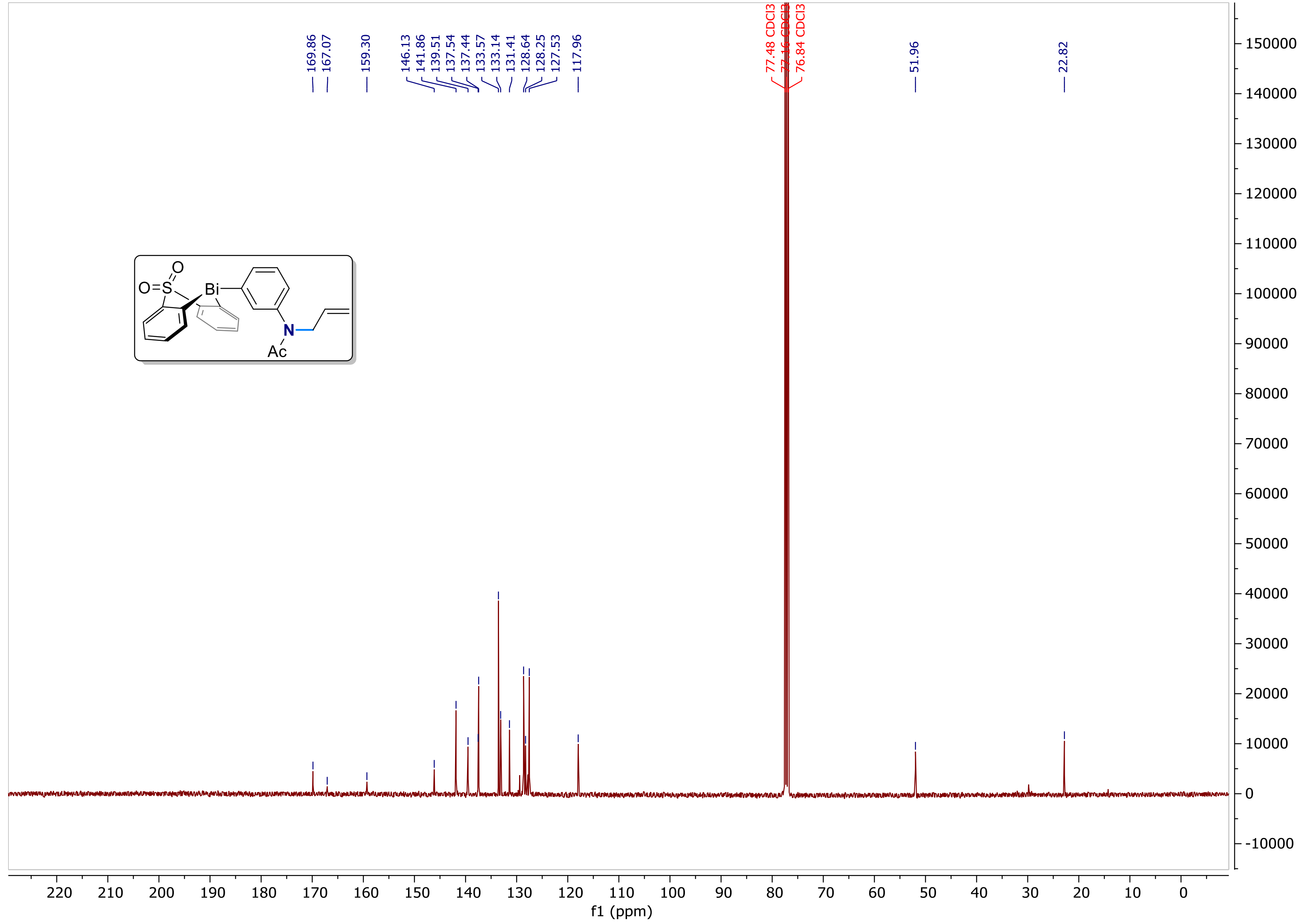

14 - ^{1}H NMR (400 MHz, DMSO-d_6):

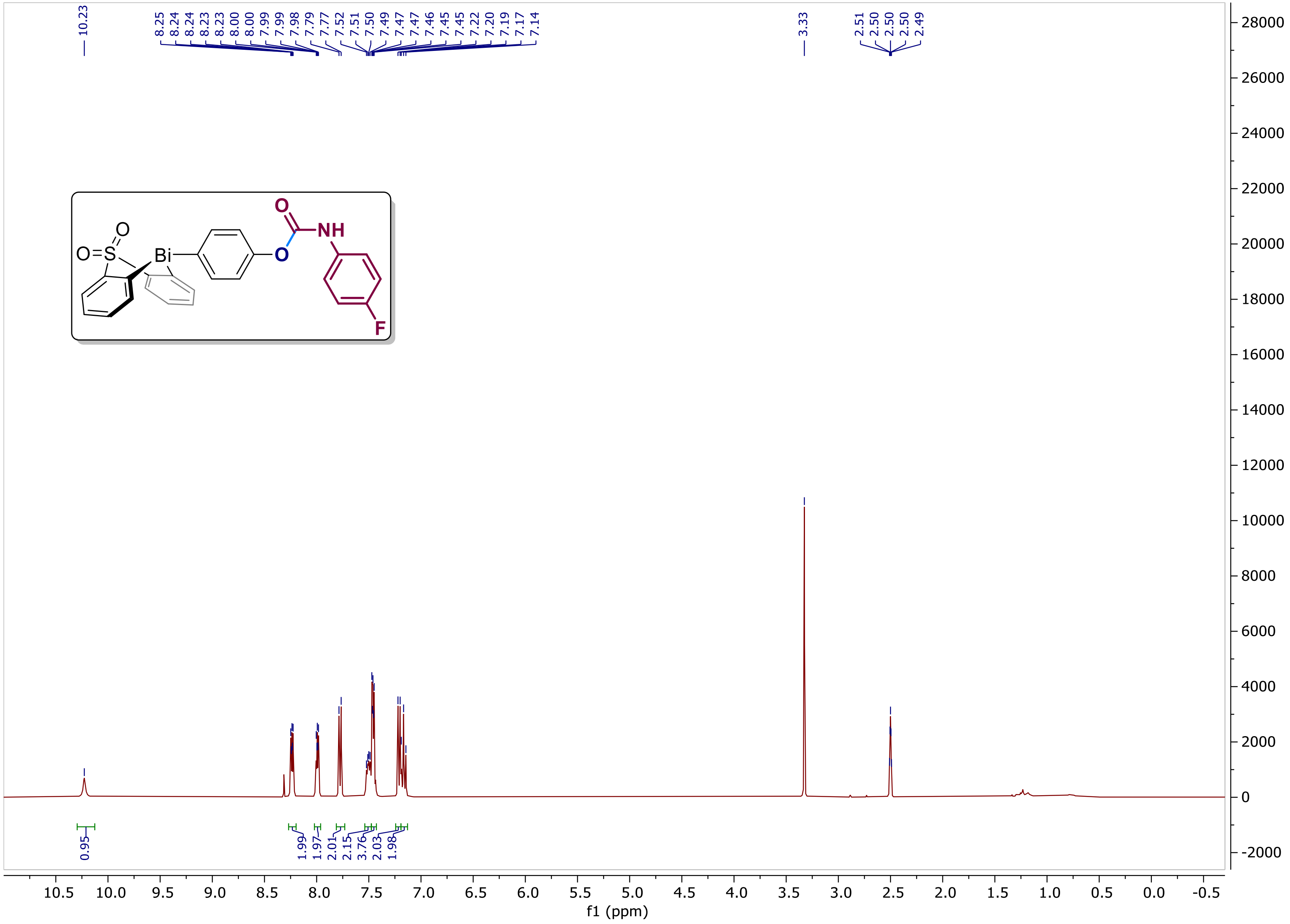

14 - $^{13}C\{^{1}H\}$ NMR (101 MHz, DMSO-d_6):

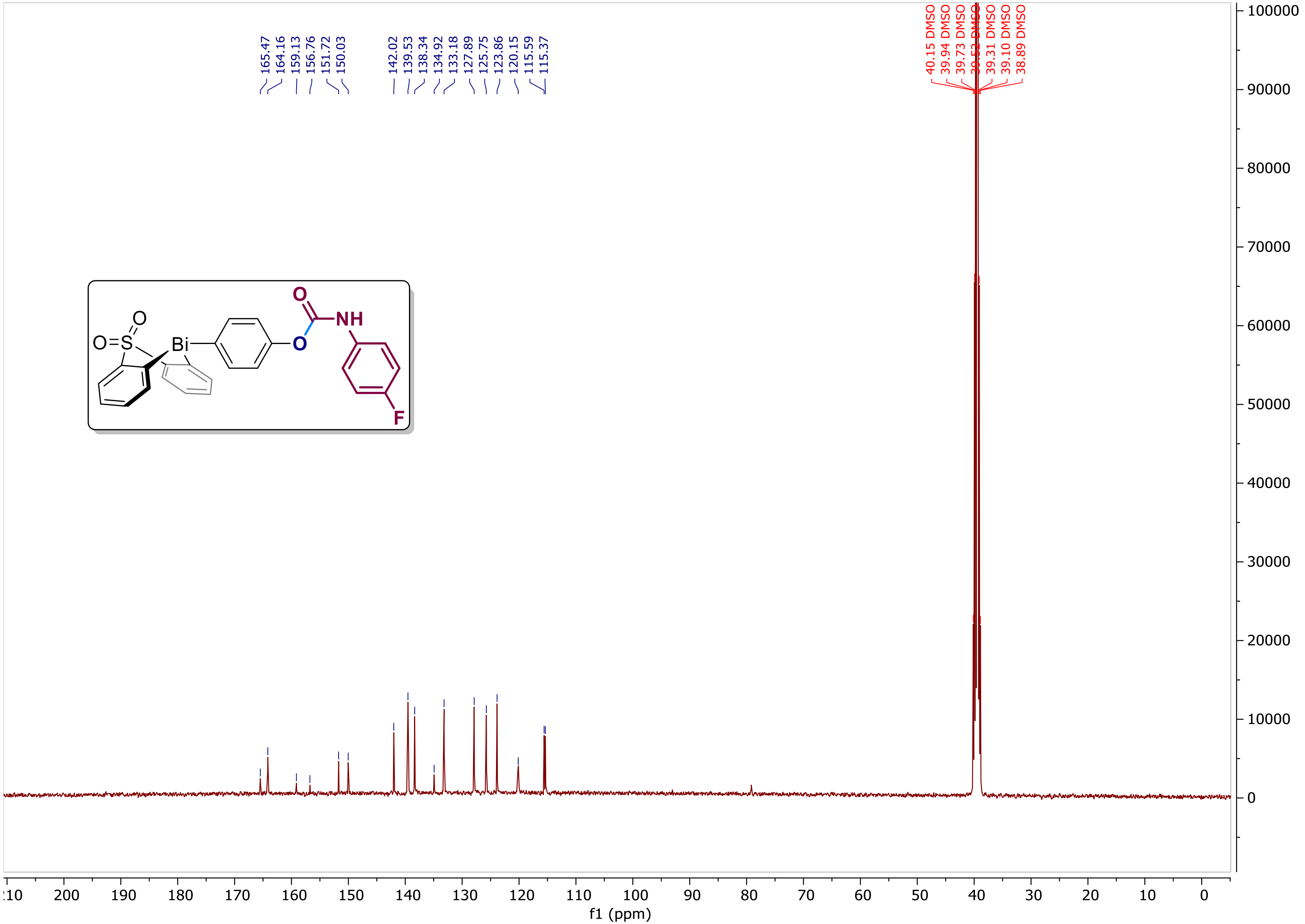

15 - ^{1}H NMR (400 MHz, $CDCl_3$):

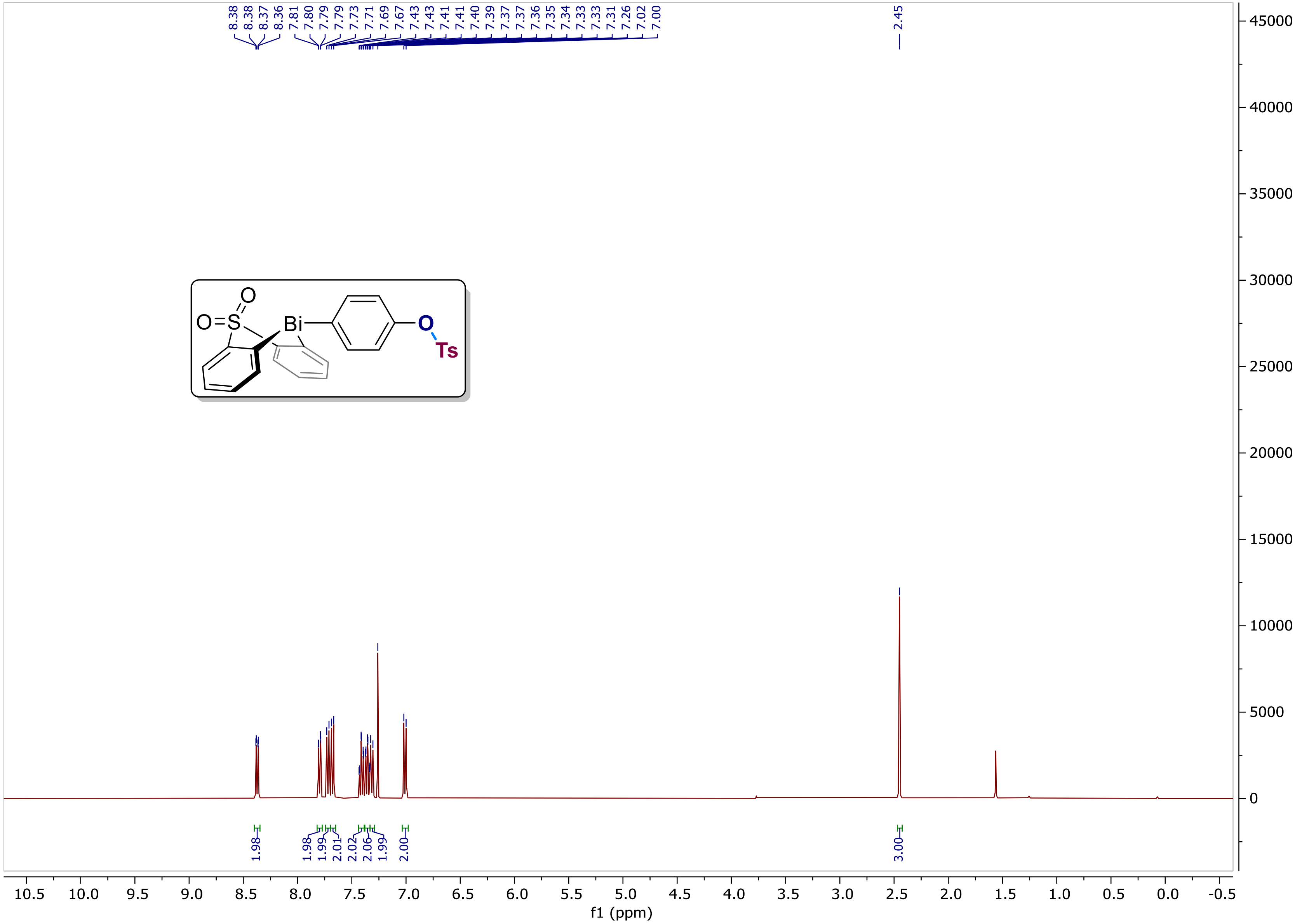

15 - $^{13}C\{^{1}H\}$ NMR (101 MHz, $CDCl_3$):

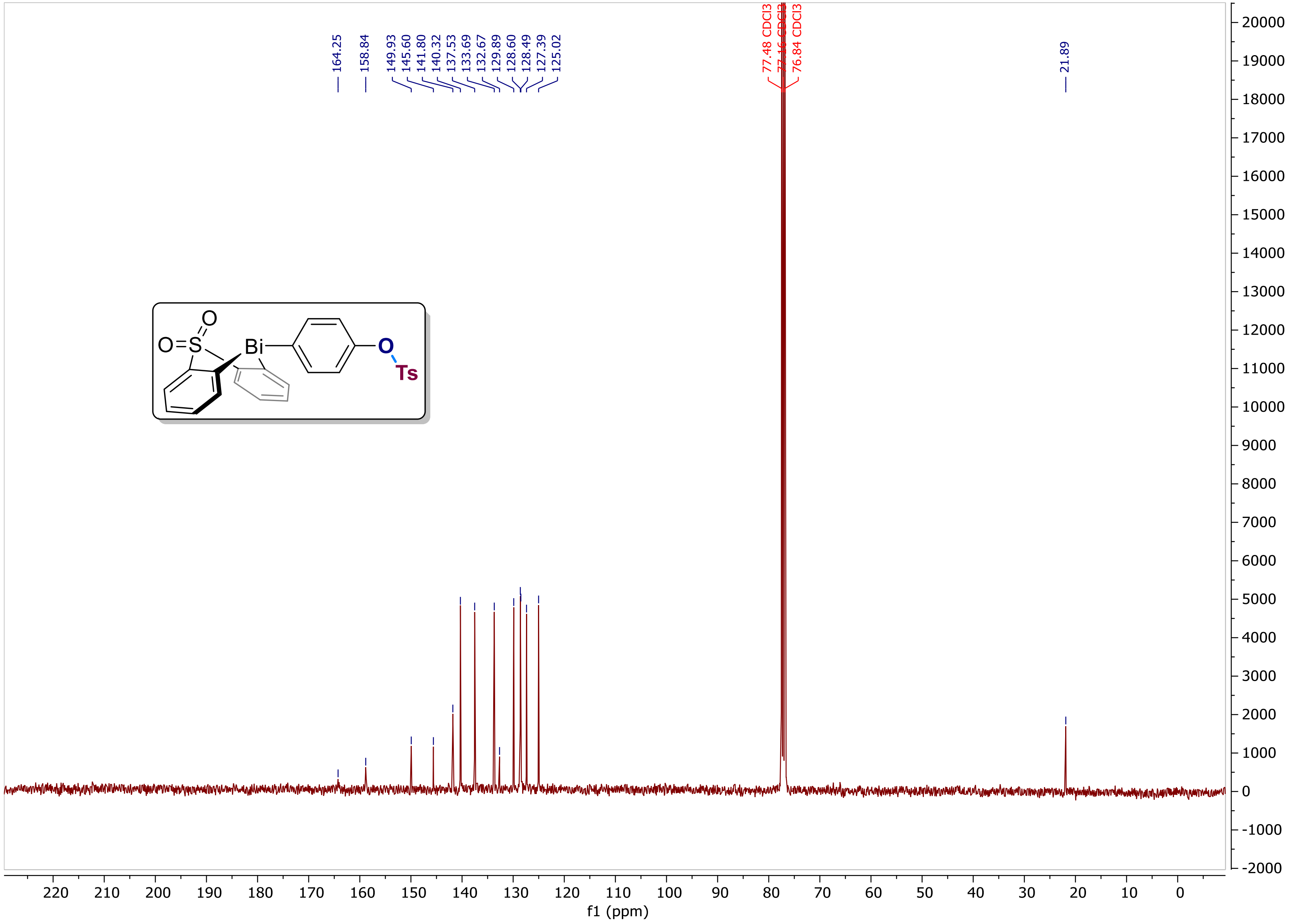

16 - ^{1}H NMR (400 MHz, $CDCl_3$):

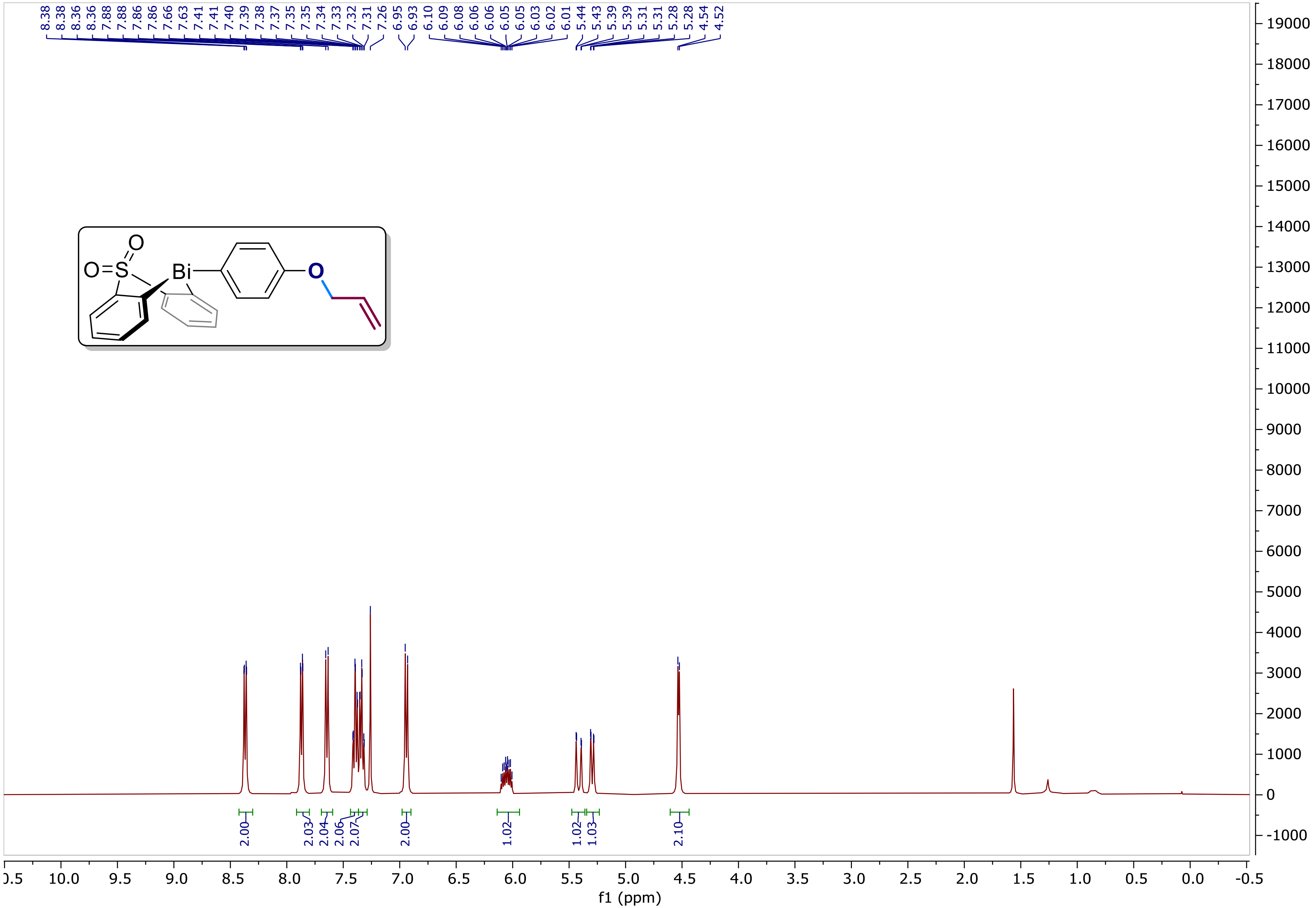

16 - $^{13}C\{^1H\}$ NMR (101 MHz, $CDCl_3$):

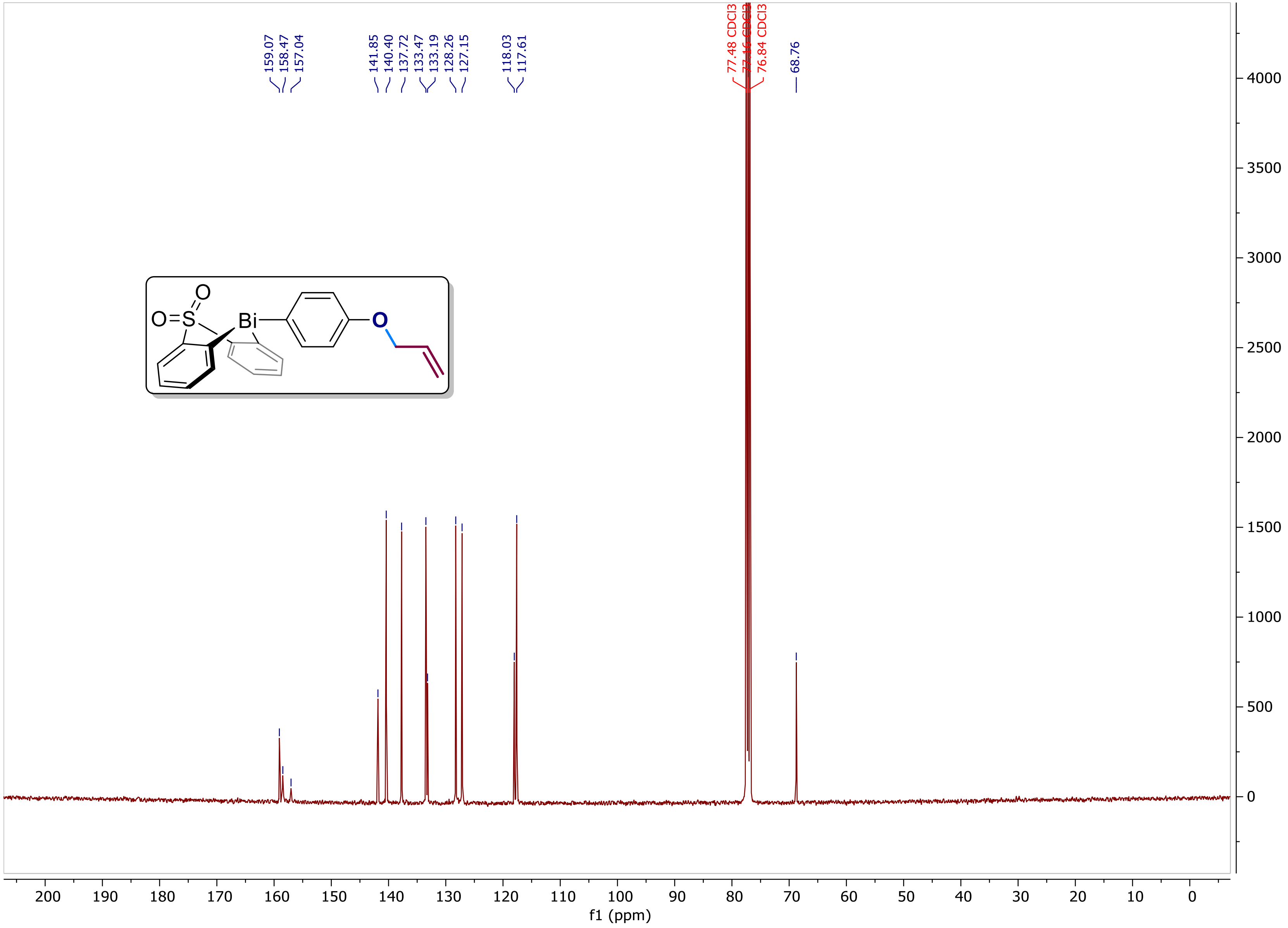

17 - ^{1}H NMR (400 MHz, CDCl3):

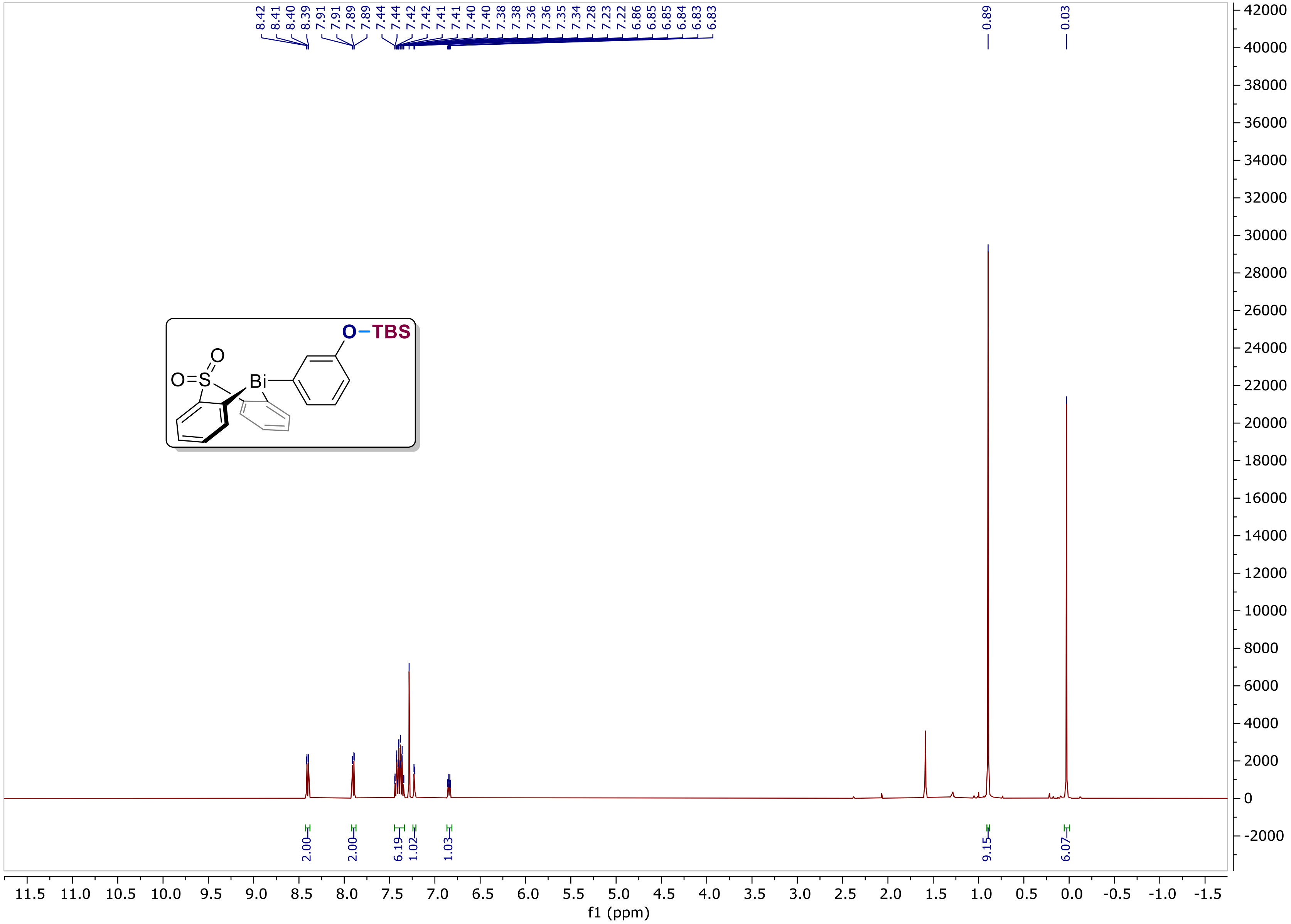

17 - $^{13}C\{^1H\}$ NMR (101 MHz, $CDCl_3$):

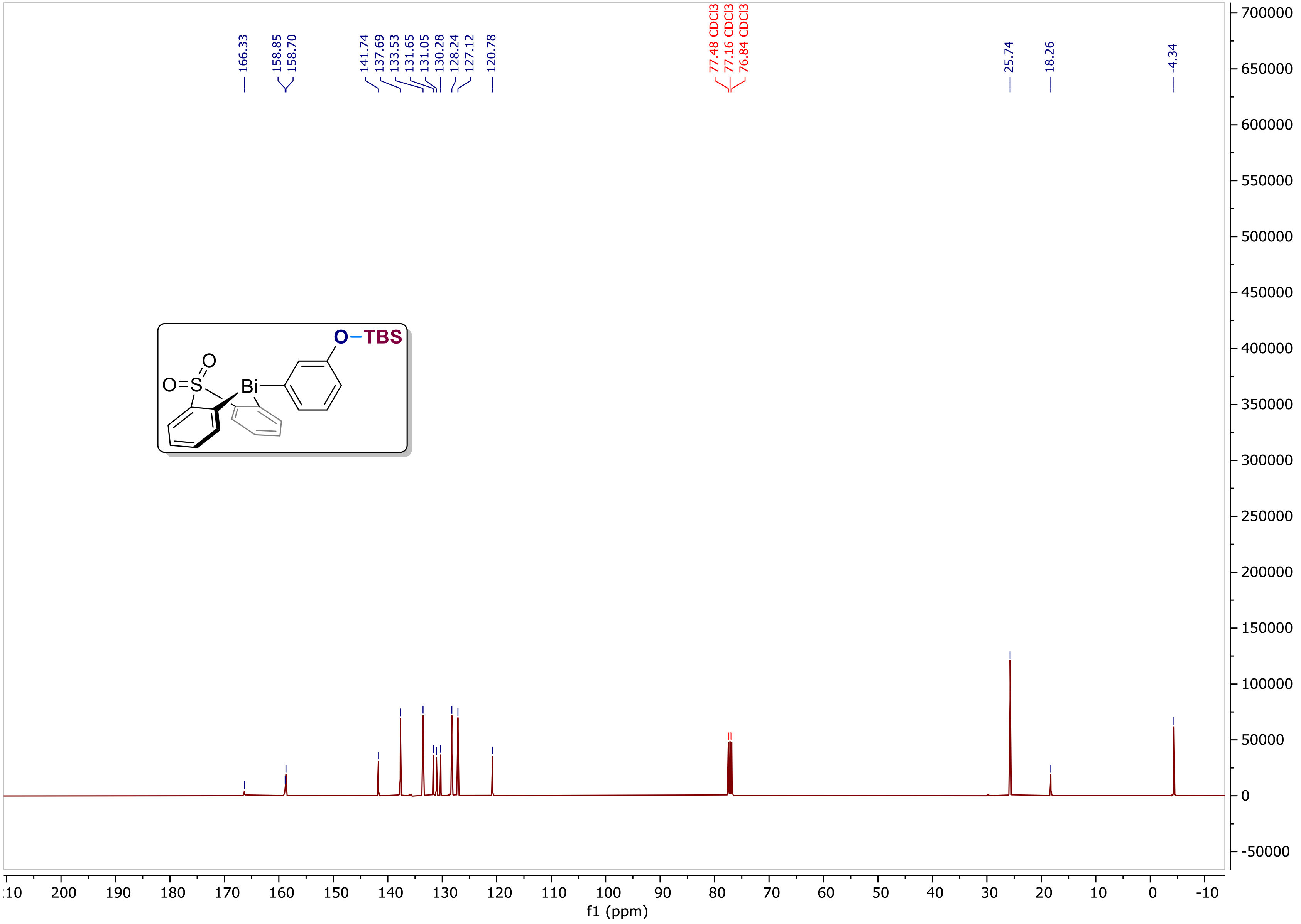

18 - ^{1}H NMR (400 MHz, DMSO-d_6):

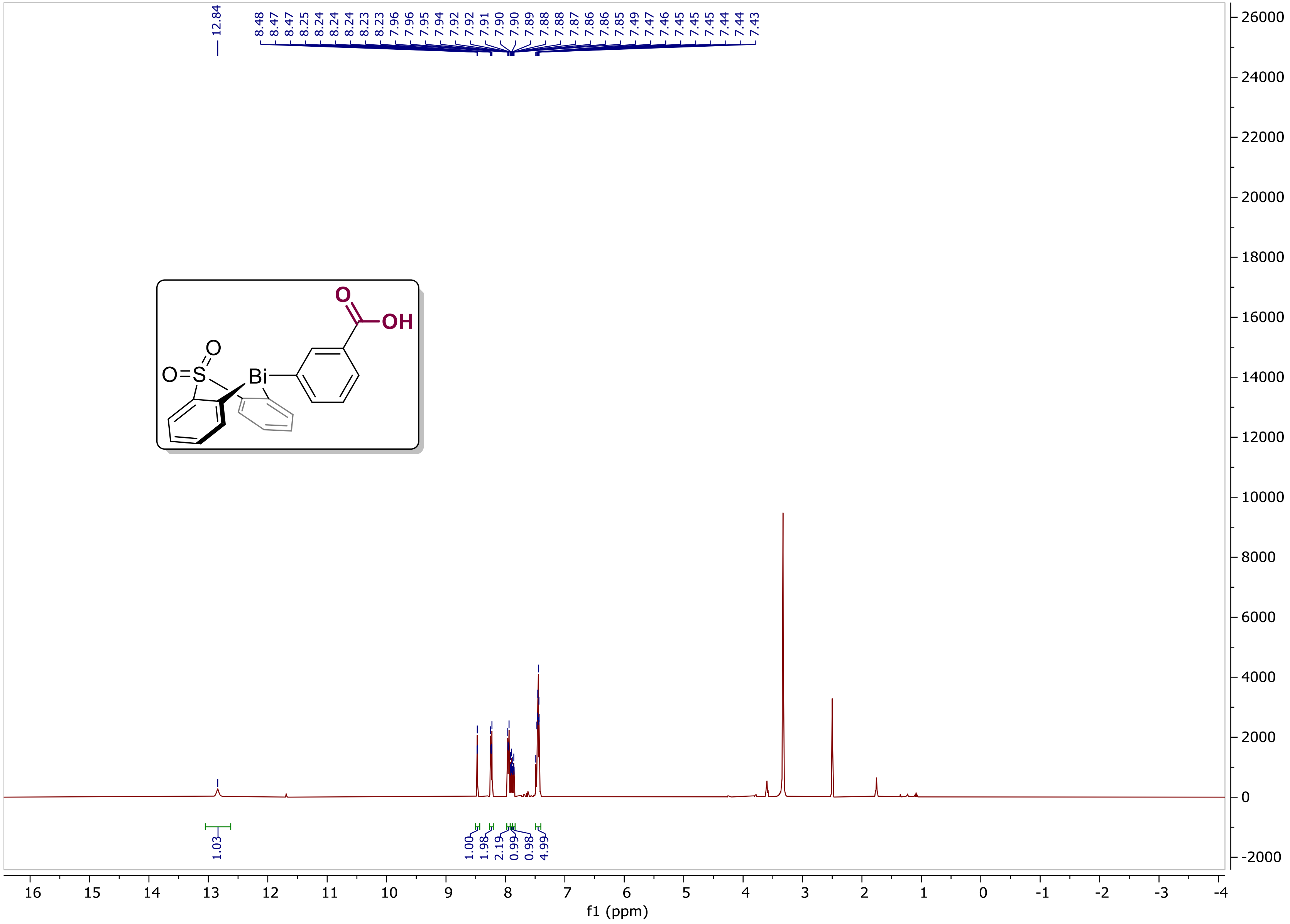

18 - $^{13}C\{^{1}H\}$ NMR (101 MHz, DMSO-d_6):

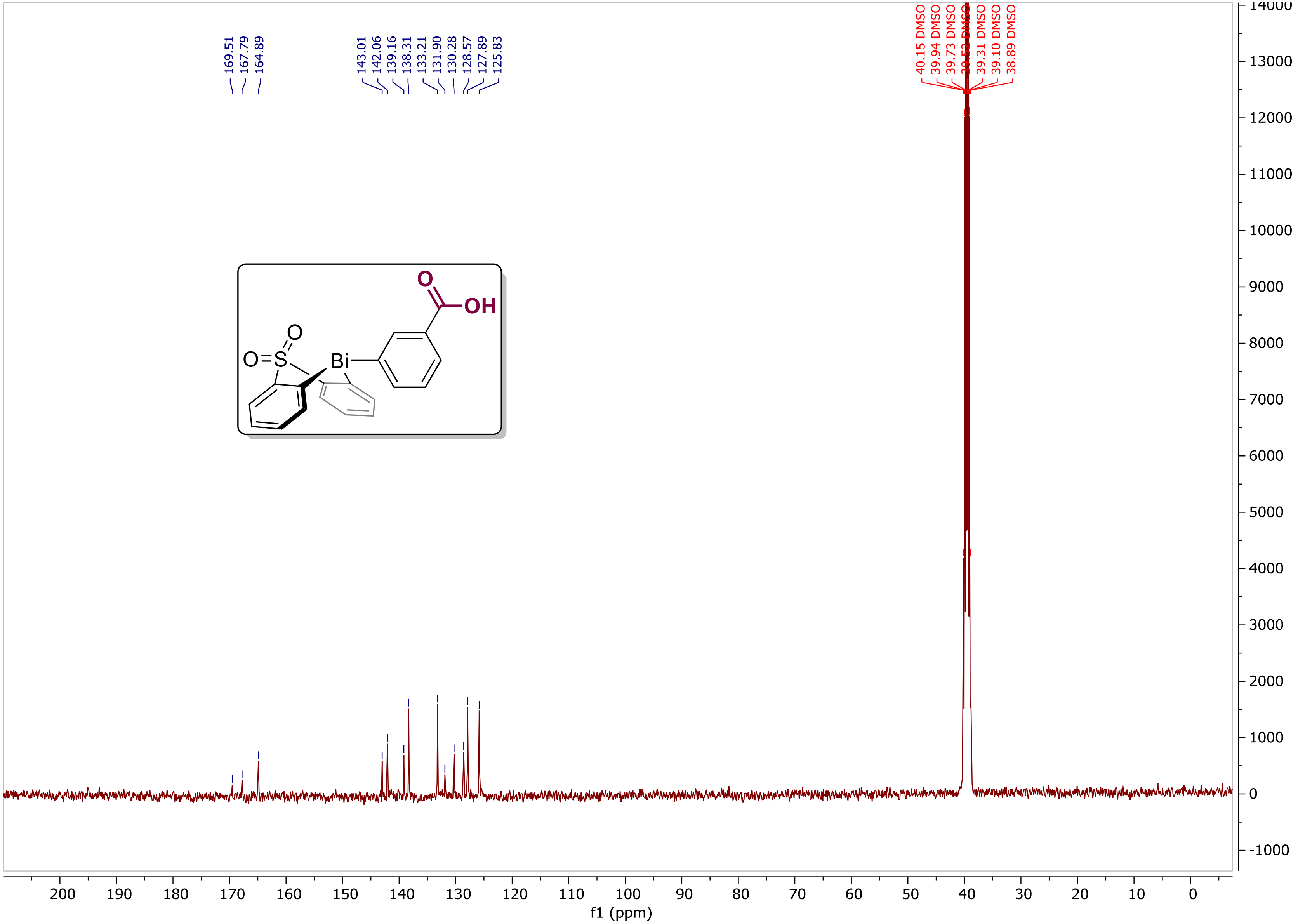

19 - ^{1}H NMR (400 MHz, $CDCl_3$):

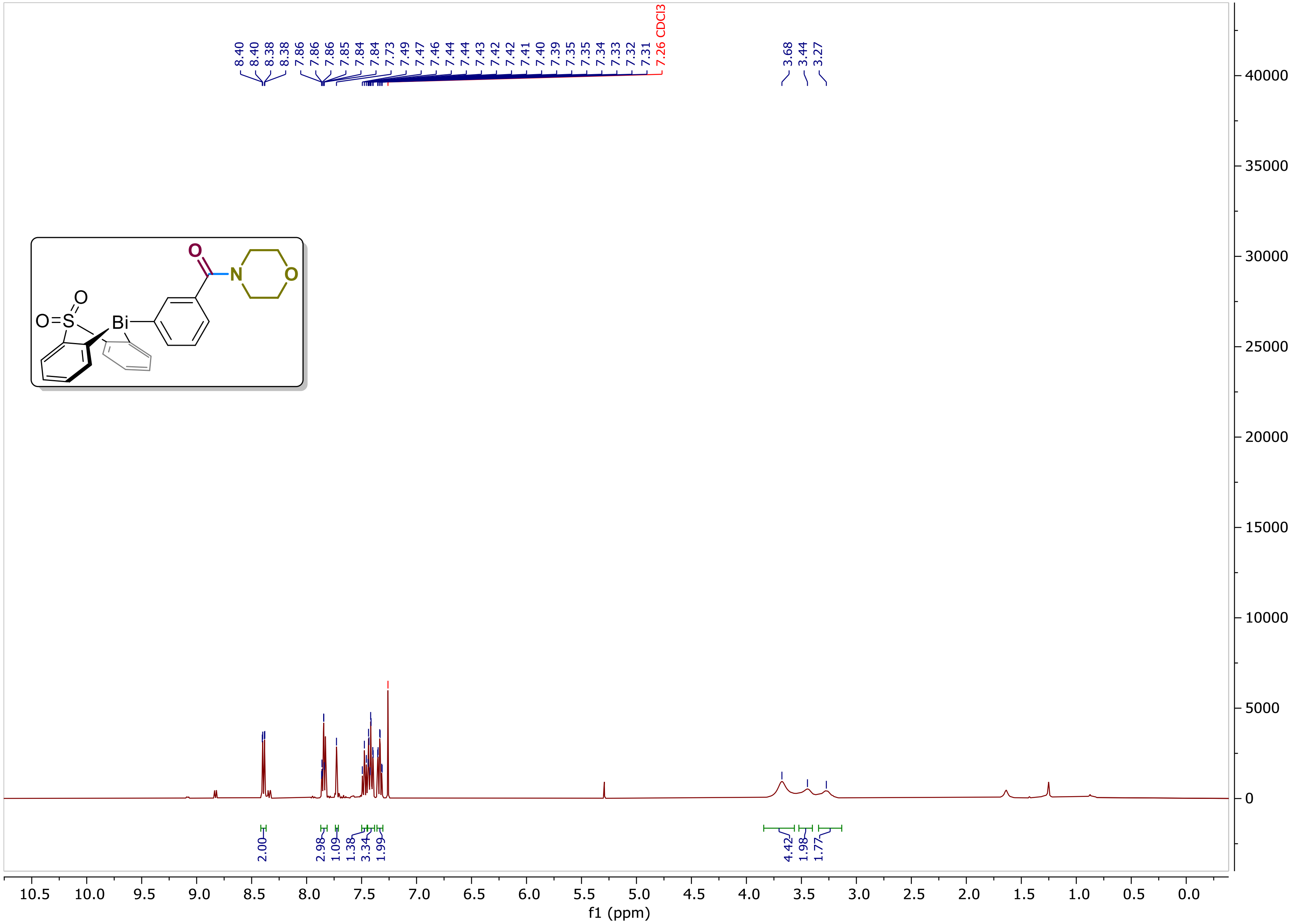

19 - $^{13}C\{^{1}H\}$ NMR (101 MHz, $CDCl_3$):

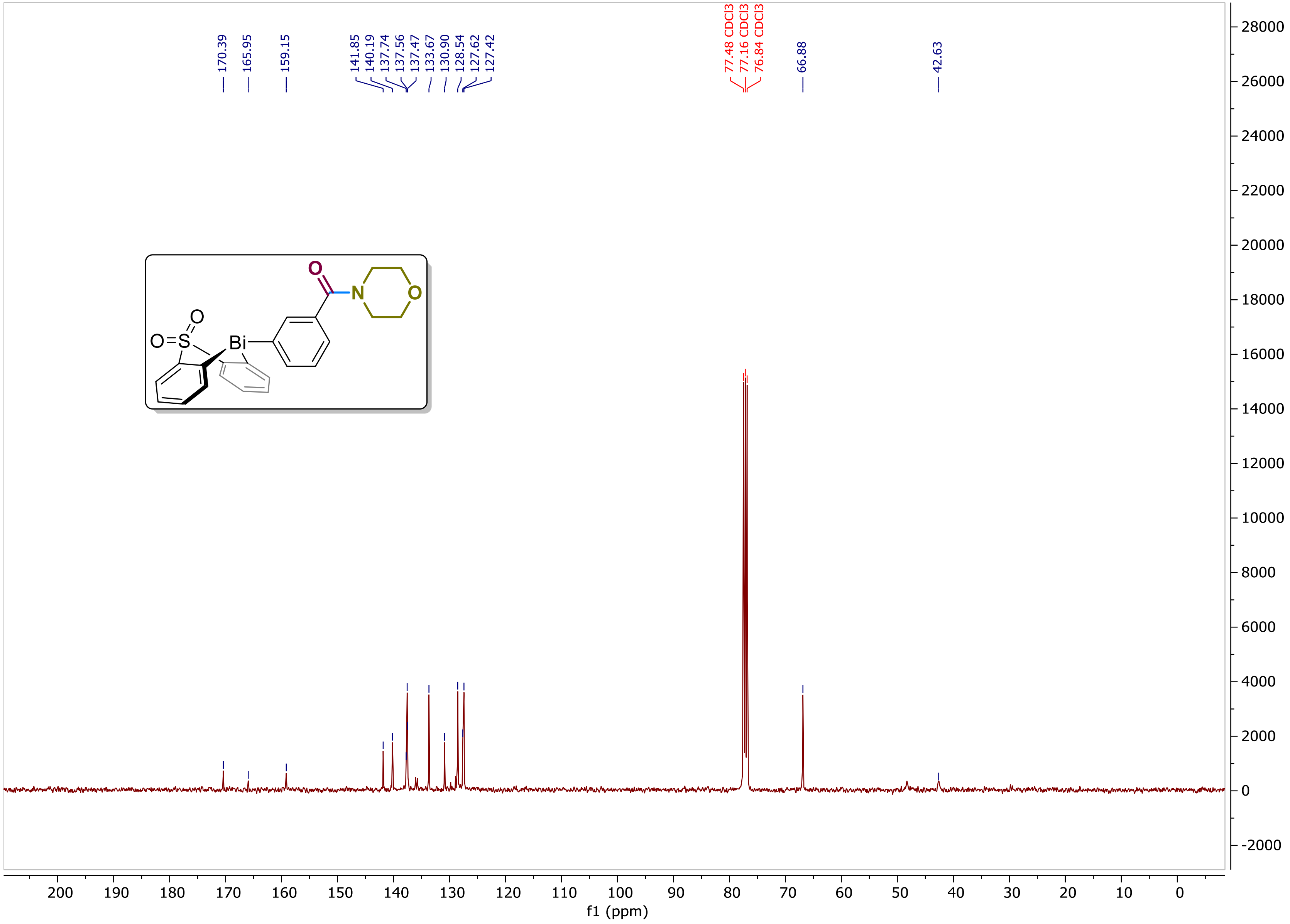

19 - HSQC (400 MHz, $CDCl_3$):

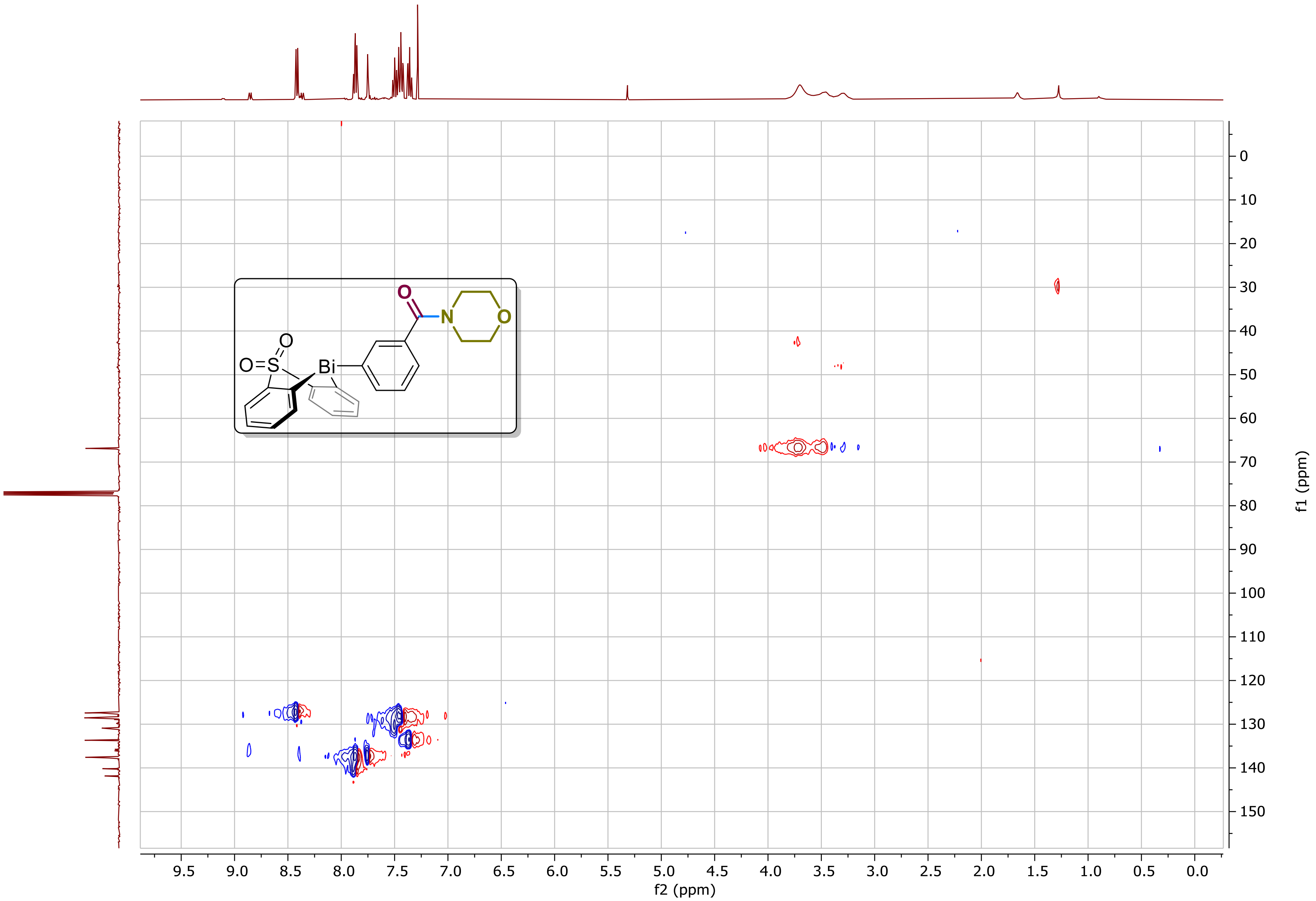

20 - ^{1}H NMR (400 MHz, $CDCl_3$):

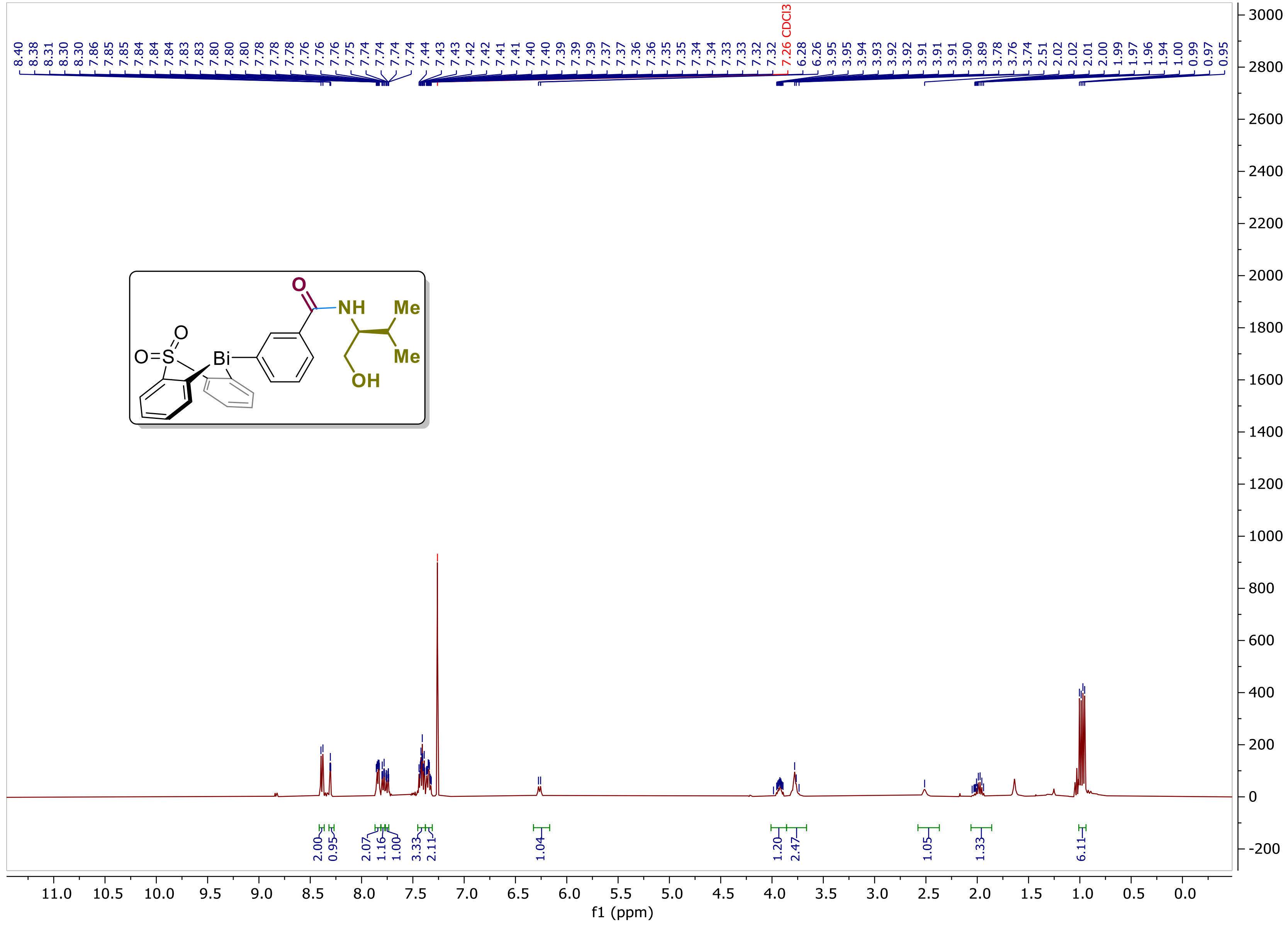

20 - $^{13}C\{^{1}H\}$ NMR (101 MHz, $CDCl_3$):

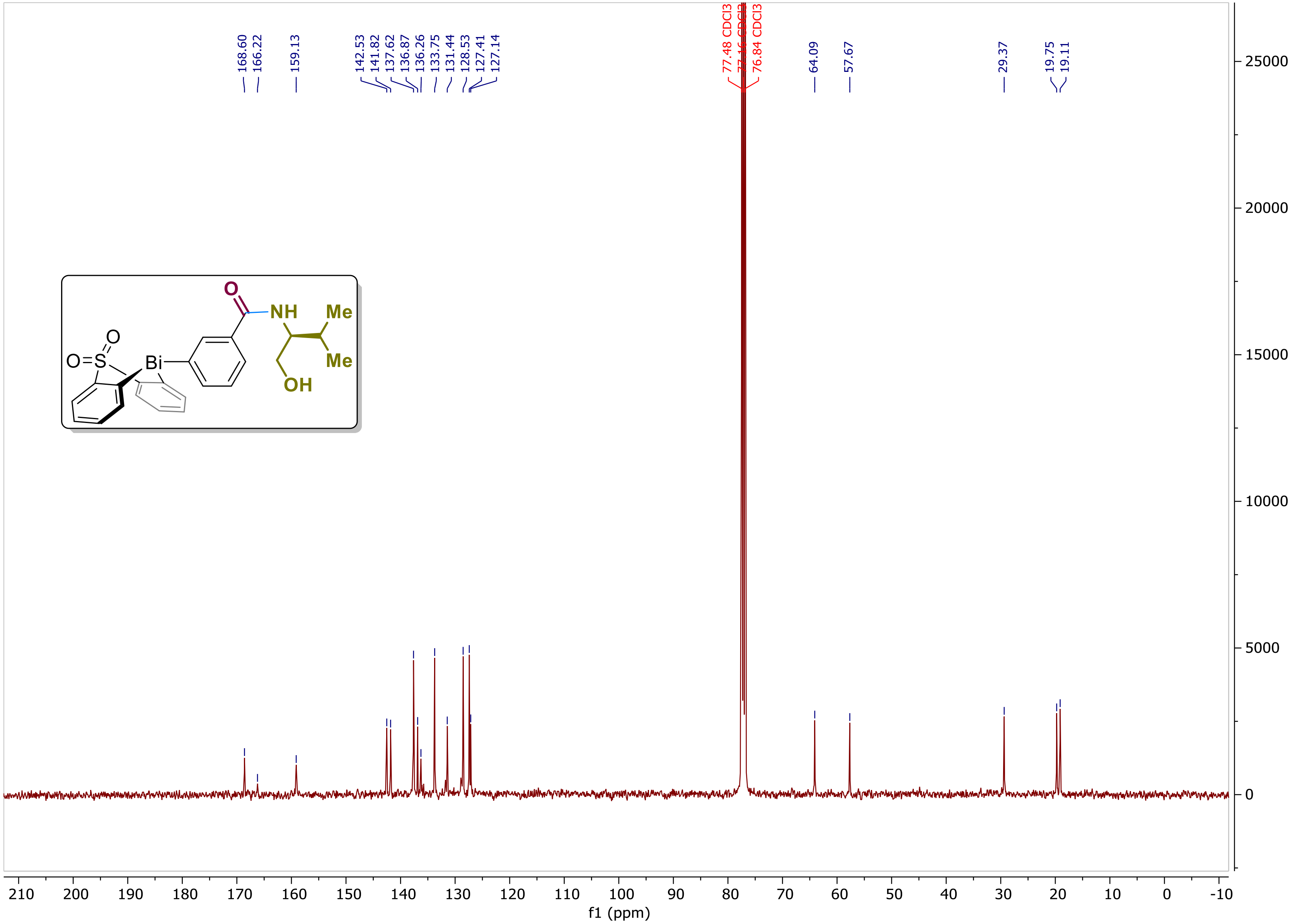

21 - ^{1}H NMR (400 MHz, $CDCl_3$):

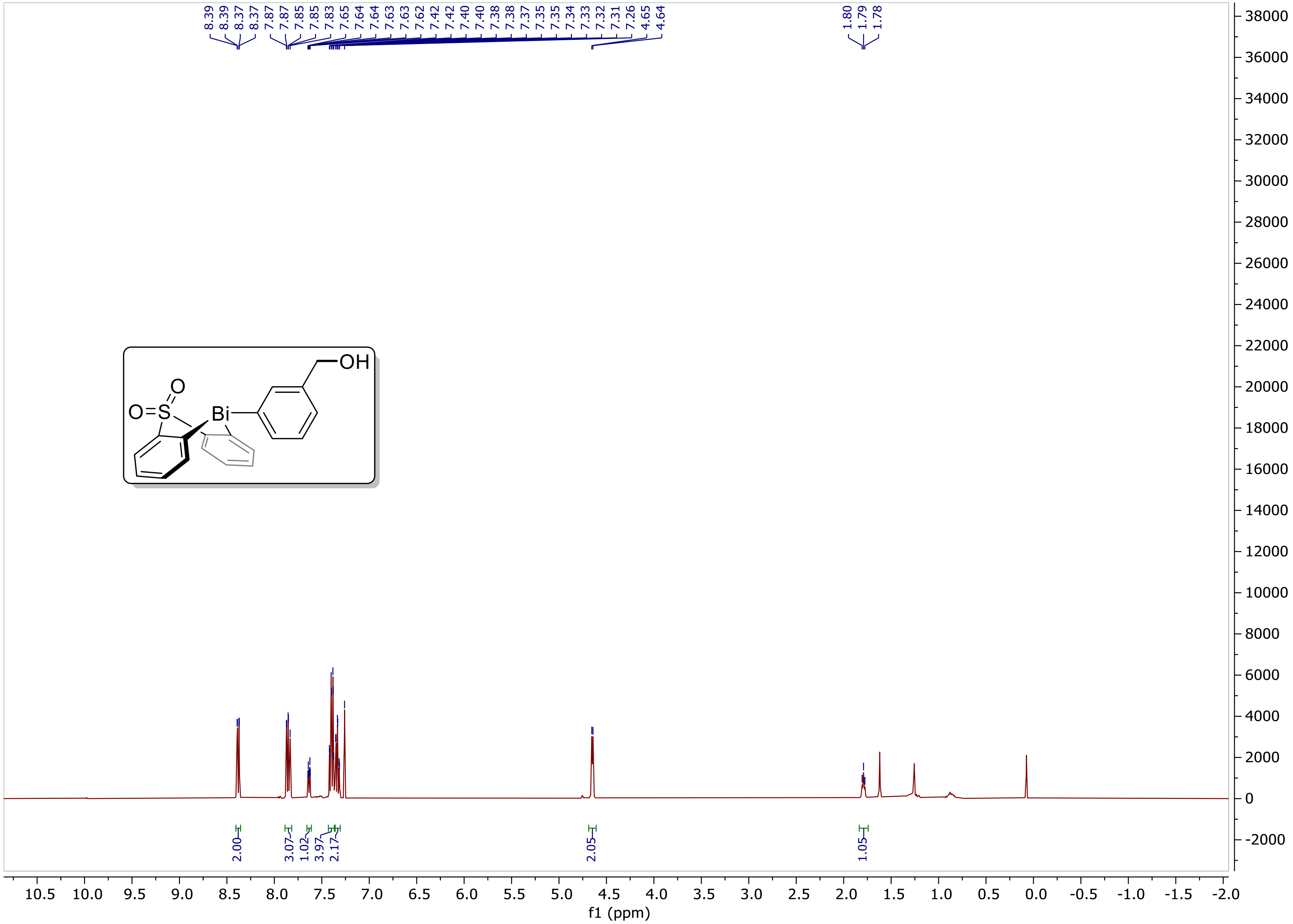

21 - $^{13}C\{^1H\}$ NMR (101 MHz, CDCl$_3$):

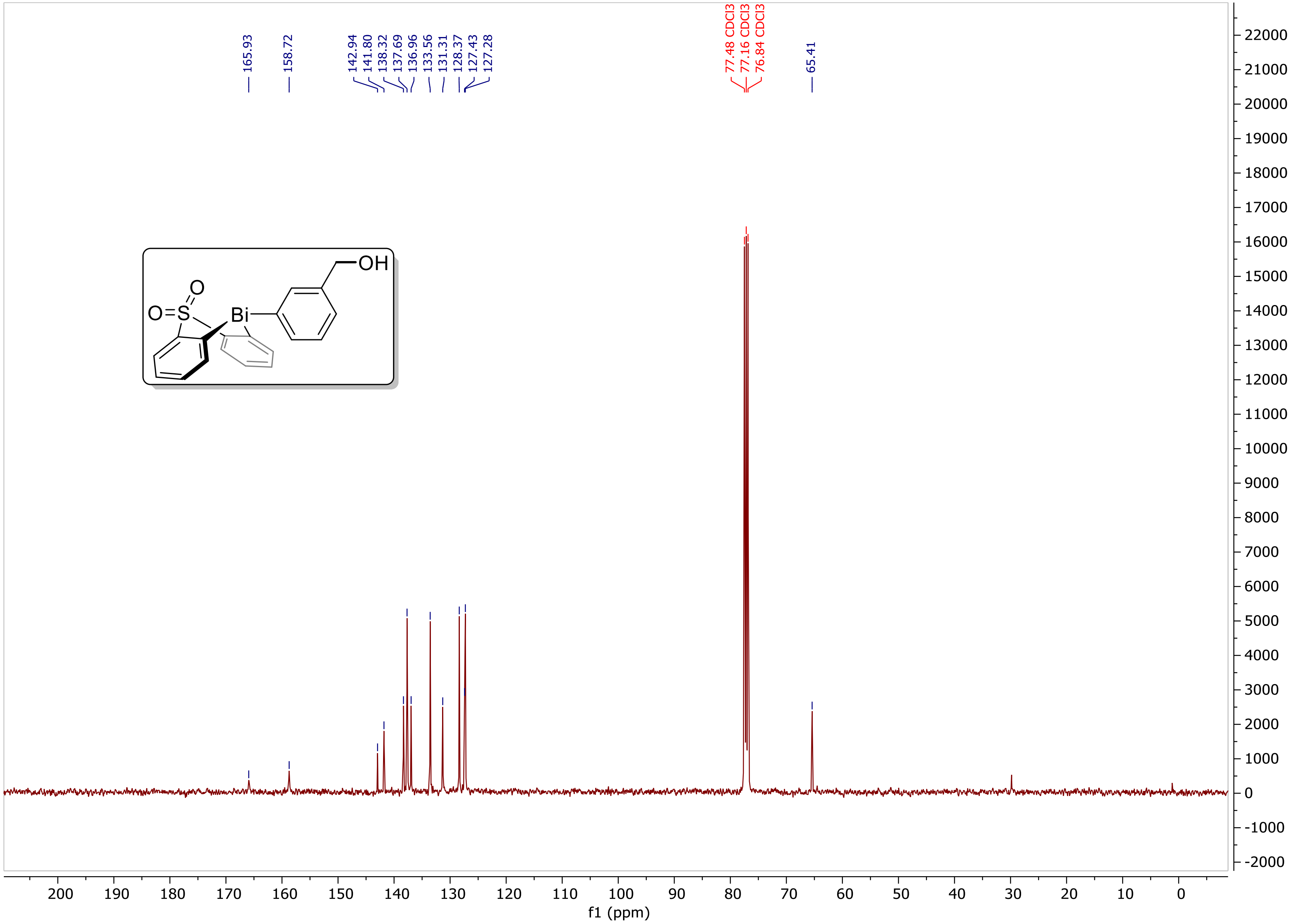

22 - ^{1}H NMR (400 MHz, CDCl$_3$):

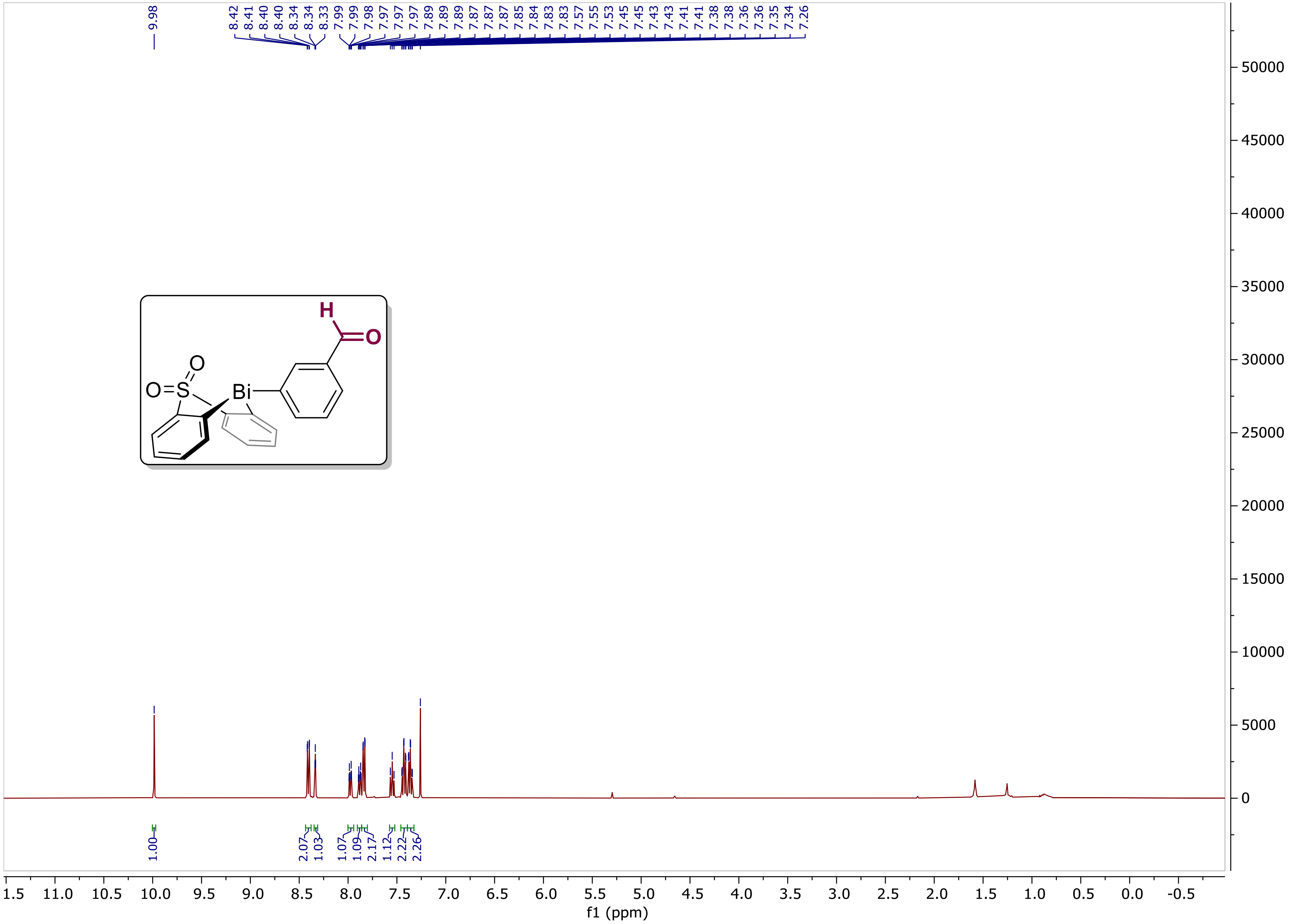

22 - $^{13}C\{^{1}H\}$ NMR (101 MHz, $CDCl_3$):

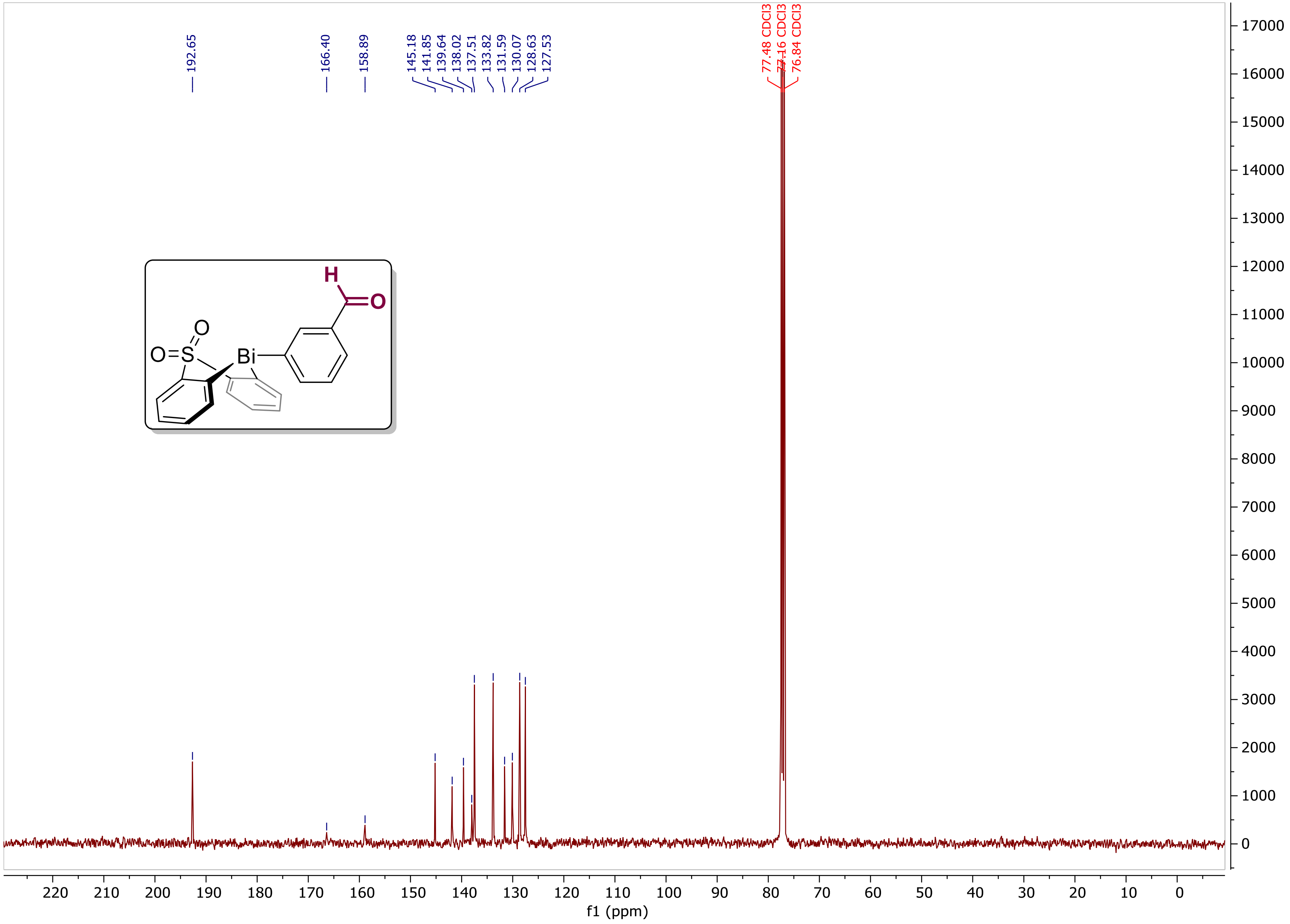

23 - ^{1}H NMR (400 MHz, $CDCl_3$):

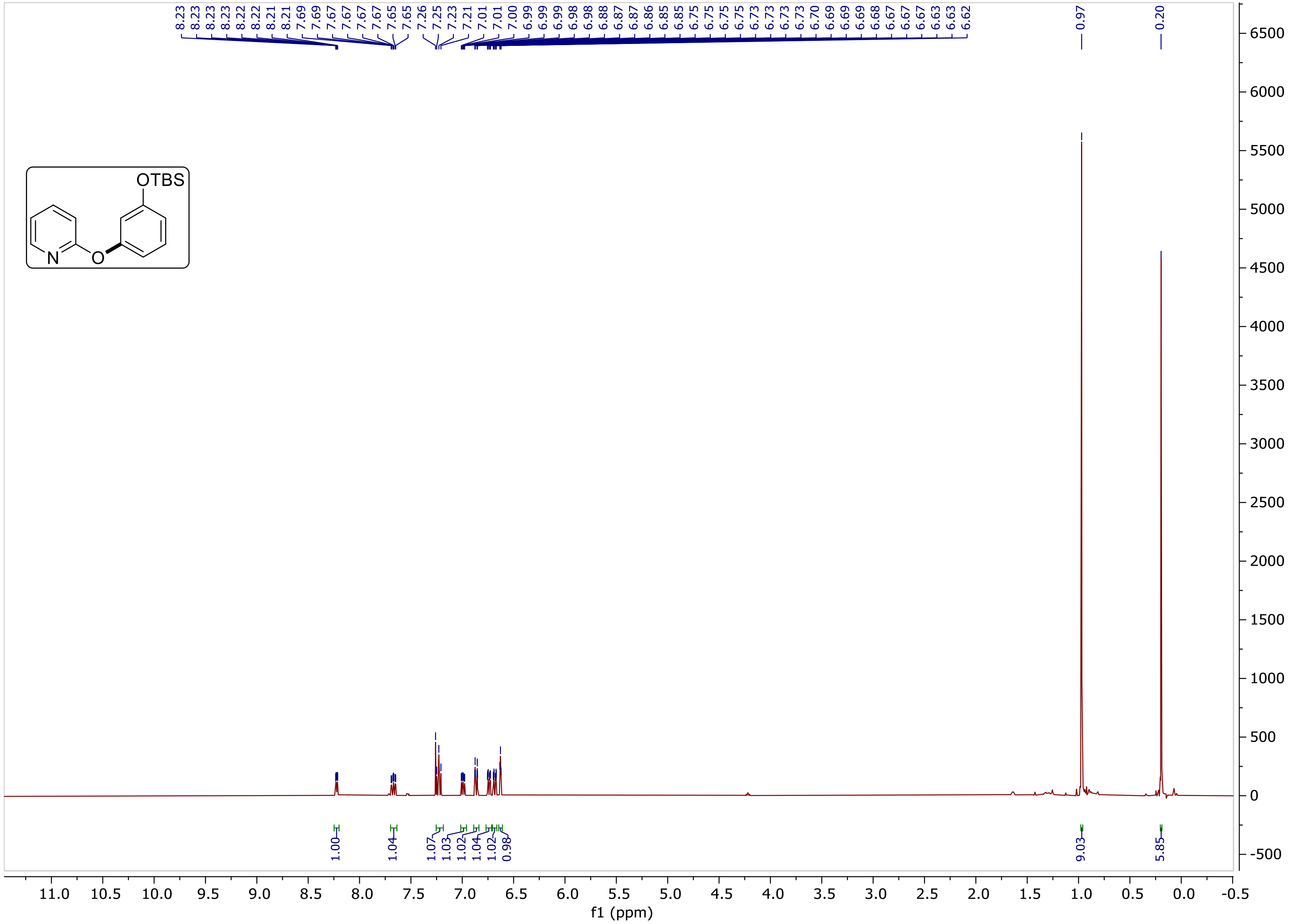

23 - $^{13}C\{^{1}H\}$ NMR (101 MHz, $CDCl_3$):

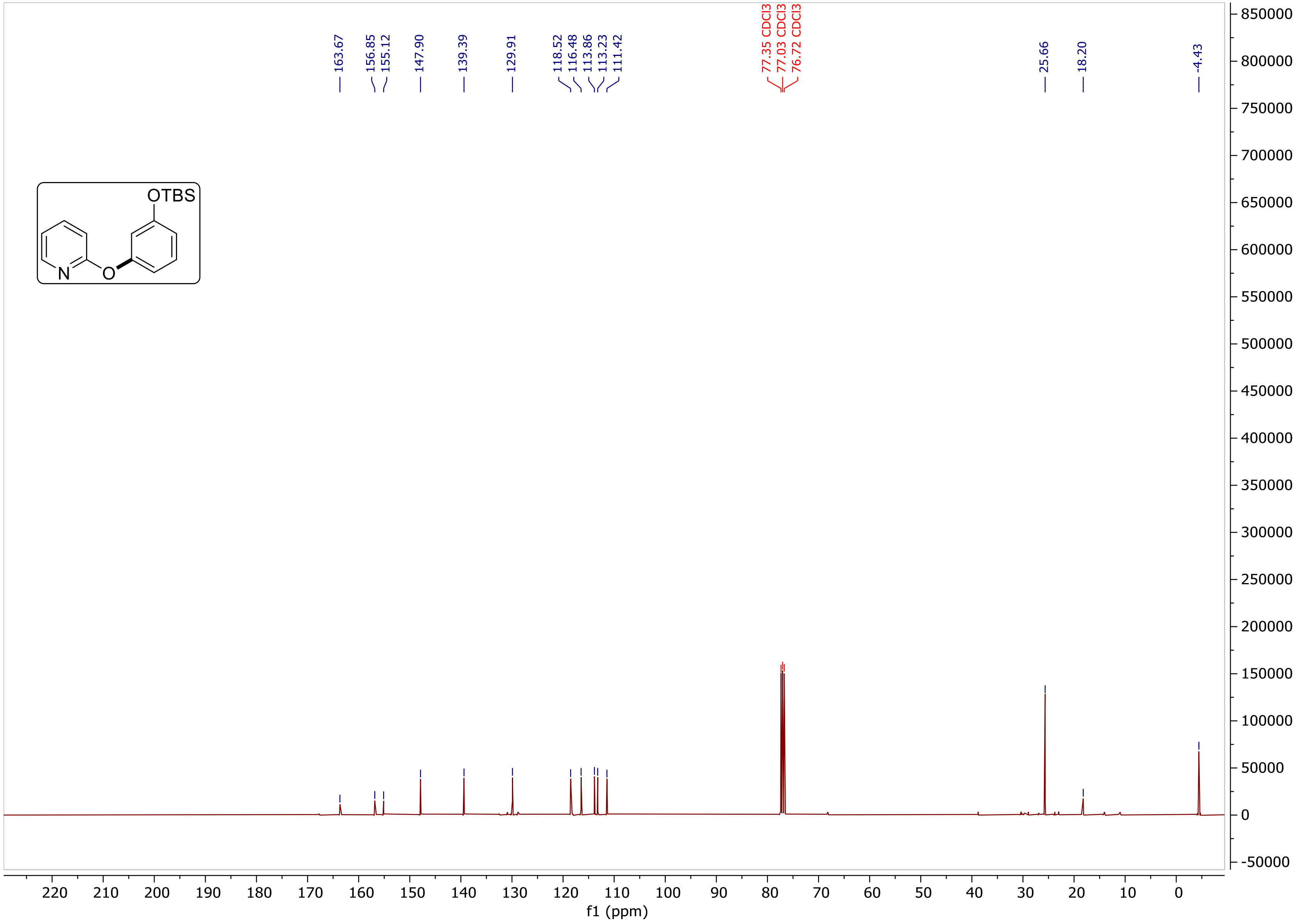

24 - ^{1}H NMR (400 MHz, acetone-d$_6$):

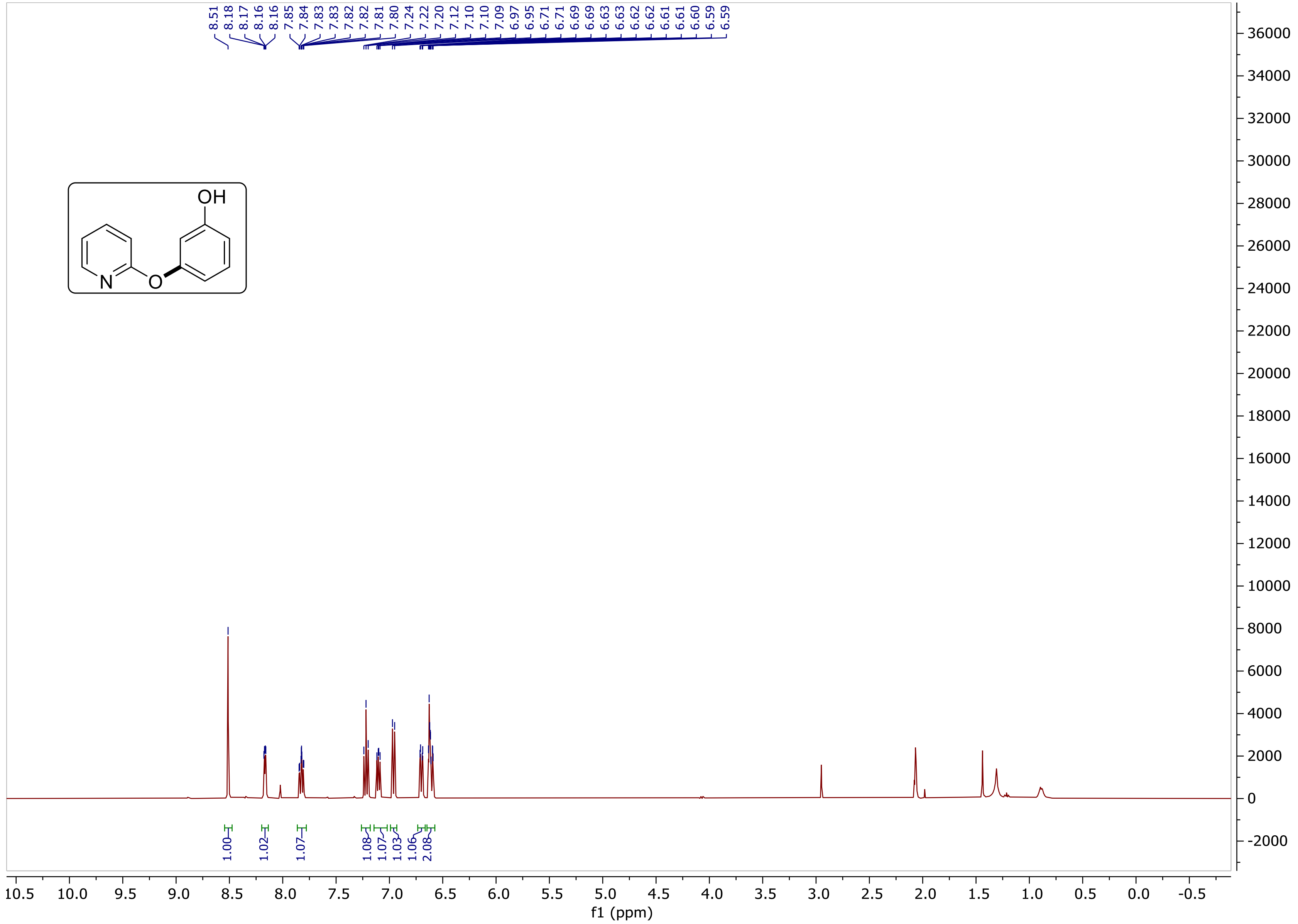

24 - $^{13}C\{^1H\}$ NMR (101 MHz, acetone-d$_6$):

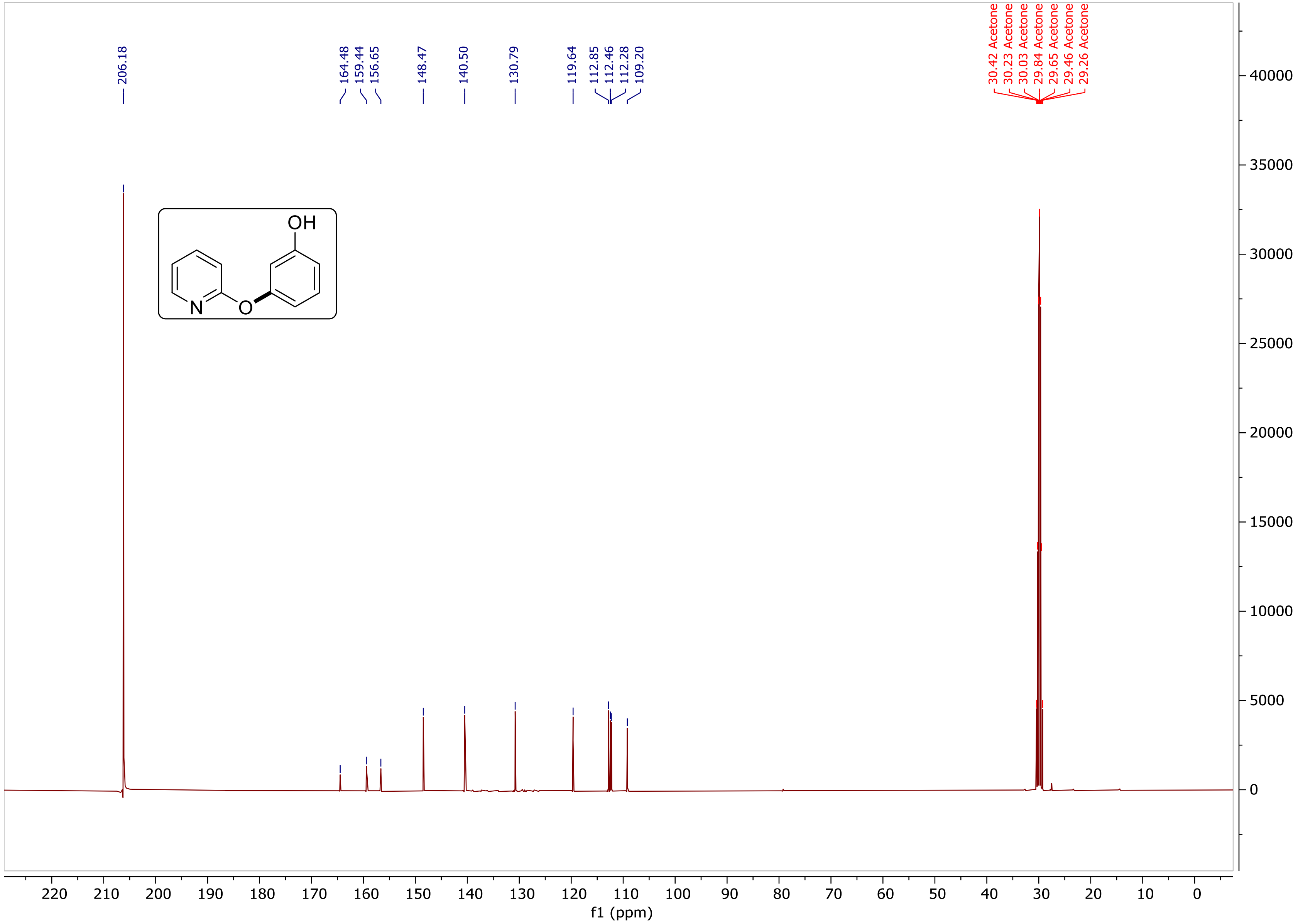

25 - ^{1}H NMR (400 MHz, $CDCl_3$):

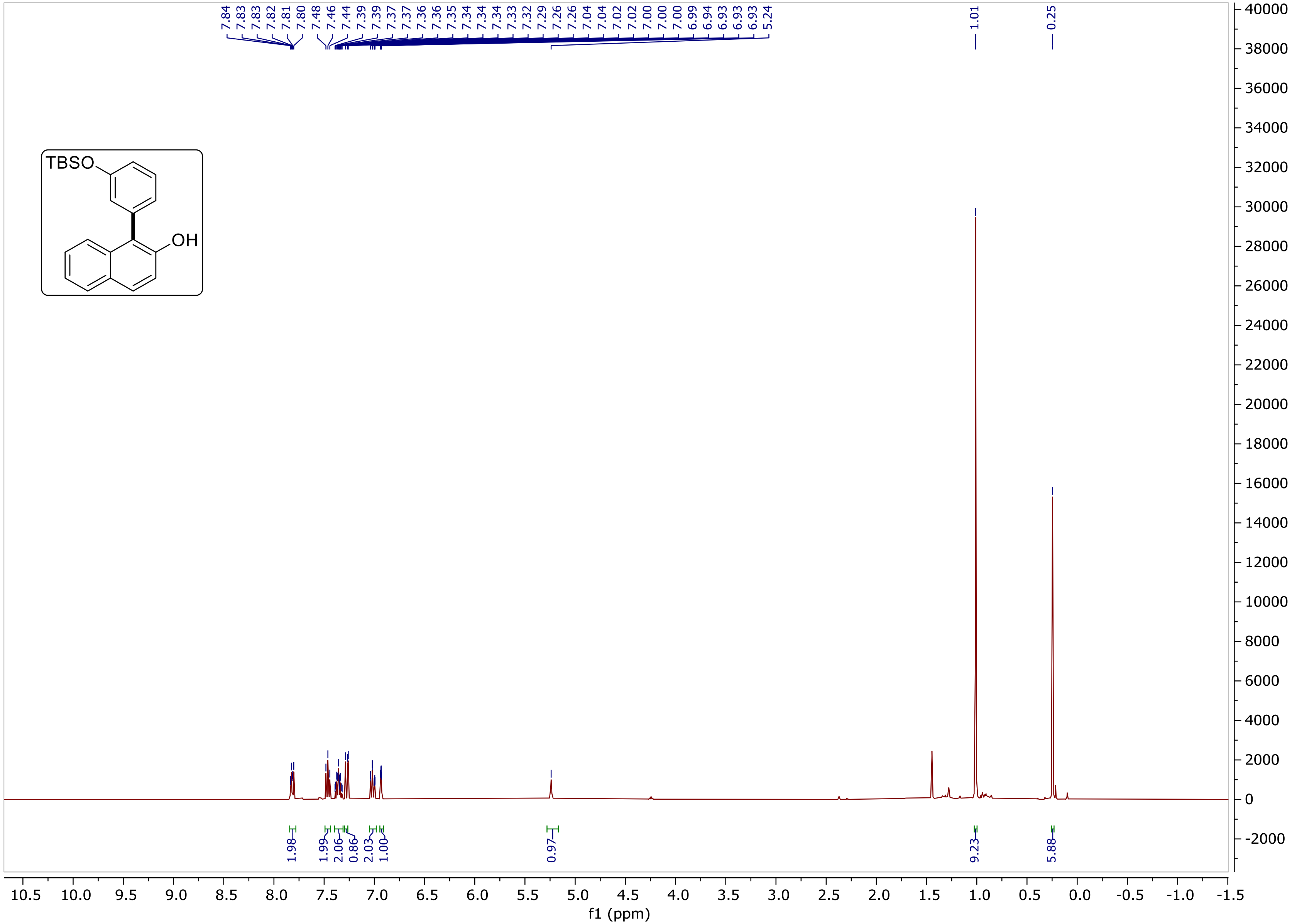

25 - $^{13}C\{^1H\}$ NMR (101 MHz, $CDCl_3$):

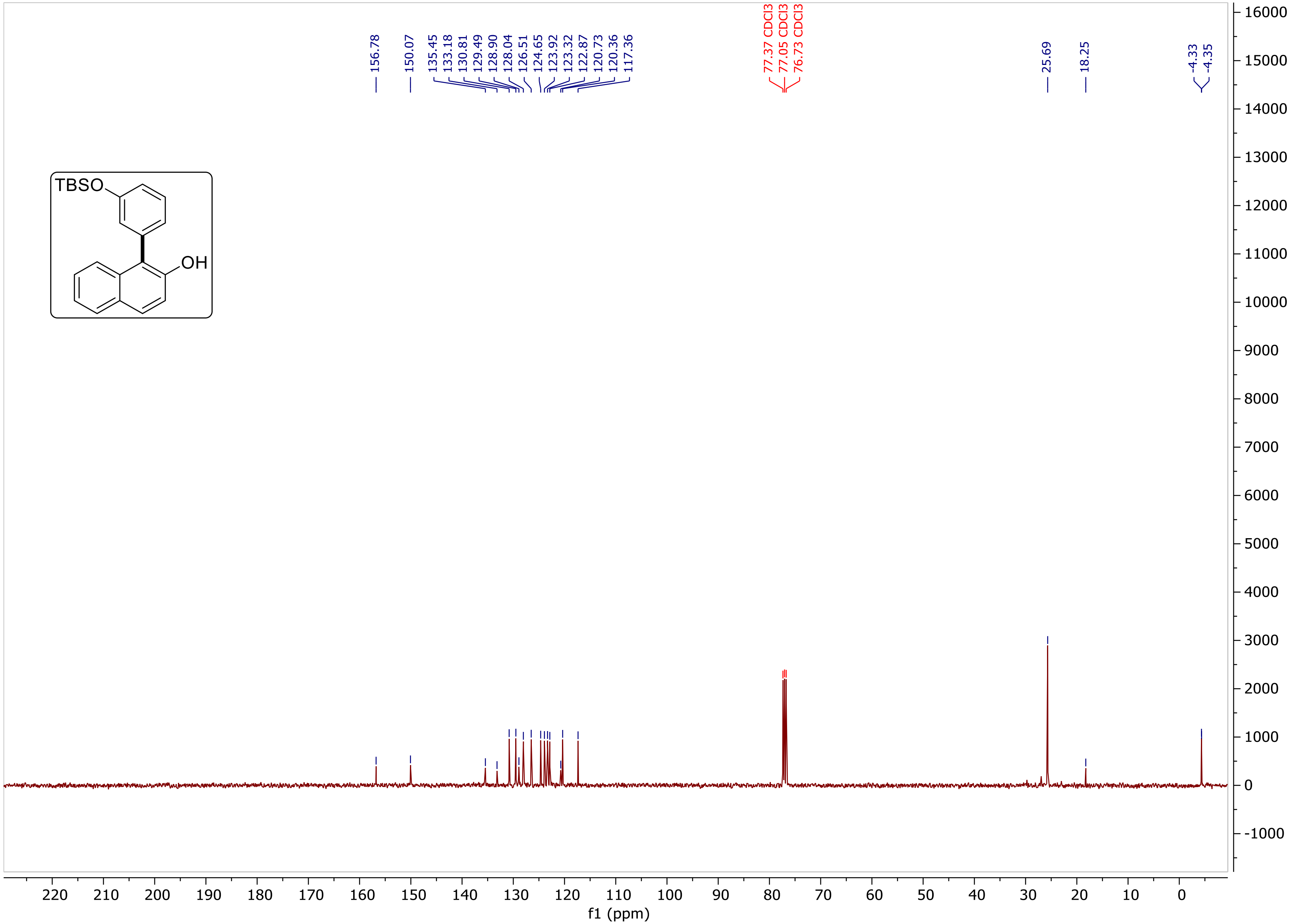

26 - ^{1}H NMR (400 MHz, CDCl$_3$)

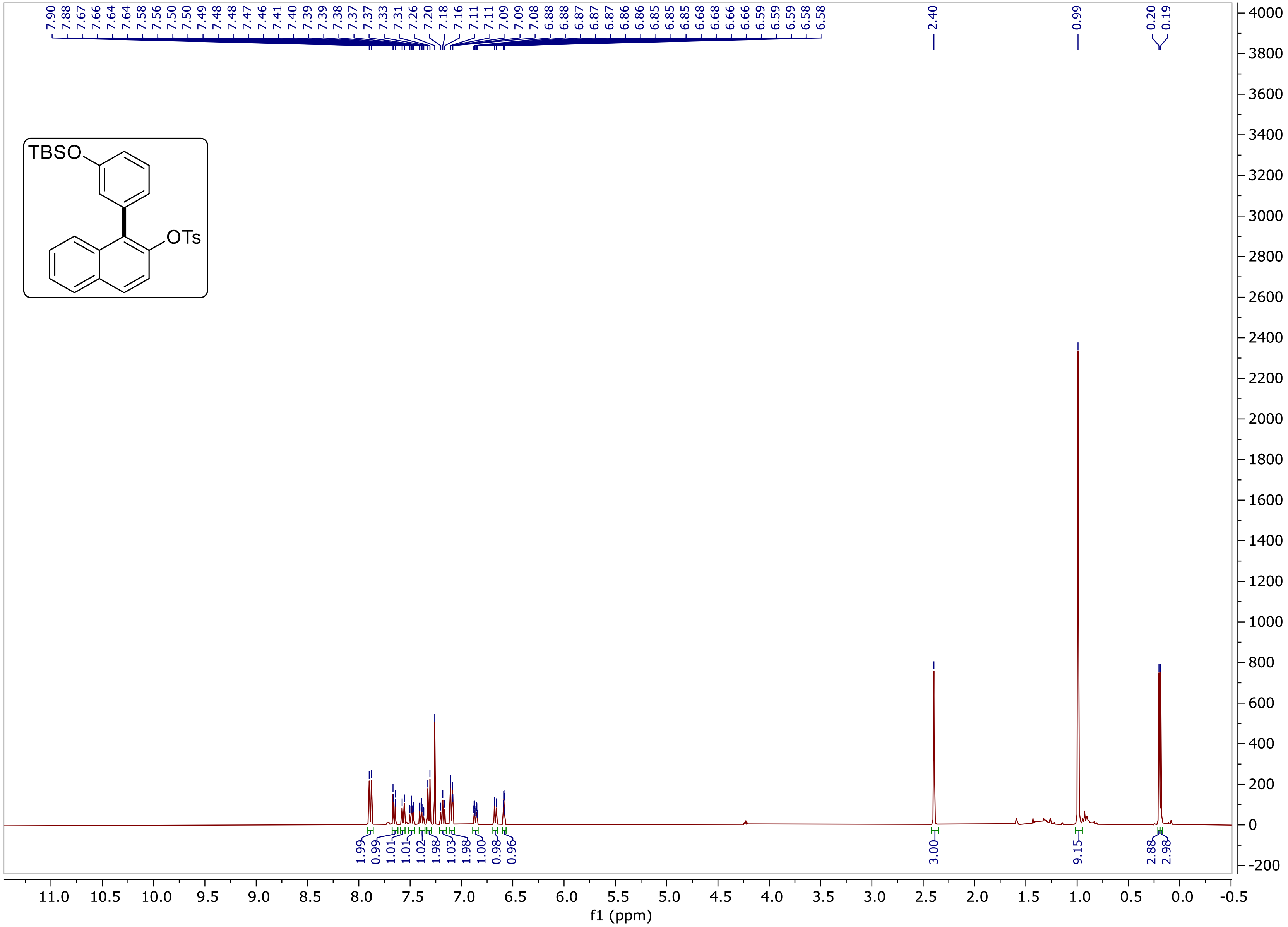

26 - $^{13}C\{^{1}H\}$ NMR (101 MHz, $CDCl_3$):

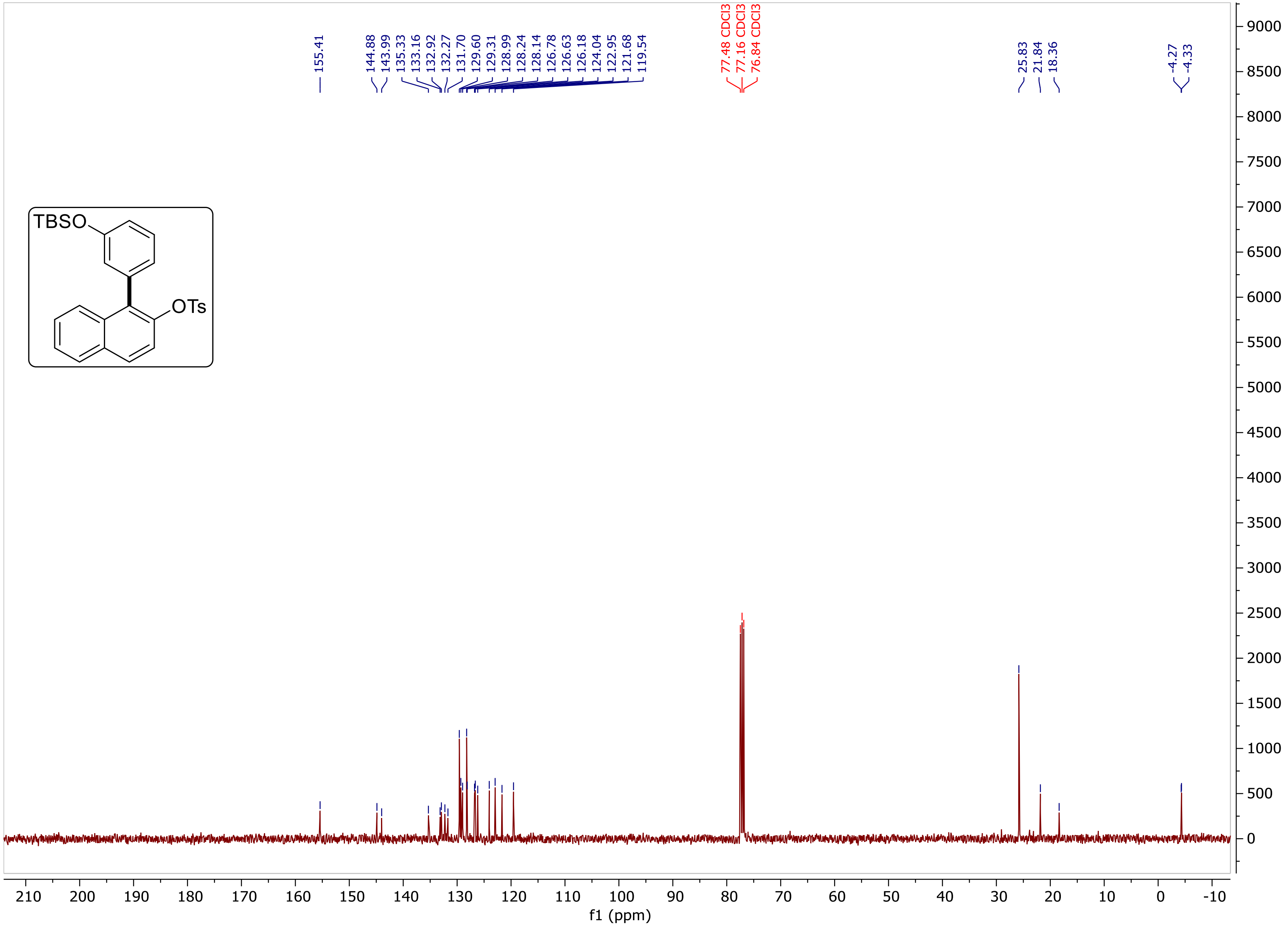

27 - ^{1}H NMR (400 MHz, $CDCl_3$):

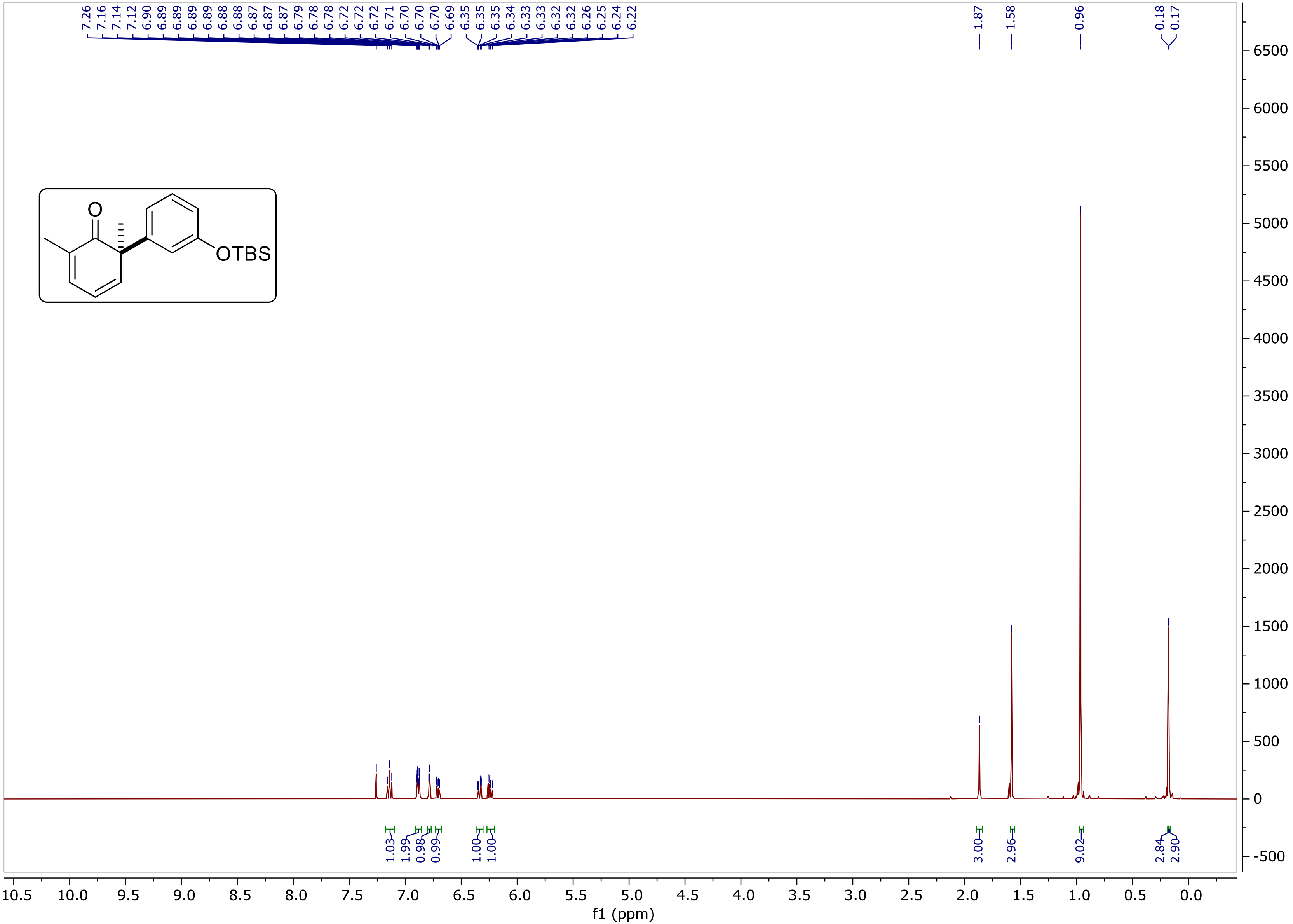

27 - $^{13}C\{^1H\}$ NMR (101 MHz, $CDCl_3$):

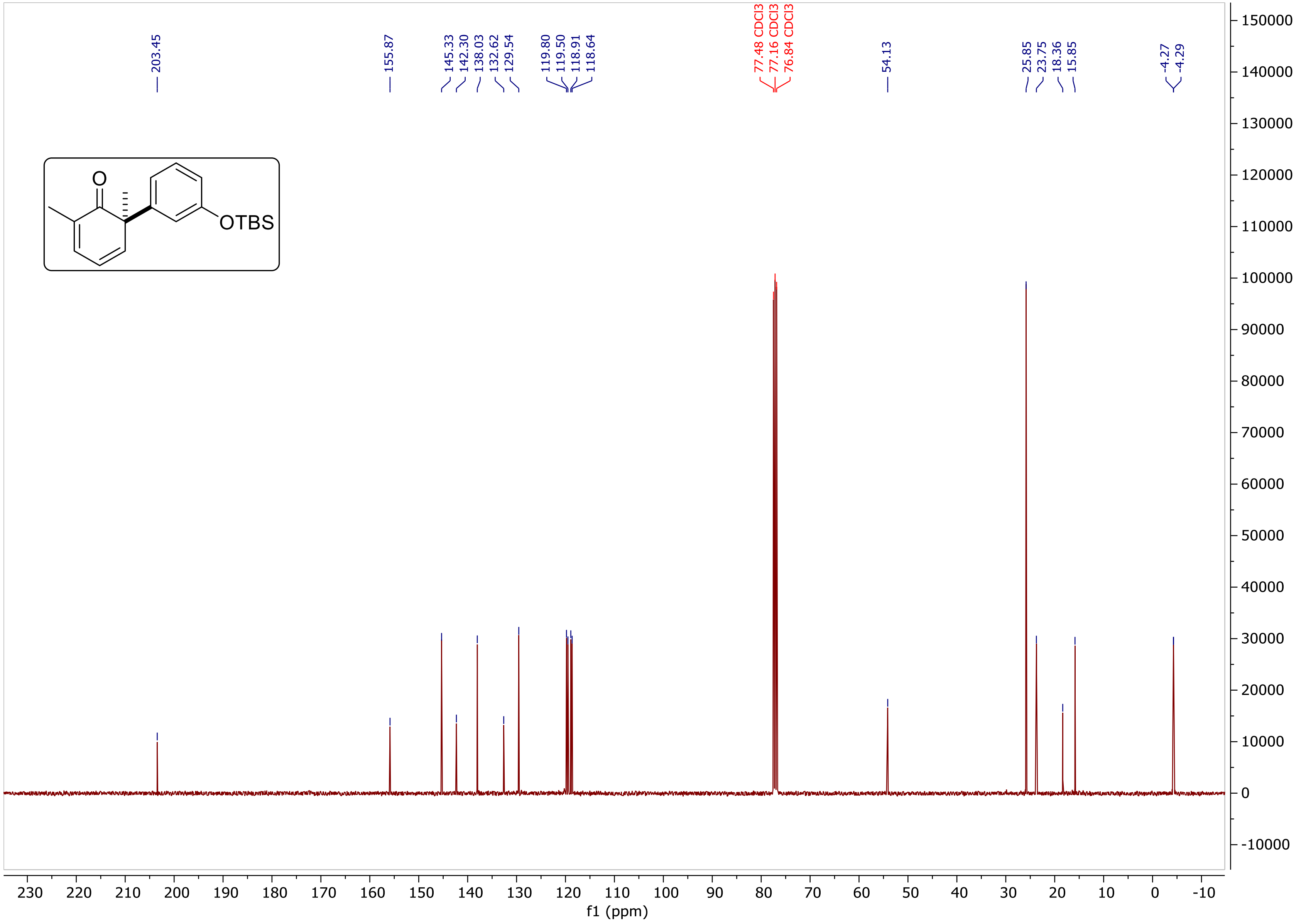

28 - ^{1}H NMR (400 MHz, $CDCl_3$):

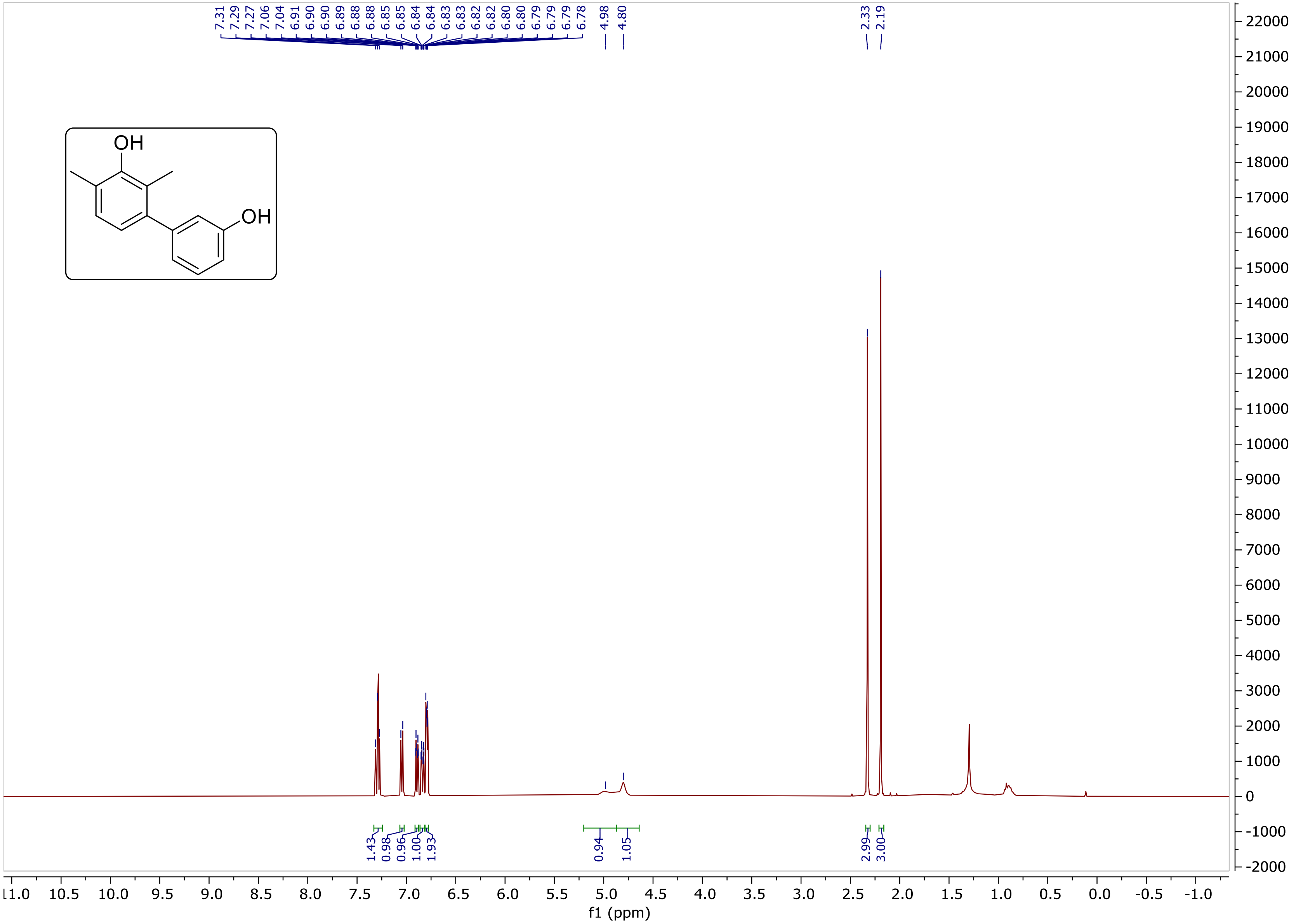

28 - $^{13}C\{^1H\}$ NMR (101 MHz, CDCl$_3$):

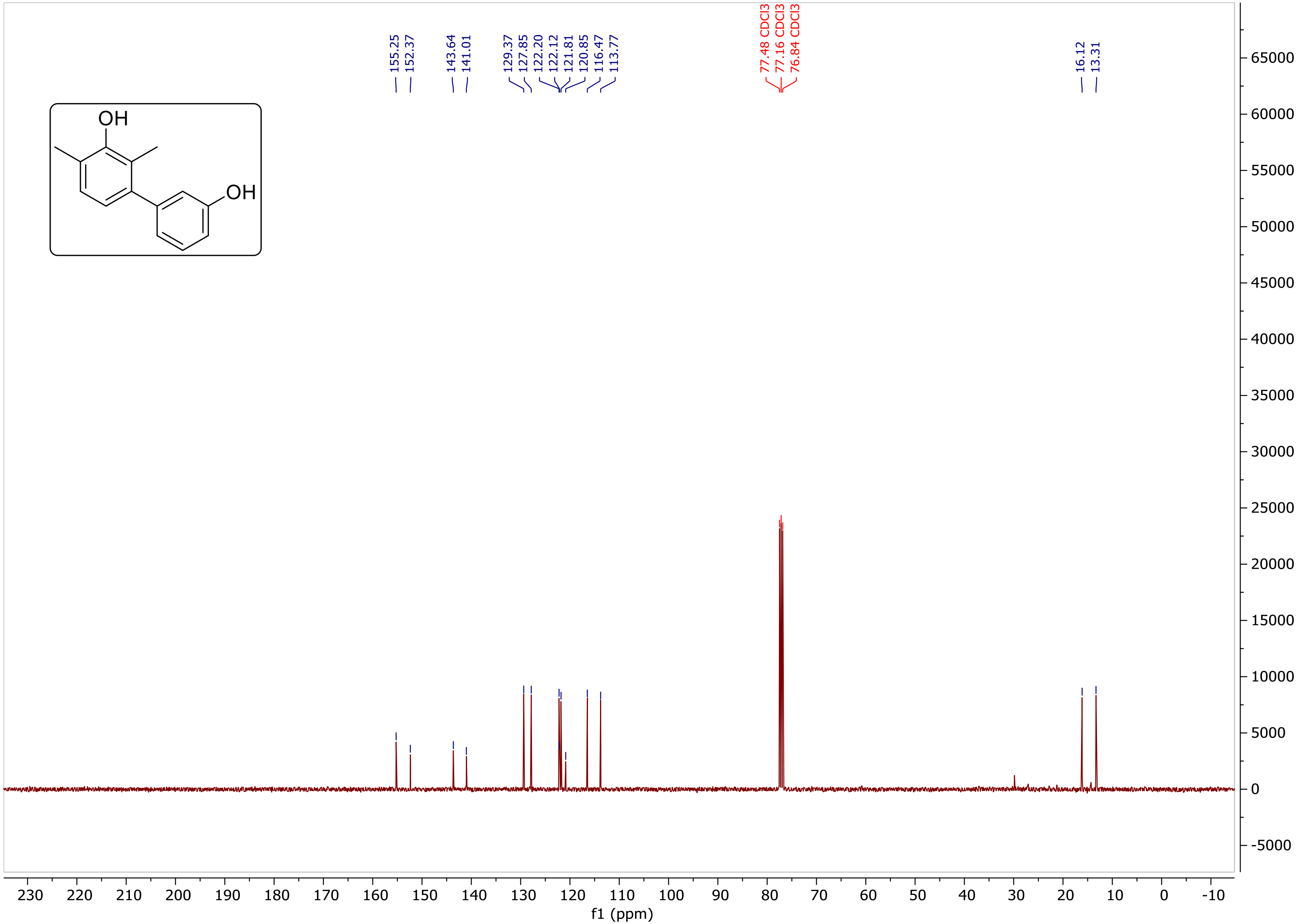

29 - ^{1}H NMR (400 MHz, $CDCl_3$):

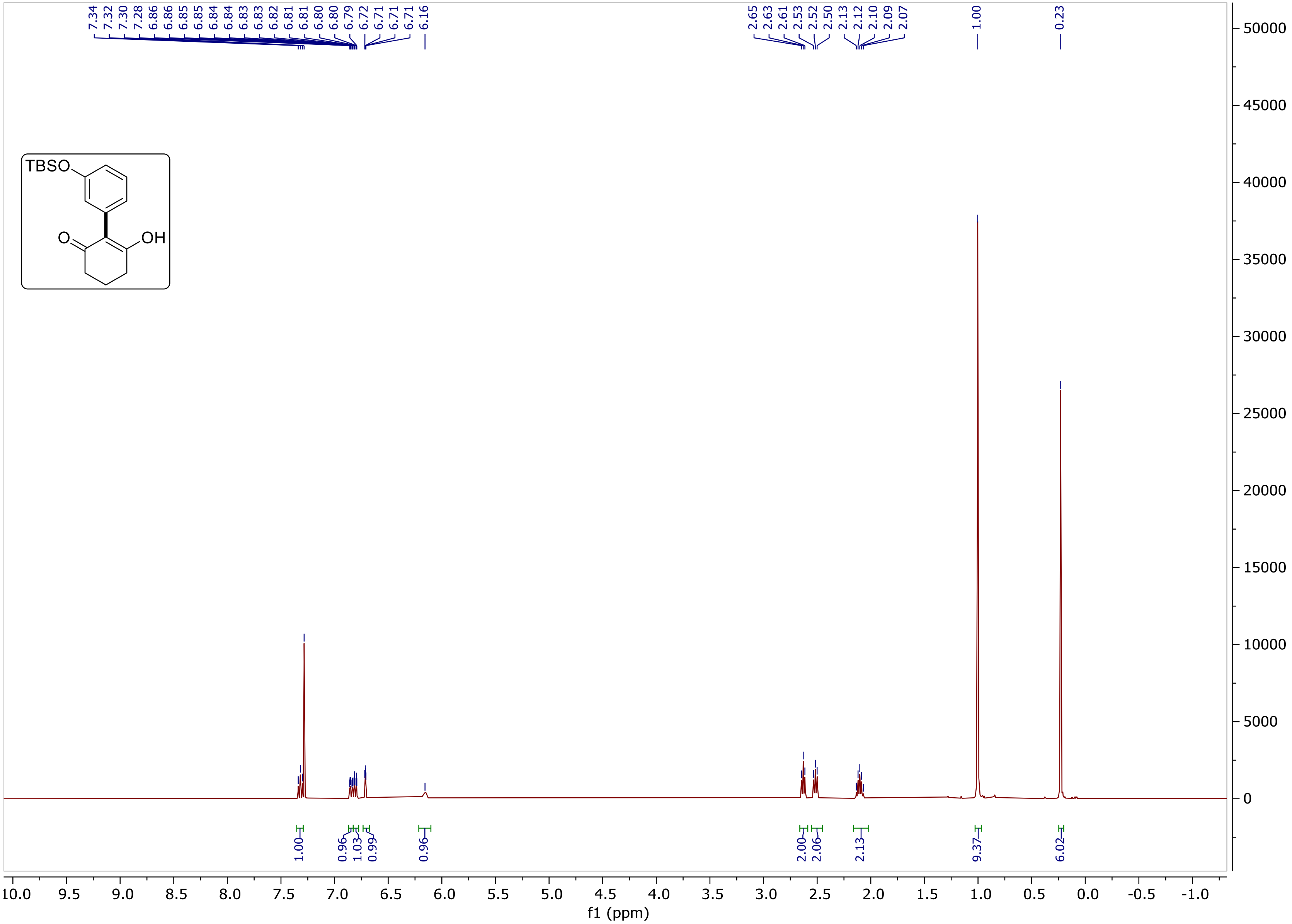

29 - $^{13}C\{^{1}H\}$ NMR (101 MHz, $CDCl_3$):

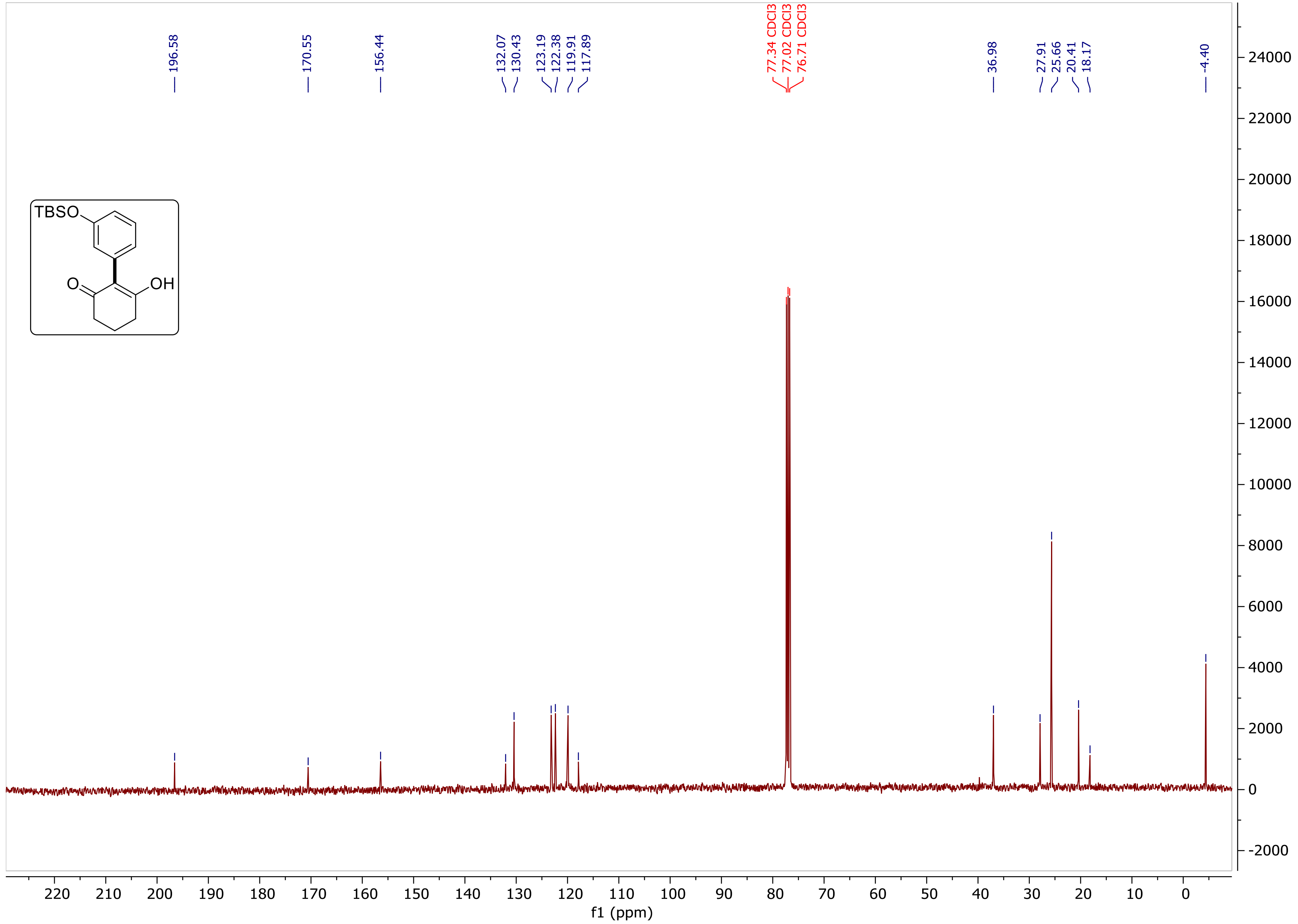

30 - ^{1}H NMR (400 MHz, $CDCl_3$):

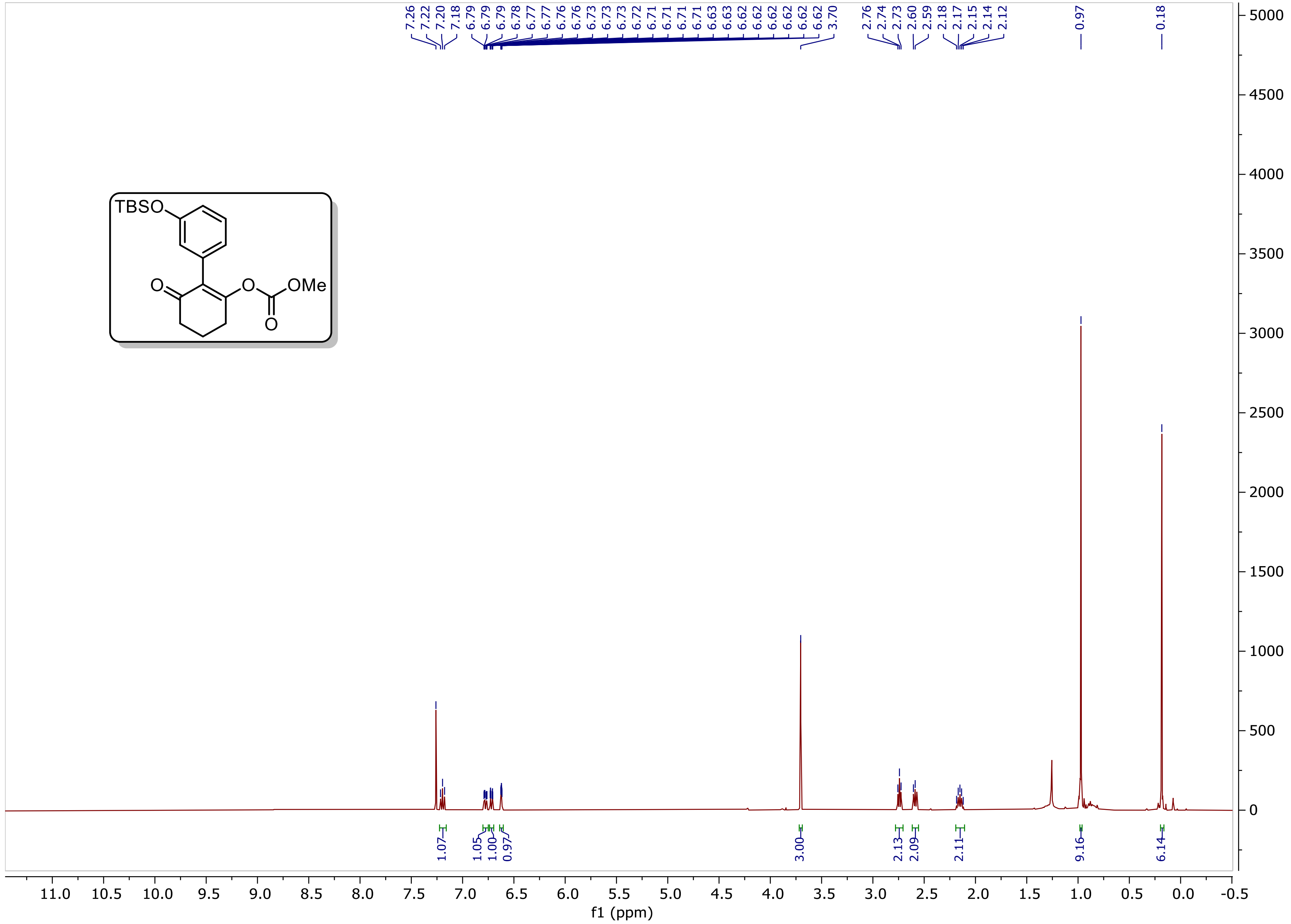

30 - $^{13}C\{^1H\}$ NMR (101 MHz, $CDCl_3$):

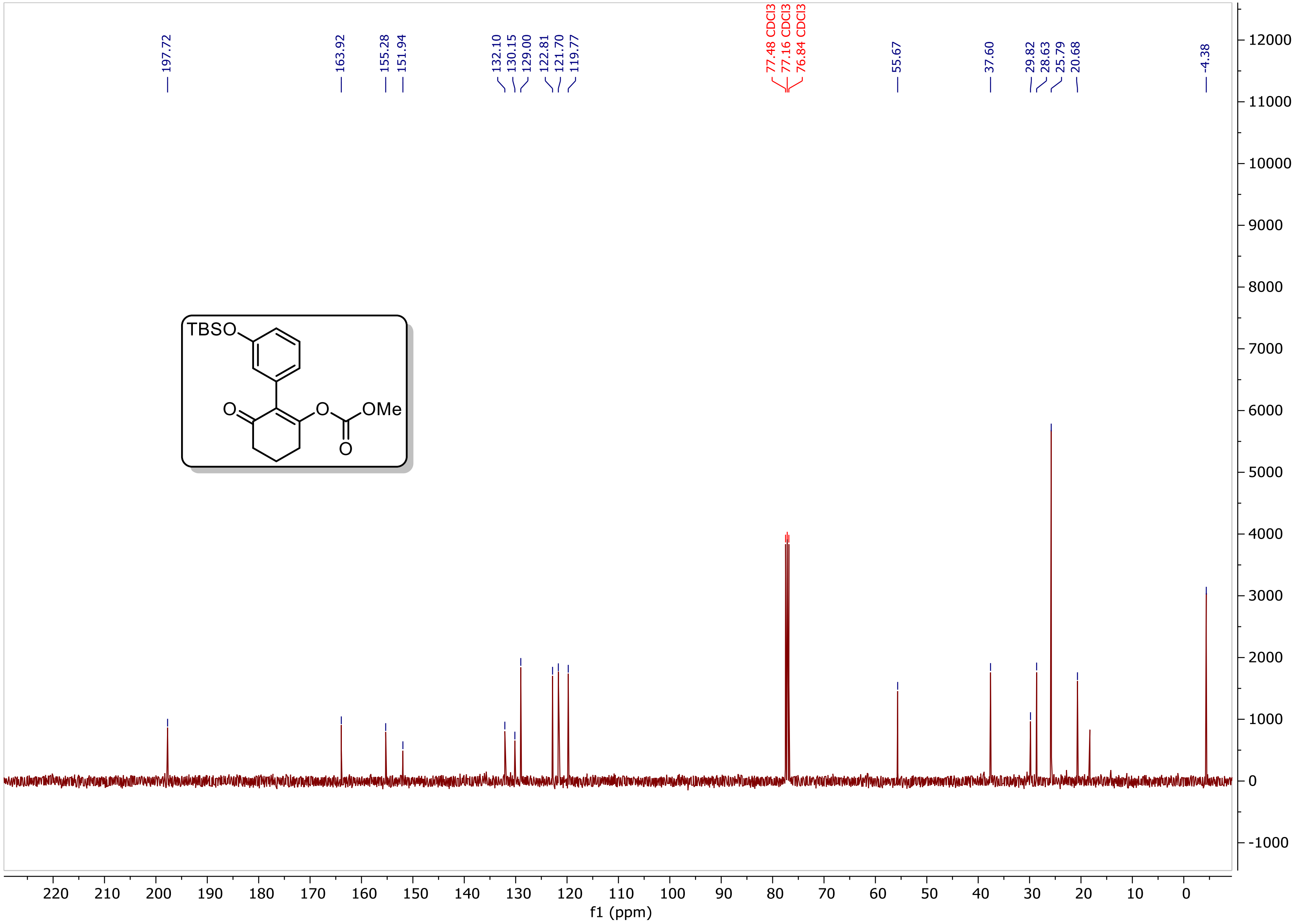

Supplement: Supplementary file 1 — jo3c00361_si_001.pdf [file jo3c00361_si_001.pdf]
